# Higher activation barriers can lift exothermic rate restrictions in electron transfer and enable faster reactions

**DOI:** 10.1038/s41467-018-05267-5

**Published:** 2018-07-25

**Authors:** Kamila K. Mentel, Arménio Serra, Paulo E. Abreu, Luis G. Arnaut

**Affiliations:** 1 Chemistry Department, University of Coimbra, Coimbra, 3004-535 Portugal; 2 Chemical Engineering Department, University of Coimbra, Coimbra, 3030-790 Portugal

## Abstract

Electron transfer reactions are arguably the simplest chemical reactions but they have not yet ceased to intrigue chemists. Charge-separation and charge-recombination reactions are at the core of life-sustaining processes, molecular electronics and solar cells. Intramolecular electron donor-acceptor systems capture the essential features of these reactions and enable their fundamental understanding. Here, we report intramolecular electron transfers covering a range of 100 kcal mol^−1^ in exothermicities that show an increase, then a decrease, and finally an increase in rates with the driving force of the reactions. Concomitantly, apparent activation energies change from positive, to negative and finally to positive. Reactions with positive activation energies are found to be faster than analogous reactions with negative effective activation energies. The increase of the reorganization energy with the driving force of the reactions can explain the peculiar free-energy relationship observed in this work.

## Abstract (web summary)

A widely-assumed principle of chemical reactivity is that, for elementary reactions, higher activation barriers lead to slower reactions. Here, the authors show that some intramolecular electron transfer processes become faster as their apparent activation energies increase, contradicting chemical intuition.

## Introduction

**Fig. 1:**
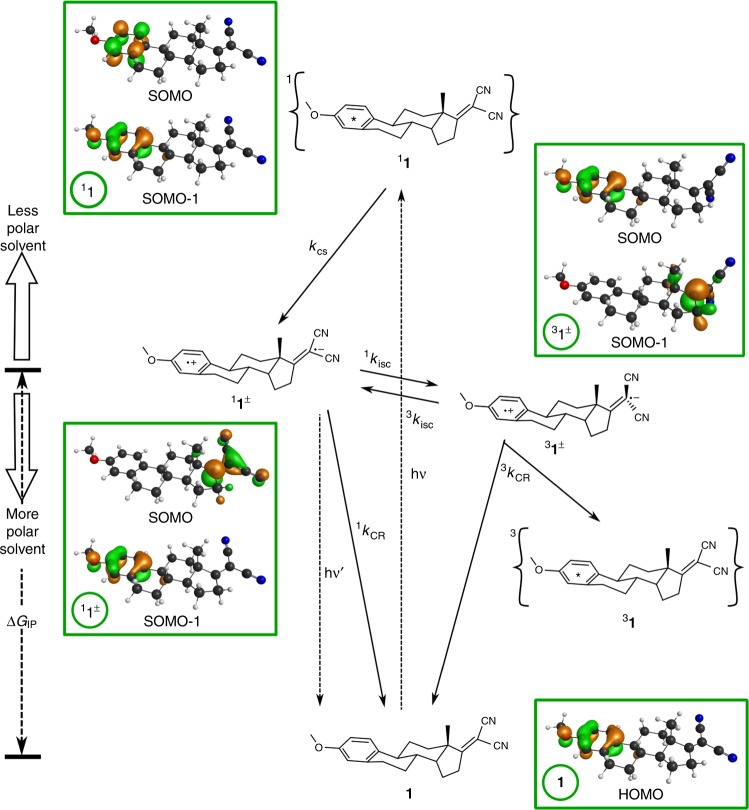
Mechanism of the intramolecular electron transfer reaction. Frontier molecular orbitals of LE and CT states of **1**, and charge separation and recombination rate constants. Molecule **2** is as **1** but without the methoxy group. The vibrational relaxation from the initially populated Franck-Condon state of **1** is omitted for clarity and the first step shown is the ultrafast generation of its lowest singlet state (^**1**^**1**). This is followed by fast charge-separation to a longer-lived CT state (^**1**^**1**^**±**^). Charge recombination of ^**1**^**1**^**±**^ to the ground state (^1^*k*_CR_) may compete with intersystem crossing (^1^*k*_isc_) to the triplet manifold, where ^**3**^**1**^**±**^ may return to ^**1**^**1**^**±**^ (^3^*k*_isc_) or decay to the long-lived triplet state (^**3**^**1**) by triplet charge recombination (^3^*k*_CR_) when ^**3**^**1** is energetically accessible (*E*_T_ < *E*_CT_). The triplet yield of ^**3**^**1** (Φ_T_) is conveniently monitored by flash photolysis in view of its expected microsecond lifetime

Electron transfer (ET) reactions are implicated in fundamental processes, and have been scrutinized since Marcus related ET rates to solvent (*λ*_s_) and molecular vibration (*λ*_v_) reorganization energies^1,2^. Their two most distinctive features are a free-energy dependence characterized by an increase in the rates as their exothermicities increase followed by their decrease for very exothermic reactions (the Marcus “inverted” region)^2^, and the observation of fast rates even when electron donor and acceptor moieties are separated by long and rigid spacers^3,4^. A convenient strategy to trigger ET reactions is electronic excitation, and photoinduced ET reactions have found applications in solar cells^5–7^, organic light-emitting diodes^8^, water splitting^9^, and optoelectronics^10^. Electron donor–acceptor moieties covalently linked by a rigid spacer provide valuable insight on ET reactions because they allow for precise control of both electronic coupling (*V*) and driving force (∆*G*^0^)^11–13^, and are particularly relevant for the fabrication of solar energy conversion and organic electronics devices^14^.The ability to explore the free-energy dependence of very exothermic ET reactions, often believed to be deep in the Marcus inverted region and slow, depends on the design of systems that can be investigated over wide ∆*G*^0^ and *T* ranges without changes in reaction mechanism or electronic coupling. Compounds **1**^15^ and **2** (Fig. 1) have stable and high-energy chromophores based on the benzene ring that act as electron donors in the excited singlet state, covalently linked via a rigid spacer to a stable and optically transparent dicyanoethene moiety working as electron acceptor. Intersystem crossing (isc) may compete with charge separation and recombination in **1** and **2**, and lead to the locally-excited (LE) triplet state. This decay channel is controlled with a D–A distance sufficiently short to enable charge separation (*k*_CS_) and charge recombination (*k*_CR_) rates competitive with intersystems crossing (*k*_isc_) rates, but long enough to allow for proper characterization of charge-transfer (CT) species. The rigidity of these compounds is also critical to provide well-defined reactive species and electronic coupling, and to minimize radiationless processes other than ET.

The rigid donor-spacer-acceptor molecules synthesized in this work were designed to explore the Marcus “inverted” region, the relevance of solvent vs. molecular reorganization energies, and the origin of energy barriers in ET reactions. Our systems enable the control over ET reactions covering 100 kcal/mol in exothermicities and 140 °C in temperature. We show that exothermic rate restrictions have a limit, that dielectric continuum models overestimate *λ*_s_ in weakly and moderately polar solvents (i.e., dielectric constants *ε* between 2.6 and 16), and that the activationless rates in the inverted region are followed by higher rates with positive activation energies (*E*_a_) at higher exothermicities. These findings can be explained by the increase of the reorganization energy with the driving force of the reactions.

## Results

### Density functional theory calculations

DFT calculations on the estrone derivatives **1** and **2** synthesized in this work (see Supplementary Note 1 and Supplementary Figures 1–5) revealed the presence of only one low energy conformer in the ground state of **1**, with a center-to center distance *r*_c_ = 7.8 Å and an edge-to-edge distance *r*_e_ = 5.9 Å (Supplementary Note 2). Figure 1 shows the frontier molecular orbitals of electron donor and acceptor states. Interestingly, the nitrile groups are rotated by 82° in ^**3**^**1**^±^ with respect to ^**1**^**1**^±^, while the C = C bond increases from 1.36 Å in **1** and in ^**1**^**1**, 1.43 Å in ^**1**^**1**^±^ to 1.47 Å in ^**3**^**1**^±^. However, the nitrile groups have very similar orientations in ^**1**^**1**, ^**1**^**1**^±^, and **1**, which means that the twisting of these groups is not involved in the reaction coordinates of singlet state charge separation and recombination but is involved in intersystem crossing to the triplet manifold.

### Energetics

The spectroscopy of ^**1**^**1** gives *E*_S1_ = 99 kcal mol^–1^ (Supplementary Note 3). The triplet energy of ^**3**^**1**, *E*_T1_ = 80 kcal mol^–1^, was estimated from the phosphorescence of dimethylanisole (dMA) and is consistent with the literature value for 1-methoxy-4-methylbenzene, *E*_T1_ = 78 kcal mol^–1^ ^16^. The energies of ^**1**^**1**^**±**^ and ^**3**^**1**^**±**^ depend on the polarity of the solvent. The following weakly polar solvents were studied: di-*n*-butyl ether (NBE), di-isopropyl ether (IPE), ethyl acetate (EAC), chloroform (CHF) and dichloromethane (DCM). The free-energies of ^**1**^**1**^**±**^ CT states were calculated with the Weller expression^17^ using isopropylidenemalononitrile (iPN) as acceptor model, and range from ∆*G*_CT_^0^ = 86 kcal mol^–1^ in NBE to 74 kcal mol^–1^ in DCM (Supplementary Note 4 and Supplementary Tables 2–4). The *o*-xylene (oXY) moiety of **2** has a higher singlet energy (*E*_S1_ = 104 kcal mol^–1^) as well as a higher oxidation potential (*E*_ox_ = 2.09 V vs SCE)^18^, which give ^**1**^**2**^±^ energies ranging from ∆*G*_CT_^0^ = 103 kcal mol^–1^ in NBE to 91 kcal mol^–1^ in DCM, higher than the triplet energy of oXY (*E*_T1_ = 82 kcal mol^–1^)^16^.

### Spectroscopy

The small bathochromic shift in the lowest energy absorption maximum of **1** relative to dMA + iPN, from 283 to 287 nm (Supplementary Fig. 6), reveals the weak coupling between donor and acceptor moieties in the ground state. Similar results were obtained for **2** and oXy + iPN (Supplementary Fig. 7). The slope of the Lippert-Mataga plot (Supplementary Fig. 8) together with the long axis of **1** and the ellipsoidal approximation^15^ afford a dipole moment *μ* = 35 D. A full charge separation with *r*_c_ = 7.8 Å should correspond to *μ* = 37 D. Gamess calculations give *μ* = 37 D. Hence, electronic excitation of **1** in solvents with *ε* > 3 leads to full ET. The slope of the Lippert-Mataga plot of **2** affords *μ* = 26 D (Supplementary Fig. 9), but the CT state ^**1**^**2**^±^ can still be regarded as the result of an ET reaction. More than 95% CT character was deduced for analogous systems in hydrocarbon solvents from the analysis of their radiative charge-recombination rates^19^. This is corroborated by the large separation between LE and CT emissions, the smallest of them is 7,800 cm^–1^ for **2** in NBE, because CT emissions 5000 cm^–1^ lower in energy than the precursor LE emissions indicate more than 90% CT character of the emissive states^20^. Furthermore, the ET process should yield an aryl radical cation with an absorption band at 450–470 nm^21,22^, and such bands were observed for **1** and **2**, as discussed below. Hence, we deduce that the charge separations and charge recombinations measured in this work are essentially ET reactions. The thermochromism of the CT fluorescence is shown in Supplementary Fig. 10. The increase in temperature lowers the dielectric constant of the solvent but decreases the CT fluorescence intensity. This is also consistent with the high CT character of the emissive species because it shows that the radiative processes play a minor role in the decay of the CT states even in lower polarity medium.

### Kinetics

The lowest LE triplet state (^**3**^**1**) in Fig. 1 may be reached via the triplet CT state, ^**1**^**1**^**±**^ → ^**3**^**1**^**±**^ → ^**3**^**1** (Supplementary Note 5). Hyperfine-coupling induced intersystem crossing (hfc-isc) is known to bring the electron spins to a triplet alignment with a rate constant of ≈10^8^ s^–1^ when *r*_c_≈7 Å and the singlet-triplet energy splitting (∆*E*_ST_) is small^23^. In related rigid donor-acceptor systems with 5 CC single bond spacers, the ^1^CT state was calculated to be 0.3 kcal mol^–1^ above that of the ^3^CT state, i.e. ∆*E*_ST_ = 0.3 kcal mol^–1^ ^24^. Alternatively, the rotation of the orbital located in the dicyanoethene moiety when ^**1**^**1**^**±**^ intersystem crosses to ^**3**^**1**^**±**^ may provide the torque for the spin flip. Fast spin-orbit coupling (soc-isc) occurs when the axes of π orbitals of donor and acceptor are perpendicular^25–27^. Both hfc and soc may contribute to ^1^*k*_isc_, and their relative roles do not change the overall mechanism in Fig. 1.

**Fig. 2:**
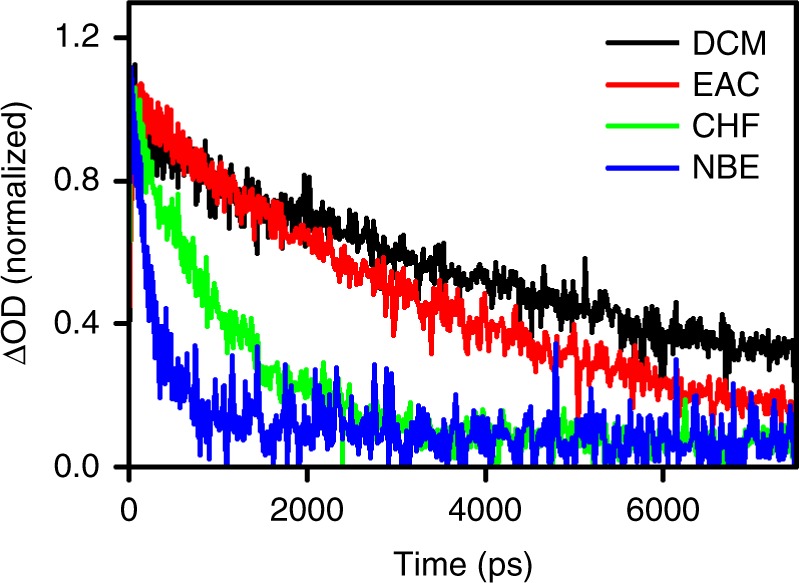
Decays of ^**1**^**2**^±^ at 470 nm in the solvents indicated in the plot. The solvent response was subtracted from decays and the chirp correction was made according to Surface Xplorer, and then the decays were normalized by the maximum absorption change, to be plotted together. Solvents: dichloromethane (DCM), ethyl acetate (EAC), chloroform (CHF), di-*n*-butyl ether (NBE)

**Fig. 3:**
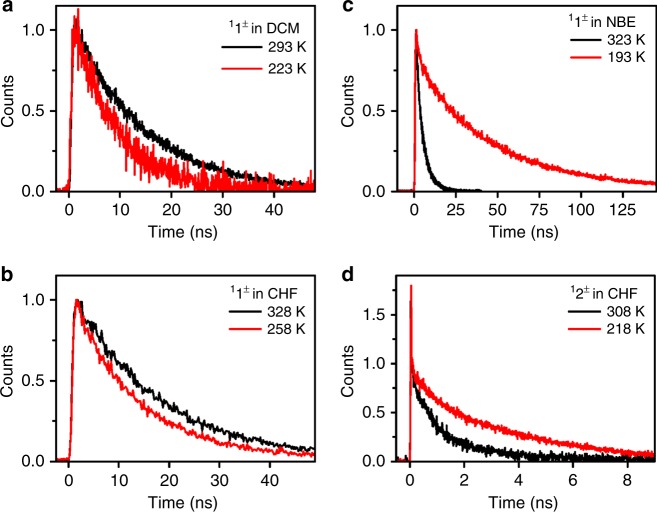
Fluorescence decays. Fluorescence decays at various temperatures of ^**1**^**1**^±^ in dichloromethane (**a**), ^**1**^**1**^±^ in chloroform (**b**) and ^**1**^**1**^±^ in di-*n*-butyl ether (**c**), normalized at maximum, and ^**1**^**2**^±^ in chloroform (**d**) normalized at 100 ps

Figure 2 shows kinetic traces of transient absorption at 470 nm where the aromatic radical cation of ^**1**^**2**^±^ absorbs^21^. The lifetimes of ^**1**^**2**^±^ follow the order *τ*_DCM_ > *τ*_EAC_ > *τ*_CHF_ > *τ*_NBE_, i.e., the fastest charge recombination corresponds to the least polar solvent and most exothermic reaction. This is precisely the opposite of the prediction of Marcus inverted region: the rates should decrease with the increase in driving force. Figure 3 shows fluorescence decays of ^**1**^**1**^±^/DCM and ^**1**^**1**^±^/NBE. The lifetime increases with the temperature in the first system and the decrease in the latter one, which correspond to a change from apparently negative to positive activation energies. The fluorescence decays of ^**1**^**1**^±^ and ^**1**^**2**^±^ in CHF reveal the same phenomenon. The change from *E*_a_ < 0 to *E*_a_ > 0 in the inverted region is unprecedented and leads to the surprising observation that the fastest decay has the highest activation energy.

## Discussion

The essential features of the raw data are better revealed when transient absorption and fluorescence decays are interpreted by target analysis and by the Birks mechanism, respectively (Methods section).

**Fig. 4:**
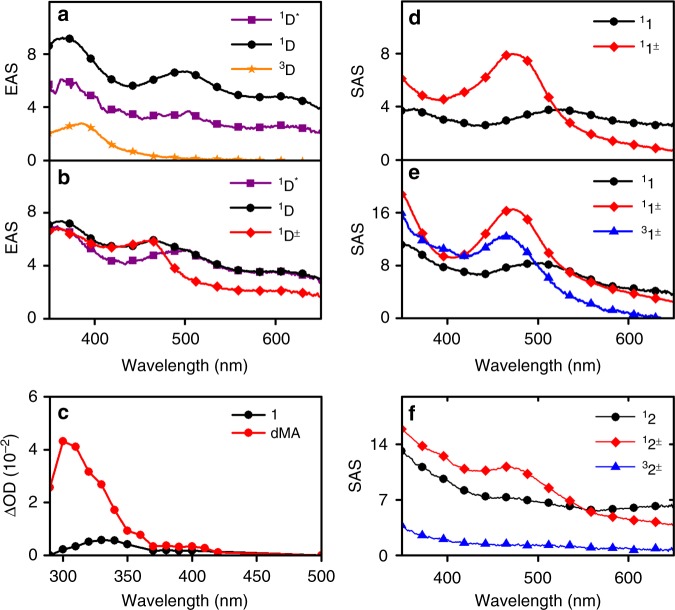
Transient absorption. Evolution-Associated Spectra (EAS) of dMA in the absence (**a**) or presence (**b**) of 0.1 M iPN in isopropyl ether. Flash photolysis of dMA and **1** (**c**) in n-butyl ether illustrating triplet-triplet absorption 100 ns after excitation. Species-Associated Spectra (SAS) of **1** in dichloromethane (**d**) and isopropyl ether (**e**) and of **2** in chloroform (**f**)

Three Evolution-Associated Spectra (EAS) were needed to describe the dynamics of dMA in IPE. The excitation of dMA generates a Franck-Condon state (^1^D*) that relaxes in 1.4 ps to the singlet state (^1^D), which decays in 3.1 ns to leave a persistent species assigned as the triplet state (^3^D). Hence, a kinetic model of three consecutive first-order reactions ^1^D* → ^1^D(→) → ^3^D → is adequate to describe this system. Figure 4 presents the EAS obtained with Glotaran (transient spectra in Supplementary Fig. 10). The quenching of ^1^D* by 0.1 M iPN occurs over a range of distances as the donor and acceptor molecules diffuse in solution. Most meaningful are the shortest-lived EAS, spectroscopically similar to ^1^D*, and the longed-lived one, with a ~460 nm band characteristic of the anisole radical cation^21^.

In addition to the ultrafast lifetime below 1 ps assigned to the decay of the Franck-Condon state, two lifetimes between 1 ps and 20 ns are necessary to fit transient absorption spectra of **1** in DCM, EAC and CHF, and three lifetimes are needed for ethers at room temperature, as well as for **2** in all solvents. Target analysis using the model of Fig. 1 gives the Species-Associated Spectra (SAS) shown in Fig. 4. A species with a spectrum similar to that of ^1^D and a lifetime between 4 and 10 ps was assigned to ^**1**^**1**. A species generated from ^**1**^**1** and a band at 472–477 nm was assigned to ^**1**^**1**^**±**^. When an additional species was found necessary to fit the spectra, its spectrum was similar to that of ^**1**^**1**^**±**^ and assigned to ^**3**^**1**^**±**^. Analogous assignments were made for **2**. Supplementary Tables 5–6 present the lifetimes obtained by target analysis.

**Fig. 5:**
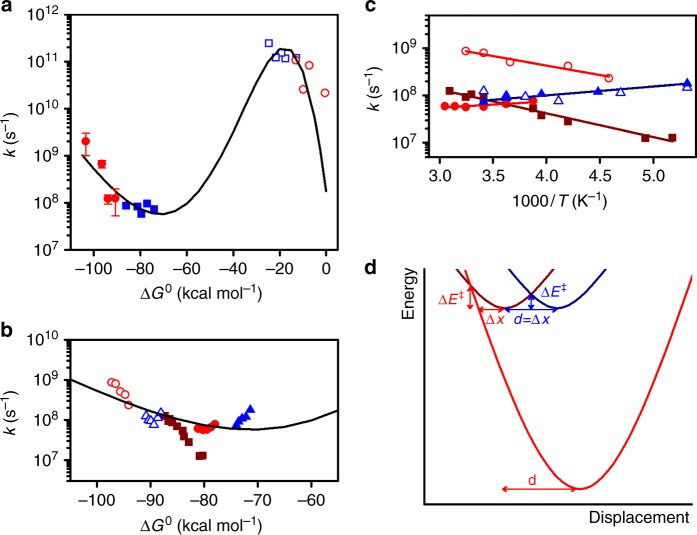
Driving force and temperature dependencies of electron transfer rates. **a** Charge separation (open symbols) and charge recombination (closed symbols) in **1** (squares) and **2** (circles) as a function of the driving force at *T* = 293 K. Points with error bar present average value and sample standard deviation of three to six independent measurements. **b** Charge recombination as a function of the driving force for all temperatures: 1/DCM (full triangles), 1/CHF (full circles), 1/NBE (full squares), 2/DCM (open triangles) and 2/CHF (open circles); the driving forces were estimated at each temperature using the dielectric constant at that temperature, interpolated from the available dielectric constants (Supplementary Table S2). **c** Temperature dependence of the charge recombinations; symbols as in **b**. **d** ET coordinate using a single averaged mode approximation illustrating the tunneling barrier width (∆*x*) and height (∆*E*^‡^), and the increase of *d* with ∆*G*^0^ given by ISM

^**1**^**1**^**±**^/CHF, ^**1**^**1**^**±**^/EAC, and ^**1**^**1**^**±**^/DCM have mono-exponential fluorescence decays, but two exponentials were required to fit the decays of ^**1**^**1**^**±**^/NBE and ^**1**^**1**^**±**^/IPE, and of ^**1**^**2**^**±**^ in all solvents (Supplementary Figs. 11-12 and Supplementary Tables 6, 7). The mechanism in Fig. 1 is analogous to the Birks mechanism for excimer-exciplex decays and was solved to extract micro-constants from lifetimes fitted to fluorescence decays using ∆*G*_ST_ = –0.3 kcal mol^–1^ (Supplementary Note 6). Supplementary Table 8 shows that the ^1^*k*_CR_ rate constants obtained are in excellent agreement with the reciprocal of τ_2_ given by target analysis of transient spectra, with the exception of **2**/NBE at 293 K. ^**1**^**2**^±^/NBE has the lowest fluorescence emission (Supplementary Fig. 12) and a weak transient absorption spectrum, both contributing to larger errors in ^1^*k*_CR_. Additionally, the charge separation may not be totally irreversible in view of its small exothermicity. Our best estimate at 293 K is ^1^*k*_CR_ = (2 ± 1)x10^9^ s^–1^ for ^**1**^**2**^±^/NBE at ∆*G*° = –103.4 kcal mol^–1^, which is many orders of magnitude faster than expected from conventional wisdom for an ET deep into the inverted region. The free-energy dependence of ET rates is presented in Fig. 5a.

It is tempting to assign the fast rates (^1^*k*_CR_ > 10^8^ s^–1^) observed for ∆*G*° < –75 kcal mol^–1^ to a change in mechanism. Intersystem crossing, fluorescence or photodecomposition could become increasingly important in the less polar solvents and dominate the decay of ^1^CT. We used flash photolysis to generate ^**3**^**1** upon excitation of **1** at 266 nm in various solvents and compared ^**3**^**1** with the triplet state of dMA under the same conditions. Flash photolysis of **1** in NBE generated a band at 330 nm with a 2.0 ± 0.2 µs lifetime, which is quenched by oxygen, in good agreement with the triplet decay of anisole (τ_T_ = 2.1 ± 0.2 µs, triplet quantum yield Φ_T_ = 0.64)^16^. Assuming Φ_T_ = 0.64 for dMA, we calculate Φ_T_ = 0.14 for **1** in NBE from the ratio of the initial decay intensities of ^**3**^**1** and dMA. Attempts to see ^**3**^**1** in DCM, EAC and CHF were unsuccessful, consistent with |∆*G*_IP_| ≤ *E*_T1_ in more polar solvents. We could not detect ^**3**^**2** in the same experimental conditions. The fluorescence of ^**1**^**2**^**±**^ is a small fraction of the fluorescence of oXy in NBE, proving the minor contribution of the radiative channel. The spectra of **1** and **2** did not change with the laser experiments and exclude photodecomposition.

The temperature dependence of ^1^*k*_CR_ provides further insight into the end of the inverted region at ∆*G*°≈–75 kcal mol^–1^. ET rates in the inverted region are believed to be essentially temperature independent^28–30^, but *E*_a_ < 0 may be observed in intramolecular charge recombination reactions when *ε* increases as the temperature decreases, or when a state favorable for ET is depopulated with an increase in temperature^31^. CR rates in an intramolecular D–A system were shown to exhibit an inverted parabola dependence on 1/*T* and claimed as the “cleanest confirmation” of Marcus energy gap law^32^.

^**1**^**1**^±^/DCM charge-recombination rates decrease as *T* increases affording *E*_a_ = –0.86 ± 0.10 kcal mol^–1^. This negative effective activation is assigned to the increase of *ε* from 9.0 at 293 K to 15.5 at 187 K, with the corresponding decrease in exothermicity from –74.0 to –71.4 kcal mol^–1^ (Supplementary Table 2). Figure 5b plots ^1^*k*_CR_ vs. ∆*G*° at various temperatures to show that part of the temperature dependence may be assigned to the dependence of *ε* on *T*. On the other hand, ^1^*k*_CR_ of ^**1**^**1**^±^/NBE increases when the temperature increases, and affords *E*_a_ = 2.32 ± 0.12 kcal mol^–1^. The change from *E*_a_ < 0 for ∆*G*° > –75 kcal mol^–1^ to *E*_a_ > 0 for ∆*G*° < –80 kcal mol^–1^ was also found from ^**1**^**1**^±^/CHF (*E*_a_ = –0.61 ± 0.20 kcal mol^–1^) to ^**1**^**2**^±^/CHF (*E*_a_ = 1.83 ± 0.25 kcal mol^–1^). This leads to the extraordinary observation that ^1^*k*_CR_ is larger for ^**1**^**2**^±^/CHF than for ^**1**^**1**^±^/CHF (6.7 × 10^8^ s^–1^ vs. 5.8 × 10^7^ s^–1^ at 293 K), i.e., the faster reaction has higher activation energy. Figure 5c presents the Arrhenius plots of these systems. The change from negative to positive effective activation energies takes place at ∆*G*° ≈–75 kcal mol^–1^, where the exothermic rate restrictions predicted by Marcus theory are lifted for these systems.

The ET rates in Fig. 5 challenge the established view of ET reactions: Marcus inverted region ends at –75 kcal mol^–1^, activation energies change from negative effective to positive at this driving force, all moderately polar solvents fit the same free-energy dependence, and the least polar solvents (ethers) do not have the lowest rates of the inverted region. For example, ^1^*k*_CR_ increases from 8.7 × 10^7^ s^–1^ for ^**1**^**1**^±^/NBE (∆*G*^0^ = –86 kcal mol^–1^) to 8.4 × 10^8^ s^–1^ for ^**1**^**2**^±^/NBE (∆*G*^0^ = –103 kcal mol^–1^), and decreases to 1.3 × 10^8^ s^–1^ for ^1^**2**^±^/DCM (∆*G*^0^ = –91 kcal mol^–1^). As shown above, this new behavior cannot be assigned to a change in mechanism.

ET rates are often expressed as the product between electronic (*V*) and Franck-Condon factors (FCWD)

1 $$k = \frac{{2\pi }}{\hbar }\left| {V^2} \right|{\rm{FCWD}}$$

and the temperature independence of very exothermic ETs is properly described when^33^

2 $${\rm{FCWD}} = \frac{1}{\sqrt {4\pi \lambda _{s}k_{\mathrm{B}}T} } \sum\limits_{n = 0}^{\infty} e^{ - S}\frac{S^{n}}{n!}\exp \left[ - \frac{\left( {\Delta G^0 + \lambda _s + nh\nu _{\nu} } \right)^2}{4\lambda _{s}k_{\mathrm{B}}T} \right]$$

Here the high-frequency vibrations are treated as a single vibrational mode (*hν*_v_) with a reduced displacement *S* = *λ*_v_/(*hν*_v_). A vibrational frequency *hν*_v_ = 1500 cm^–1^ is often used to represent aromatic donors and acceptors^11^, although the coupling to very high-frequency modes (e.g., *hν*_v_ = 3000 cm^–1^ C–H vibration) increases FCWD at very high driving forces^34^. The dielectric continuum approximation is frequently used to calculate *λ*_s_, but it is increasingly clear that it overestimates *λ*_s_^35^, probably by a factor of two^36–38^. The calculations in Supplementary Note 7 suggest *λ*_s_ = 6 ± 3 kcal mol^–1^ in our solvents. The fastest rates, at –∆*G*^0^ = *λ*, were observed for ∆*G*^0^≈–20 kcal mol^–1^. This indicates that the major contribution to FCWD originates from high-frequency molecular vibrations. This is further corroborated by the observation that the ET rates of **1** measured in deuterated CHF were indistinguishable from the rates in CHF.

In view of the dominance of the high-frequency modes of donor and acceptor, and of the barrierless rates observed, we interpret ET rates in **1** and **2** as radiationless transitions in large molecules, namely as nuclear tunneling of promoting and accepting modes of effective mass *µ*_DA_^39^

3 $$k = \nu \exp \left[ { - \frac{{\Delta x\sqrt {2\mu _{{\rm{DA}}}\Delta E^\ddagger } }}{\hbar }} \right]$$

where *ν* is the reaction frequency, and ∆*x* and ∆*E*^‡^ are represented in Fig. 5. The nonadiabatic multiphonon formalism leads to an analogous equation when the solvent is neglected and the nuclear distortions are described by an averaged single mode^40^. In fact, Eq. (3) is the WKB solution for nuclear tunneling through a barrier formed by intersecting parabolas.

The reaction frequency *ν* is related to *V*. We calculate *ν*≈2 × 10^11^ s^–1^ from the electronic frequency in the donor and the electron tunneling probability through a square energy barrier *r*_e_ = 5.9 Å width separating donor and acceptor (Supplementary Note 7). In view of the relations between ∆*x*, ∆*G*^0^, *d* and the force constant of the high-frequency vibrational mode (*f*), discussed in the Methods, Eq. (3) only requires the displacement *d* and the effective reduced mass *µ*_DA_ to calculate the rate constants. The Methods show how *µ*_DA_ is related to the reduced masses of the oscillators involved in the radiationless transitions. The averaged single mode approximation for donor and acceptor relates *d* to the square root of the mean squared displacements^39,41,42^ and yields *d*_msd_ = 0.164 Å (Supplementary Note 7). Alternatively, in this work, we estimated *d* using the Intersecting-State Model (ISM).

According to ISM, the total displacement of an averaged single mode with equilibrium bond length *l*_eq_ is^35^

4 $$\begin{array}{l}d_{{\rm{ISM}}} = \frac{{a\prime }}{{2n^\ddagger }}\ln \left[ {\frac{{1 + g}}{{1 - 1/(1 + g)}}} \right]\left( {l_{r,eq} + l_{p,eq}} \right),\\ g = \exp \left( {\sqrt {2n^\ddagger } \Delta G^0/\Lambda } \right)\end{array}$$

where *a*′ = 0.156 is a scaling constant and *n*^‡^ is the averaged bond order. When ∆*G*^0^ = 0, *d*_ISM_ is independent of Λ. Then, averaging the values of the aromatic and dicyanoethene moieties (*n*^‡^ = 1.75, *l*_eq_ = 1.37 Å, *f* = 1.14 × 10^3^ kcal mol^−1^ Å^−2^)^30,43,44^ gives *d*_ISM_ = 0.169 Å. This is in good agreement with *d*_msd_ calculated by GAMESS and yields an intrinsic barrier ∆*G*_0_^‡^ = *λ*/4≈4 kcal mol^–1^. For finite values of Λ, *d*_ISM_ increases with |∆*G*^0^|, i.e., the reorganization energy increases with the driving force. This increase is associated with the disposal of reaction energy in otherwise spectator modes when a large amount of energy must be dissipated. The coupling parameter Λ is the only parameter in Eqs. (3), (4) that is not calculated from molecular properties. Figure 5 shows that Eqs. (3, 4) with parameters typical of aromatic and dicyanoethene modes and Λ = 70 kcal mol^–1^, used for similar systems^43,44^, describe the driving force and temperature dependences of ET rates and account the transition to positive activation energies at the driving force where the new “normal” region begins.

We observed higher ET rates with higher apparent activation energies. This paradox is solved realizing that higher temperatures decrease *ε* and increase charge recombination exothermicities, which increase their reorganization energies. Established ET theories need to be revised to accommodate the increase of *λ* with |∆*G*^0^| and the end of Marcus inverted region. The ability to describe these phenomena with a tunneling model and *d*_ISM_, suggests that exothermicity is increasingly dissipated to accepting very high-frequency modes, not covered by the frontier molecular orbitals. The contribution of these additional modes increases the reorganization energy with the driving force. The coupling of reactive modes to otherwise spectator modes suggests that the lifetimes of high-energy charge-separated states in molecular electronics and photovoltaic cells can be increased, and the efficiencies improved, uncoupling donor and acceptor moieties from spectator modes. The local mode behavior of such modes should decrease charge recombination rates of very exothermic ET.

## Methods

### Materials

Unless otherwise noted, all commercial materials were used without further purification. Solvents for the synthesis were obtained from Sigma-Aldrich. Malononitrile (Aldrich, > 99%) was recrystallized from ether^45^. Pseudosaccharyl chloride was prepared following a described procedure^46^. 3,4-Dimethylanisole (dMA, Aldrich, > 99%), isopropylidenemalononitrile (iPN, TCI Europe, > 98%) were used as received. EAC (Carlo Erba, > 99%), chloroform (CHF, Carlo Erba ≥ 99.9%) and dichloromethane (DCM, Sigma Aldrich, ≥ 99.9%) were used without additional purification. IPE (Merck, 98%) was dried over CaSO_4_ and passed by activated alumina column. NBE (NBE, Acros Organics, ≥ 99.9) passed through an activated silica column, and then distilled over CaCl_2_.

### Synthesis of the estrone derivative 1

The estrone derivative **1** was prepared according the following scheme:
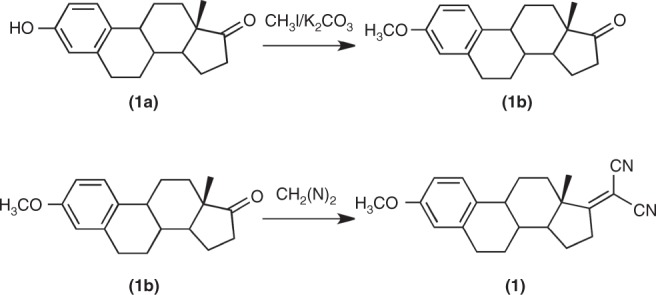


Synthesis of the estrone derivative **1b** (**3-methoxy-1,3,5 (10)-estratrien-17-one**). In a pressure tube 1.0 g (3.7 mmol) of estrone (3-hidroxy-1,3,5 (10)-estratrien-17-one) (**1a**), 1.2 g (3.6 mmol) of potassium carbonate and 2.0 mL of methyl iodide (16 mmol) were added to 50 mL of acetone. The mixture was placed in an oil bath at 60 °C for 24 h. The formation of the methyl ether was monitored by NMR. Incomplete methylation led to the addition of more methyl iodide and the reaction was continued. The precipitate formed was filtered and the liquid evaporated at reduced pressure. The solid residue was recrystallized in dichloromethane/methanol originating 0.81 g (*η* = 78 %) of the estrone derivative **1b** (m.p. = 168–169.5 °C).

Synthesis of the estrone derivative **1 (3-methoxy-1,3,5 (10)-estratrien-17-yliden)malononitrile**). The introduction of the dicyano group was carried out using described procedures^4,47^. In a round flask 400 mg of **1b** (1.4 mmol), 590 mg of ammonium acetate, 1.52 mL of acetic acid and 309 mg (4.68 mmol) of malononitrile were added to 30 mL of toluene. The solution was refluxed overnight in a Dean-Stark apparatus, under nitrogen. The solution was treated with a saturated solution of NaHCO_3_ (50 mL), washed with water, dried over Na_2_SO_4_ and the solvent evaporated under reduced pressure. The residue was subject to column chromatography (silica, DCM and then EAC). The product was recrystallized in ethanol originating 273 mg (*η* = 56 %) of **1** (m.p. = 188–189 °C; 190 °C)^48^.

### Synthesis of the estrone derivative 2

The estrone derivative **2** was prepared according the following scheme
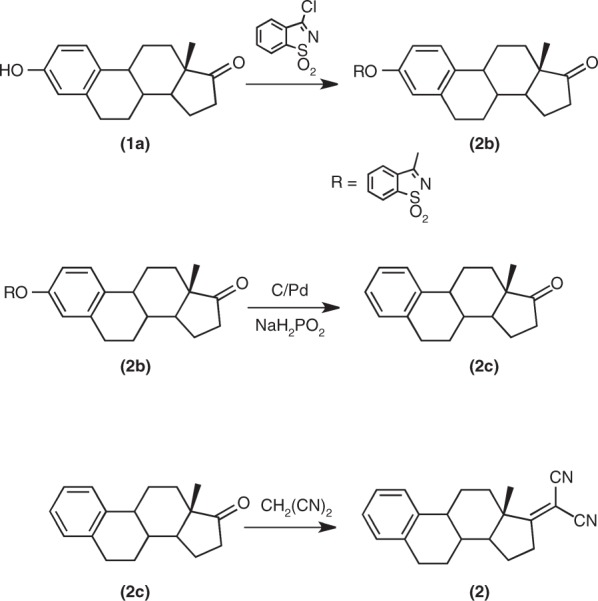


The estrone derivative **2b** (**3-[1,2-benzisothiazole-1,1-dioxide]-1,3,5 (10)-estratrien-17-one**) was prepared following a described procedure^49^. In a round bottom flask 1.2 g of **1a** (4.4 mmol), 0.7 mL of triethylamine and 1.0 g (4.9 mmol) of pseudosaccharyl chloride were added to 100 mL of toluene and refluxed for 2 h under N_2_. The hot solution was filtered and the liquid left to cool to room temperature. The precipitate **2b** was filtered and dried. The material was chromatographed (silica-gel DCM/ethyl ether 3:1) to yield 0.70 g (*η* = 36 %) of **2b** (m.p. = 261–263 °C (decomp.).

The estrone derivative **2c** (**1,3,5 (10)-estratrien-17-one**) was prepared following a described procedure^49^. In a round bottom flask 0.45 g (0.10 mmol) of **2b** and 1.0 g of C/Pd (10 %) were added to 100 mL of benzene and heated to reflux. A solution of 2.6 g (30 mmol) of sodium hypophosphite in 50 mL of water was added stepwise and the mixture was stirred under reflux during 4 h. After cooling to room temperature the catalyst was filtered. The solution was extracted with ethyl ether, washed with water and dried with anhydrous sodium sulfate. Solvent evaporation originates a solid material that was chromatographed (silica-gel, DCM/ethyl ether 10:1). The first fraction was collected and corresponded to 0.20 g (0.078 mmol) of **2c** (*η* = 78 %). m.p. = 134–135 °C (134–135 °C)^49^.

The estrone derivative **2** (**1,3,5 (10)-estratrien-17-yliden)malononitrile**) was prepared following a described procedure^4,47^. In a round flask 0.25 g of **2c** (0,098 mmol) 0.37 g (4.8 mmol) of ammonium acetate, 1.0 mL of acetic acid and 0.19 g (2.9 mmol) of malononitrile were added to 30 mL of toluene. The solution was refluxed overnight in a Dean-Stark apparatus, under nitrogen. The solution is treated with a saturated solution of NaHCO_3_ (50 mL), washed with water, dried over Na_2_SO_4_ and the solvent evaporated under reduced pressure. The residue is chromatographed (silica- DCM and then EAC). The product is recrystallized in ethanol originating 0.19 g (*η* = 65 %) of **2** (m.p. = 200–202 °C).

The characterization of estrone derivatives **1** and **2** is described in Supplementary Note 1 and Supplementary Figures 1-5.

### Conformational searches and DFT calculations

A first search of conformers was made with OpenBabel using the confab model, and Marvin 17.2.27 2017 ChemAxon using the conformer plugin. We set a threshold of 50 kcal mol^–1^ in OpenBabel and did not find any conformers. In Marvin we used the strict optimization limit and the hyperfine post-processing step. All the conformers found differ by minor orientations of the methoxy group linked to the donor moiety, which are irrelevant for the electronic coupling between donor and acceptor moieties. The Supplementary Movie 1 offers various perspectives of the overlaid conformers and shows their similarity. It is very much likely that they collapse into one single conformer with more accurate energy minimization procedures.

Conformational search with GAMESS^50^ using the B3LYPV1R hybrid functional^51–53^ and the 6–31 G(d) Pople basis set for all atoms^54^, revealed the presence of only one low energy conformer in the ground state of **1**. All singlet states were described using RHF formalism and the triplet state used UHF formalism. There was no relevant spin contamination on the UHF calculations, < *S*^2^ > = 2.022. It is clear that the molecules are not conformationally flexible in the region covering donor, spacer and acceptor, and the electronic coupling is unlikely to change with the temperature.

Geometries and conformations are presented in Supplementary Note 2, Supplementary Table 1 and Supplementary Movie 1).

### Time-resolved measurements

Fluorescence decays in the time window between 250 ps to 20 ns were measured using a home-built TCSPC apparatus^55^ with a Horiba-JI-IBH NanoLED (*λ*_ex_ = 282 nm) as excitation source. Fluorescence decays times with shorter time resolution were investigated using a picosecond time correlated single photon counting apparatus (TCSPC, *λ*_ex_ = 272–273 nm)^56^. The excitation source consisted of a picosecond Spectra Physics mode-lock Tsunami laser (Ti:sapphire) model 3950 (repetition rate of about 82 MHz, tuning range 700–1000 nm), pumped by a Millennia Pro-10s, frequency-doubled continuous wave (CW), diode-pumped, solid-state laser (*λ*_em_ = 532 nm). A harmonic generator model GWU-23PS (Spectra-Physics) was used to produce a third harmonic from the Ti:sapphire laser exciting beam frequency output. Deconvolution of the fluorescence decay curves was performed using the modulating function method, as implemented by G. Striker in the SAND program^57^. All the fluorescence decays were measured in 5 or 10 mm quartz cuvettes in the presence of oxygen. Temperatures control was achieved using a cryostat Optistat DN2 (188–308 K) or cuvette holder Flash 300 (253–328 K).

Flash photolysis employed excitation at 266 nm from the fourth harmonic of Nd:YAG laser (Spectra Physics) and the Applied Photophysics LKS.60 laser-flash-photolysis spectrometer^58^. Samples for flash photolysis were measured in the presence of oxygen, and in inert atmosphere (samples were bubbled with N_2_ for 30 min prior to every experiment).

The experimental setup for the ultrafast spectroscopic and kinetics measurements consisted of a broadband (340–1600 nm) HELIOS pump-probe femtosecond transient absorption spectrometer from Ultrafast Systems, equipped with an amplified femtosecond Spectra-Physics Solstice-100F laser (displaying a pulse width of 128 fs and 1 kHz repetition rate), coupled with a Spectra-Physics TOPAS Prime F optical parametric amplifier (195–22 000 nm) for pump pulse generation. Samples of dMA, **1** and **2** were excited with 283, 287, or 273 nm laser pulses at pulse energies of 3, 1, or 1.5 µJ respectively. The probe light in the UV range was generated by passing a small portion of the 795 nm light from the Solstice-100F laser through a computerized optical delay (with a time window of up to 8 ns) and then focusing in a vertical translating CaF_2_ crystal to generate a white-light continuum (340–650 nm). All the measurements were made in 1 or 2 mm quartz cuvettes, with absorptions in the range 0.2–0.5 at the pump excitation wavelength. To avoid photodegradation, the sample was kept in movement using a motorized translating sample holder or stirred.

Steady-state spectroscopic measurements are described in Supplementary Note 3.

### Analysis of kinetic data

The transient absorption data were analyzed using the Surface Xplorer PRO program from Ultrafast Systems and Glotaran for global and target analysis^59^. The results from several scans of freshly prepared samples were averaged. Each scan collected around 1000 time points at 310 different wavelengths. Global and target analysis simultaneously analyzed at least 100 wavelengths. A strong nonresonant signal with a relaxation time below 1 ps was observed in all samples and assigned to the solvent and cuvette. In order to eliminate this signal, for each experimental condition employed to study the samples, an experiment was performed just with the solvent in the cuvette. The normalized solvent response was subtracted from sample data point measured under exactly the same conditions. Transient spectra were also corrected for the dispersion of the probe light resulting from propagation through the crystal and sample (chirp correction).

A sequential kinetic scheme with species of increasing lifetimes was used to fit transient spectra collected for each sample, resulting in Evolution-Associated Spectra (EAS). The number of EAS (2 or 3 after the hot state) required to fit the spectra was estimated by inspection of the residuals. The EAS correspond to true SAS when the initially prepared Franck-Condon state of dMA decays to the relaxed singlet state and then to the triplet state (peak at 310 nm and shoulder at 380 nm), or when the relaxed singlet state is quenched by iPN and leads to the aromatic cation (absorption band ≈460 nm). The decays of **1** and of **2** follow the mechanism of Fig. 1, schematically represented in Eq. (5). When the triplet energy is close or higher than that of the charge-transfer (CT) species, which is the case for **1** in DCM, EAC and CHF, the decays can be fit with a sequential kinetic scheme with two species (in addition to the hot state), Eq. (5A), and the EAS are the true SAS. In the other cases, we attempted to use Target Analysis with the mechanism of Eq. (5B), with branching and equilibrium, which yields SAS. For **1** in NBE and IPE and for **2** in CHF the Target Analysis with 3 species (in addition to the hot state) gives all positive spectra, which are true SAS. For **2** in DCM, EAC, and NBE the spectra were very weak for a reliable Target Analysis, and Global Analysis with sequential kinetic scheme involving three species (in addition to the hot state) was performed. This revealed the need for an additional lifetime and the difficulty to associated it with a spectrum. In this case the EAS are not the true SAS
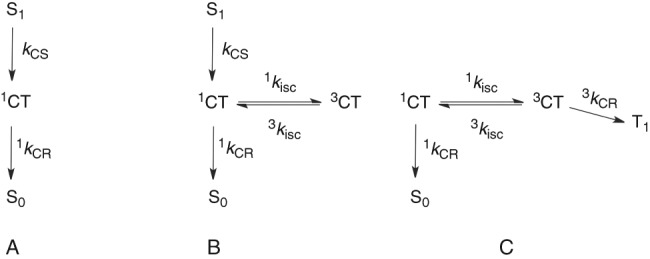


Femtosecond transient absorption (time resolution between 500 fs and 10 ns) allowed us to measure the formation and decay of the ^1^CT state but the locally excited triplet is formed and decays outside this time window. This is the reason why T_1_ is not considered in Eqs. (5A) and (5B) used to obtain the EAS and SAS, respectively. Single photon counting (SPC, two excitation sources were used, a nanoLED and a laser with instrumental responses of 1 ns and 22 ps, respectively) could not be used to see the initial charge separation. Laser flash photolysis (instrumental response of 20 ns) allowed for the direct observation of T_1_.

SPC data were interpreted with an adaptation of the Birks excimer mechanism presented in Eq. (5C). The difference is that in the Birks mechanism two monomers have to diffuse to yield the excimer and this is a bimolecular reaction, whereas in our mechanism intersystem crossing between singlet and triplet states of the charge transfer (CT) species are first-order reactions. The important consequence of this difference is that the rate of the decay of the monomer in the Birks mechanism (corresponding to ^1^*k*_CR_ in our mechanism) can be obtained at high dilution of the monomer, when it becomes the only relevant decay of the monomer (corresponding to ^1^CT in our mechanism), whereas in our mechanism there is no independent experimental measurement to obtain ^1^*k*_CR_. As shown in Supplementary Note 6, this can be circumvented with a reasonable estimate of the ratio between ^1^*k*_isc_ and ^3^*k*_isc_.

The charge recombination rates independently measured by femtosecond transient absorption and single photon counting are in very good agreement. Figure 5 present the rates from single photon counting because they are better accommodated in the time window of this technique.

### Electron transfer model

When *λ*_s_ → 0, the dominant factor of the low temperature limit for a symmetrical radiationless transfer between two electronic states is^40^

6 $$k = \frac{2\pi}{\hbar }\left| {V^2} \right|\frac{1}{{\hbar \omega_s}}\exp \left[ { - \frac{{d\sqrt {2\mu_{\mathrm{DA}}\Delta E^{\ddagger}}}}{\hbar }} \right]$$

which shows that the nuclear Franck-Condon factor for symmetrical reactions and for exoergic processes can be recast in a form which is practically identical with the Gamov formula. For exoergic processes, the displacement *d* between the minima of the oscillators must be replaced by the barrier width ∆*x*, which is the horizontal distance between the turning points of vibration of the oscillator in the initial and final states of a radiationless transition,

7 $$\Delta x = d - \sqrt {\frac{{2\left| {\Delta E} \right|}}{f}}$$

where the harmonic force constant of the vibrational mode is given by its angular frequency of oscillation, $\omega _\nu = \sqrt {f/\mu }$, knowing the oscillator reduced mass *µ*. For example, the asymmetric CC stretching mode of benzene is observed at 1309 cm^–1^ and can be reproduced with a harmonic force field using a CC stretching mode with a force constant *f*_CC_ ≈ 1000 kcal mol^–1^ Å^–2^ ^60^. This vibration of the benzene ring is also described as the Kekulé mode and corresponds to three CC oscillators being simultaneously displaced from their equilibrium positions in the transfer of benzene from the initial to the final state. Hence, in a first approximation, the effective reduced mass in Eq. (3) should be *µ*_benzene_ = 3*µ*_CC_. However, Eq. (3) was derived for radiationless transitions within a given molecule^39^, while in ET two molecules are involved (or two independent moieties is the same molecule). Within the approximations used to derive Eq. (3), the identical and similarly displaced oscillators involved in the transitions in these moieties have the same frequencies and reduced masses. Hence, the barriers ∆*E*^‡^ are the same for all the oscillators and the total reduced mass for the hypothetical case of two benzene molecules exchanging an electron is *µ*_DA_ = [(*µ*_benzene_)^1/2^ + (*µ*_benzene_)^1/2^]^2^. For the case of molecules **1** and **2**, one of the moieties can be approximated as the benzene ring and the other as dicyanoethylene. We have shown before that the average of the force constants of the relevant oscillators gives *f*_CC_ ≈ 1.15 × 10^3^ kcal mol^–1^ Å^–2^, and that the effective reduced masses are *µ*_benzene_ = 3*µ*_CC_, *µ*_dicyanoethylene_ = *µ*_CC_ + 2*µ*_CN_^30,43,44^. In general the effective reduced mass of the donor-acceptor system is

8 $$\mu _{{\rm{DA}}} = \left( {\sqrt {\mu _{{\rm{donor}}}} + \sqrt {\mu _{{\rm{acceptor}}}} } \right)^2$$

The nuclear tunneling rate constants calculated with Eq. (3) employed ∆*x* calculated with Eqs. (7) and (4), effective reduced masses calculated with Eq. (8) and ν≈2 × 10^11^ s^–1^. The tunneling rates are larger than thermal activation rates calculated over the same energy barrier

9 $$k_{{\rm{th}}} = \nu \exp \left( { - \frac{{\Delta E^\ddagger }}{{{\mathrm{RT}}}}} \right)$$

for ∆*G*^0^ < –20 kcal mol^–1^. However, thermal activation dominates the rates in the normal region. Figure 5 combines the thermal activates rates in the normal region with the tunneling rates elsewhere.

In summary, ISM calculations employed the following set of parameters: *a*′ = 0.156, *n*^‡^ = 1.75, *l*_eq_ = 1.37 Å, *f* = 1.14 × 10^3^ kcal mol^–1^ Å^–2^, Λ = 70 kcal mol^–1^, *µ*_donor_ = 19 amu and *µ*_acceptor_ = 18 amu. These parameters are entirely consistent with those employed in our earlier applications of ISM to ET reactions^35,44^.

### Data availability

The data that support the findings of this study are available from the corresponding author upon reasonable request. Correspondence and requests for materials should be addressed to Prof. Luis Arnaut (lgarnaut@ci.uc.pt).

## Electronic supplementary material

Supplementary Information

Description of Additional Supplementary Files

Supplementary Movie 1

## References

[1] Marcus RA (1956). On the theory of oxidation-reduction reactions involving electron transfer. I. J. Chem. Phys..

[2] Marcus RA (1960). Theory of oxidation-reduction reactions involving electron transfer. Part 4. A statistical-mechanical basis for treating contributions from solvent, ligands and inert salt. Faraday Discuss. Chem. Soc..

[3] Calcaterra LT, Closs GL, Miller JR (1983). Fast intramolecular electron transfer in radical ions over long distances across rigid saturated hydrocarbon spacers. J. Am. Chem. Soc..

[4] Oevering H (1987). Long-range photoinduced through-bond electron transfer and radiative recombination via rigid nonconjugated bridges: Distance and solvent dependence. J. Am. Chem. Soc..

[5] Ihly R (2016). Tuning the driving force for exciton dissociation in single-walled carbon nanotube heterojunctions. Nat. Chem..

[6] Zhang X (2016). Molecular engineering of potent sensitizers for very efficient light harvesting in thin-film solid-state dye-sensitized solar cells. J. Am. Chem. Soc..

[7] Chang W (2015). Spin-dependent charge transfer state design rules in organic photovoltaics. Nat. Commun..

[8] Uoyama H, Goushi K, Shizu K, Nomura H, Adachi C (2012). Highly efficient organic light-emitting diodes from delayed fluorescence. Nature.

[9] Berardi S (2012). Photocatalytic water oxidation: tuning light-induced electron transfer by molecular Co4O4 cores. J. Am. Chem. Soc..

[10] Schubert C, Margraf JT, Clark T, Guldi DM (2015). Molecular wires – impact of π-conjugation and implementation of molecular bottlenecks. Chem. Soc. Rev..

[11] Closs GL, Miller JR (1988). Intramolecular long-distance electron transfer in organic molecules. Science.

[12] Paddon-Row MN (1994). Investigating long-range electron-transfer processes with rigid, covalently linked donor–(norbornylogous bridge)–acceptor systems. Acc. Chem. Res..

[13] Sukegawa J (2014). Electron transfer through rigid organic molecular wires enhanced by electronic and electron–vibration coupling. Nat. Chem..

[14] Freeman DME (2017). Synthesis and exciton dynamics of donor-orthogonal acceptor conjugated polymers: Reducing the singlet–triplet energy gap. J. Am. Chem. Soc..

[15] Pasman P, Mes GF, Koper NW, Verhoeven JW (1985). Solvent effects on photoinduced electron transfer in rigid, bichromophoric systems. J. Am. Chem. Soc..

[16] Montalti, M., Credi, A., Prodi, L. & Gandolfi, M. T. *Handbook of Photochemistry*. 3rd edn, (CRC Press, 2006).

[17] Weller A (1982). Photoinduced ET in solution: exciplex and RIP formation enthalpies and their solvent dependence. Z. Phys. Chem..

[18] Merkel PB, Luo P, Dinnocenzo JP, Farid S (2009). Accurate oxidation potentials of benzene and biphenyl derivatives via electron-transfer equilibria and transient kinetics. J. Org. Chem..

[19] Bixon M, Jortner J, Verhoeven JW (1994). Lifetimes for radiative charge recombinations in donor-acceptor molecules. J. Am. Chem. Soc..

[20] Gould IR, Young RH, Mueller LJ, Albrecht AC, Farid S (1994). Electronic structures of exciplexes and excited charge-transfer complexes. J. Am. Chem. Soc..

[21] Takamuku S, Komitsu S, Toki S (1989). Radical cations of anisole derivatives. Novel complex formation. Int. J. Radiat. Appl. Instrum. C.

[22] Shukla D, Liu G, Dinnocenzo JP, Farid S (2003). Controlling parameters for radical cation fragmentation reactions: Origin of the intrinsic barrier. Can. J. Chem..

[23] Weller A, Staerk H, Treichel R (1984). Magnetic-field effects on geminate radical-pair recombination. Faraday Discuss. Chem. Soc..

[24] Paddon-Row MN, Shephard MJ (2002). A time-dependent density functional study of the singlet-triplet energy gap in charge-separated states of rigid bichromophoric molecules. J. Phys. Chem. A.

[25] Okada T (1981). Ultrafast intersystems crossing in some intramolecular heteroexcimers. J. Phys. Chem..

[26] van Willingen H, Jones G, Farahat MS (1996). Time-resolved EPR study of photoexcited triplet-state formation in electron-donor-substituted acridinium ions. J. Phys. Chem..

[27] Dance ZEX (2008). Intersystem crossing mediated by photoinduced intramolecular charge transfer: Julolidine-anthracene molecules with perpendicular π systems. J. Phys. Chem. A.

[28] Liang N, Miller JR, Closs GL (1990). Temperature-independent long-range electron transfer reactions in the Marcus inverted region. J. Am. Chem. Soc..

[29] Kroon J (1993). Temperature effects on intramolecular electron transfer kinetics under “normal”, “inverted” and “mearly optimal” conditions. J. Phys. Chem..

[30] Serpa C, Gomes PJS, Arnaut LG, Formosinho SJ, Seixas de Melo J (2006). Temperature dependence of ultra-exothermic charge recombinations. Chemphyschem.

[31] Scott AM, Wasielewski MR (2011). Temperature dependence of spin-selective charge transfer pathways in donor-bridge-acceptor molecules with oligomeric fluorenone and p-phenylethynylene bridges. J. Am. Chem. Soc..

[32] Waskasi MM (2016). Marcus bell-shaped electron transfer kinetics observed in an Arrhenius plot. J. Am. Chem. Soc..

[33] Bixon M, Jortner J (1991). Non-arrenius temperature dependence of electron-transfer rates. J. Phys. Chem..

[34] Bixon M, Jortner J, Cortes J, Heitele H, Michel-Beyerle ME (1994). Energy gap law for nonradiative and radiate charge transfer in isolated and solvated supermolecules. J. Phys. Chem..

[35] Formosinho SJ, Arnaut LG, Fausto R (1998). A critical assessment of classical and semi-classical models for electron transfer reactions in solution. Prog. React. Kinet..

[36] Newton MD, Basilevsky MV, Rostov IV (1998). A frequency-resolved cavity model (FRCM) for treating equilibrium and non-equilibrium solvation energies. 2: Evaluation of solvent reorganization energies. Chem. Phys..

[37] Ren HS, Ming MJ, Ma JY, Li XY (2013). Theoretical calculation of reorganization energy for electron self-exchange reaction by constrained density functional theory and constrained equilibrium thermodynamics. J. Phys. Chem. A.

[38] Li XY (2015). An overview on continuum models for nonequilibrium solvation: popular theories and new challenge. Int. J. Quantum Chem..

[39] Formosinho SJ (1974). Quantum mechanical tunnelling in the radiationless transitions of large molecules. J. Chem. Soc. Faraday Trans. 2.

[40] Jortner J, Ulstrup J (1979). Tunnelling in low-temperature atom-transfer processes. Chem. Phys. Lett..

[41] McCoy EF, Ross IG (1962). Electronic states of aromatic hydrocarbons: the Franck-Condon principle and geometries in excited states. Aust. J. Chem..

[42] Van Duyne RP, Fischer SF (1974). A nonadiabatic description of electron transfer reactions involving large free energy changes. Chem. Phys..

[43] Serpa C (2006). Electron transfers in supercritical CO_2_. Ultra-exothermic charge recombinations at the end of the ‘inverted region’. Chem. Eur. J..

[44] Mentel KK, Nunes RMD, Serpa C, Arnaut LG (2015). Dynamics of radical ion pairs following photoinduced electron transfer in solvents with low and intermediate polarities. J. Phys. Chem. B.

[45] Ferris JP, Sanchez RA, Mancusi RW (1968). Aminomalononitrile p- toluenesulfonate. Org. Synth..

[46] Meadow JR, Cavagnol JC (1952). Use of saccharin derivatives for the identification of mercaptans. J. Org. Chem..

[47] Lawson JM, Craig DC, Oliver AM, Paddon-Row MN (1995). Synthesis and structural characterization of a novel pair of rigid diastereomeric triads. Tetrahedron.

[48] Annen K, Hofmeister H, Laurent H, Seeger A, Wiechert T (1978). Eine neuartige einfache Dreiring-Synthese. Chem. Ber..

[49] Brigas AF, Johnstone RAW (1990). Metal-assisted reactions. Part 20. Catalytic transfer hydrogenolysis of phenolic CO bonds. Tetrahedron Lett..

[50] Schmidt MW (1993). General atomic and molecular electronic structure system. J. Comput. Chem..

[51] Becke AD (1993). Density-functional thermochemistry. III. The role of exact exchange. J. Chem. Phys..

[52] Stephens PJ, Devlin FJ, Chabalowski CF, Frisch MJ (1995). Ab initio calculation of vibrational absorption and circular dichroism spectra using density functional force fields: a comparison of local, nonlocal, and hybrid density functionals. J. Phys. Chem..

[53] Hertwig RH, Koch W (1997). On the parameterization of the local correlation functional. What is Becke-3-LYP?. Chem. Phys. Lett..

[54] Ditchfield R, Hehre WJ, Pople JA (1971). Self‐consistent molecular-orbital methods. IX. An extended Gaussian-type basis for molecular-orbital studies of organic molecules. J. Chem. Phys..

[55] Pina J (2006). Photophysical studies of alpha,omega-dicyano-oligothiophenes NC(C4H2S)nCN (n=1-6). J. Phys. Chem. B.

[56] Pina J (2009). Alternating binaphthyl-thiophene copolymers: synthesis, spectroscopy, and photophysics and their relevance to the question of energy migration versus conformational relaxation. Macromolecules.

[57] Stricker G, Subramaniam V, Seidel CAM, Volkmer A (1999). Photochromicity and fluorescence lifetimes of Green Fluorescent Protein. J. Phys. Chem. B.

[58] Serpa C, Arnaut LG (2000). Does molecular size matter in photoinduced electron transfer reactions?. J. Phys. Chem. A.

[59] van Stokkum IHM, Larsen DS, van Grondelle R (2004). Global and target analysis of time-resolved spectra. Biochim. Biophys. Acta.

[60] Pulay P, Fogarasi G, Boggs JE (1981). Force field, dipole moment and vibronic constants of benzene from a combination of experimental and ab initio quantum chemical information. J. Chem. Phys..

