# Supplementary material for: Higher activation barriers can lift exothermic rate restrictions in electron transfer and enable faster reactions
Source: Nat Commun. 2018 Jul 25;9:2903. doi: 10.1038/s41467-018-05267-5 (PMC6060101; doi:10.1038/s41467-018-05267-5)
Supplement: Supplementary file 1 — Supplementary Information [file 41467_2018_5267_MOESM1_ESM.pdf]

Supplementary Information

Higher Activation Barriers Lifting Exothermic Rate Restrictions in Electron Transfers and Enable Faster Reactions

Kamila K. Mentel,[1] Arménio Serra,[2] Paulo Abreu,[1] Luis G. Arnaut[1,*]

[1] Chemistry Department, University of Coimbra, Portugal

[2] Chemical Engineering Department, University of Coimbra, Portugal

Supplementary Note 1. Materials, Synthesis and Characterization 2
Supplementary Figure 1 4
Supplementary Figure 2 5
Supplementary Figure 3 6
Supplementary Figure 4 7
Supplementary Figure 5 8

Supplementary Note 2. Conformational searches and DFT calculations 9
Supplementary Table 1 9

Supplementary Note 3. Experimental procedures 10
Supplementary Figure 6 10
Supplementary Figure 7 10
Supplementary Figure 8 11
Supplementary Figure 9 11

Supplementary Note 4. Reaction energy calculations 12
Supplementary Table 2 12
Supplementary Table 3 13
Supplementary Table 4 14

Supplementary Note 5. Intersystems crossing to the triplet 15

Supplementary Note 6. Analysis of transient absorption and single-photon counting data 18
Supplementary Figure S10 18
Supplementary Table 5 19
Supplementary Table 6 20
Supplementary Figure 11 22
Supplementary Figure 12 23
Supplementary Table 7 24
Supplementary Table 8 25
Supplementary Table 9 26
Supplementary Table 10 27
Supplementary Figure 13 28

Supplementary Note 7. Electron transfer rate constants 29

Supplementary Note 8. Transient absorption spectra 33

Supplementary References 44

Supplementary Note 1

Materials, Synthesis and Characterization

Synthesis of the estrone derivative 1:

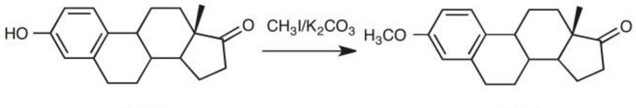

(1a) (1b)

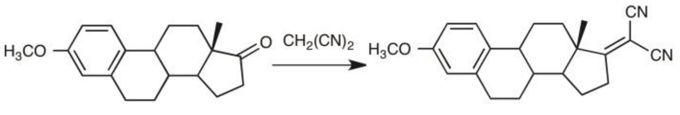

(1b) (1)

3-Methoxy- 1,3,5 (10)-estratrien-17-one (**1b**): IR (KBr) 3448, 2971, 2948, 2914, 1737, 1610, 1502, 1454, 1316, 1286, 1245, 1036, 846, 824 cm^{-1}; ^1H NMR (400 MHz, $CDCl_3$) δ 0.84 (s, 3H), 1.34 – 1.58 (m, 6H), 1.86 – 2.12 (m, 4H), 2.15 – 2.21 (m, 1H), 2.30 – 2.35 (m, 1H), 2.43 (dd, 1H, J = 8.4 Hz, J = 18.8 Hz), 2.80 – 2.85 (m, 2H), 3.71 (s, 3H), 6.58 (d, 1H, J = 2.8 Hz), 6.65 (dd, 1H, J = 2.8 Hz, J = 8.4 Hz), 7.13 (d, 1H, J = 8.4 Hz); ^{13}C NMR (100 MHz, CDCl3) δ 13.9, 21.6, 25.9, 26.6, 29.9, 31.6, 35.9, 38.4, 44.0, 48.0, 50.4, 55.2, 111.6, 113.9, 126.4, 132.0, 137.8, 157.6, 220.9; TOF MS (EI+) *m/z*: Calculated for $C_{19}H_{24}O_2$ [M+] 284.18, found [M+] 284.18.

3-Methoxy-1,3,5 (10)-estratrien-17-yliden)malononitrile (**1**): IR (KBr) 3433, 2948, 2926, 2874, 2837, 2233, 1606, 1573, 1495, 1454, 1282, 1252, 1036, 887 cm^{-1}; ^1H NMR (400 MHz, $CDCl_3$) δ 1.07 (s, 3H), 1.46 –1.62 (m, 5H), 1.71-1.78 (m, 1H), 1.93 –2.05 (m, 2H), 2.26 – 2.31 (m, 1H), 2.48 (m, 1H), 2.46-2.50 (m, 1H), 2.68-2.71 (m, 1H), 2.75 – 3.02 (m, 4H), 3.78 (s, 3H), 6.65 (s, 1H), 6.74 (dd, 1H, J = 2.8 Hz, J = 8.8 Hz), 7.19 (d, 1H, J = 8.8 Hz); ^{13}C NMR (100 MHz, $CDCl_3$) δ 16.6, 23.3, 26.3, 27.4, 29.5, 33.9, 34.7, 38.2, 43.2, 49.4, 54.1, 55.2, 79.7, 111.2, 111.7, 112.3, 113.9, 126.3, 131.3, 137.5, 157.8, 196.1; TOF MS (EI+) *m/z*: Calculated for $C_{22}H_{24}N_2O$ [M+] 332.44, found 332.19. Anal. Calcd. for $C_{22}H_{24}N_2O$: C, 79.48; H, 7.28; N, 8.43. Found: C, 79.08; H, 7.15; N, 8.31.

Synthesis of the estrone derivative 2:

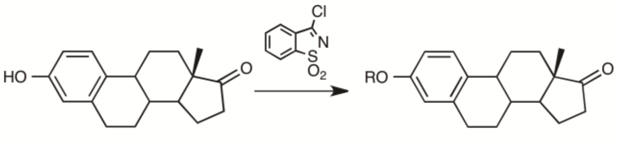

(1a) (2b)

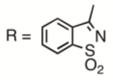

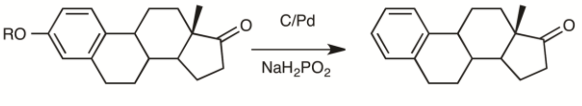

(2b) (2c)

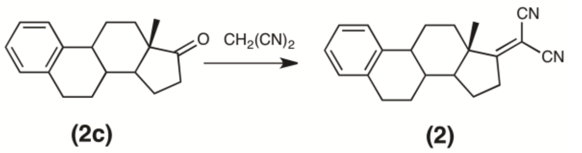

(2c) (2)

3-[1,2-Benzisothiazole-1,1-dioxide]-1,3,5 (10)-estratrien-17-one (**2b**): IR (KBr) 3459, 3101, 2930, 2866, 1737, 1621, 1558, 1491, 1387, 1338, 1312, 1178, 1155, 1051, 898, 772 cm^{-1}; ^1H NMR (400 MHz, $CDCl_3$) δ 0.93 (s, 3H), 1.43 – 1.70 (m, 6H), 1.97 – 2.20 (m, 4H), 2.29 - 2.36 (m, 1H), 2.41 – 2.46 (m, 1H), 2.52 (dd, 1H, J = 8.6 Hz, J = 19.0 Hz), 2.94 – 2.98 (m, 2H), 7.09 – 7.13 (m, 2H), 7.37 – 7.31 (d, 1H, J = 8.4 Hz), 7.81 (dddd, 2H, J = 1.2 Hz, J = 7.6 Hz, J = 14.8 Hz, J = 16 Hz), 7.87 (d, 1H, J = 7.2 Hz); ^{13}C NMR (100 MHz, $CDCl_3$) δ 13.8, 21.6, 25.7, 26.5, 29.5, 31.5, 35.9, 37.9, 44.2, 47.9, 50.4, 117.8, 120.6, 122.1, 123.6, 126,8, 126.9, 133.6, 134.4, 138.7, 139.0, 143.7, 149.9, 169.0, 220.6; TOF MS (EI+) *m/z*: Calculated for $C_{25}H_{25}NO_4S$ [M+] 435.15 found 435.15.

1,3,5 (10)-Estratrien-17-one (**2c**): IR (KBr) 3448, 2960, 2933, 2874, 1733, 1487, 1454, 1085, 1051, 1006, 753 cm^{-1}; ^1H NMR (400 MHz, $CDCl_3$) δ 0.92 (s, 3H), 1.41 – 1.68 (m, 6H), 1.96 – 2.20 (m, 4H), 2.30-2.36 (m, 1H), 2.42 – 2.47 (m, 1H), 2.52 (dd, 1H, J = 8.8 Hz, J = 18.8 Hz), 2.92-2.95 (m, 2H), 7.10 – 7.19 (m, 3H), 7.30 – 7.31 (m, 1H); ^{13}C NMR (100 MHz, $CDCl_3$) δ 13.9, 21.6, 25.7, 26.5, 29.4, 31.6, 35.9, 38.1, 44.5, 48.0, 50.6, 125.3, 125.8, 125.8, 129.1, 136.5, 139.7, 220.9; TOF MS (EI+) *m/z*: Calculated for $C_{18}H_{22}O$ [M+] 254.17 found 254.17.

1,3,5 (10)-Estratrien-17-yliden)malononitrile (**2**): IR (KBr) 3433, 2952, 2933, 2863, 1599, 1491, 1454, 1379, 746 cm^{-1}; ^1H NMR (400 MHz, $CDCl_3$) δ 1.08 (s, 3H), 1.43 – 1.54 (m, 2H), 1.59-1.70 (m, 3H), 1.76 (td, 1H, J = 4,0 Hz , J = 12.8 Hz), 1.94 – 2.07 (m, 2H), 2.33 – 2.38 (m, 1H), 2.49-2.55 (m, 1H), 2.69-2.73 (m, 1H), 2.75 – 2.84 (m, 1H), 2.91 – 3.02 (m, 3H), 7.07 – 7.11 (m, 1H), 7.13 – 7.19 (m, 2H), 7.28 – 7.30 (m, 1H); ^{13}C NMR (100 MHz, $CDCl_3$) δ 16.6, 23.3, 26.1, 27.4, 29.2, 34.0, 34.7, 38.0, 43.7, 49.4, 54.3, 79.7, 111.2, 112.3, 125.2, 125.9, 126.1, 129.1, 136.2, 138.9, 196.0; TOF MS (EI+) *m/z*: Calculated for $C_{21}H_{22}N_2$ [M+] 302.18 found 302.18. Anal. Calcd. for $C_{21}H_{22}N_2$: C, 83.40; H, 7.33; N, 9.26. Found: C, 83.02; H, 7.38; N, 8.79.

Nuclear magnetic resonance (NMR) spectra were recorded with Bruker Avance III 400 MHz spectrometer. Chemical shifts are reported in δ ppm using tetramethylsilane as standard in the case of ^1H NMR and chloroform-d (δ 77.00) for ^{13}C NMR. ^1H NMR (400 MHz, $CDCl_3$) and ^{13}C NMR (100 MHz, $CDCl_3$) spectra of compounds are presented in Figures S1-S5. The multiplicities were described by the use of: s (singlet), d (doublet), t (triplet), m (multiplet) and br (broad). Mass spectra were recorded on a VG Autospect M mass spectrometer. Elemental analysis was recorded on Fisons EA 1108-HNSO equipment.

NMR spectra are presented in Supplementary Figures 1-5.

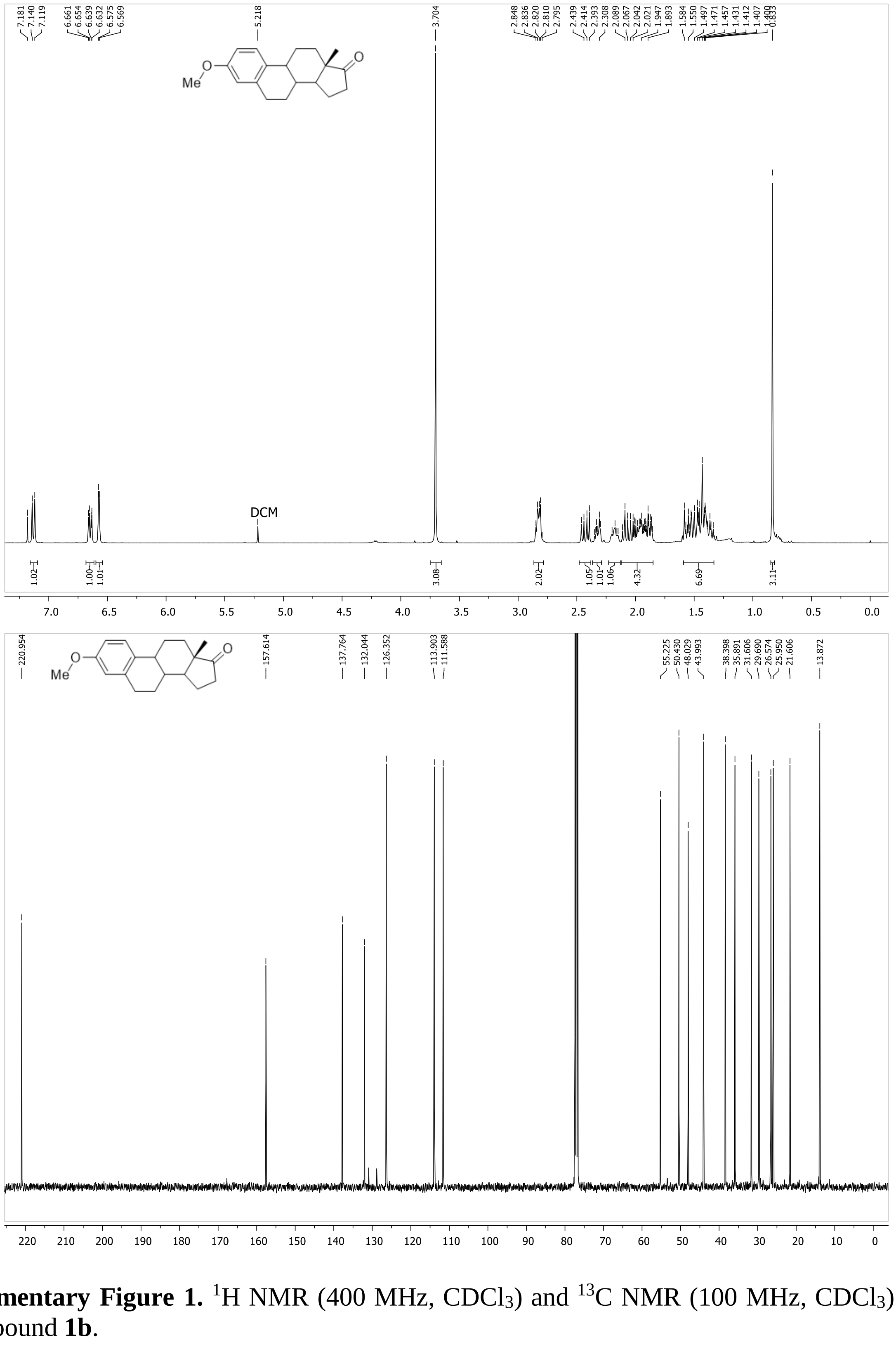

Supplementary Figure 1. ^{1}H NMR (400 MHz, $CDCl_3$) and ^{13}C NMR (100 MHz, $CDCl_3$) spectra of compound **1b**.

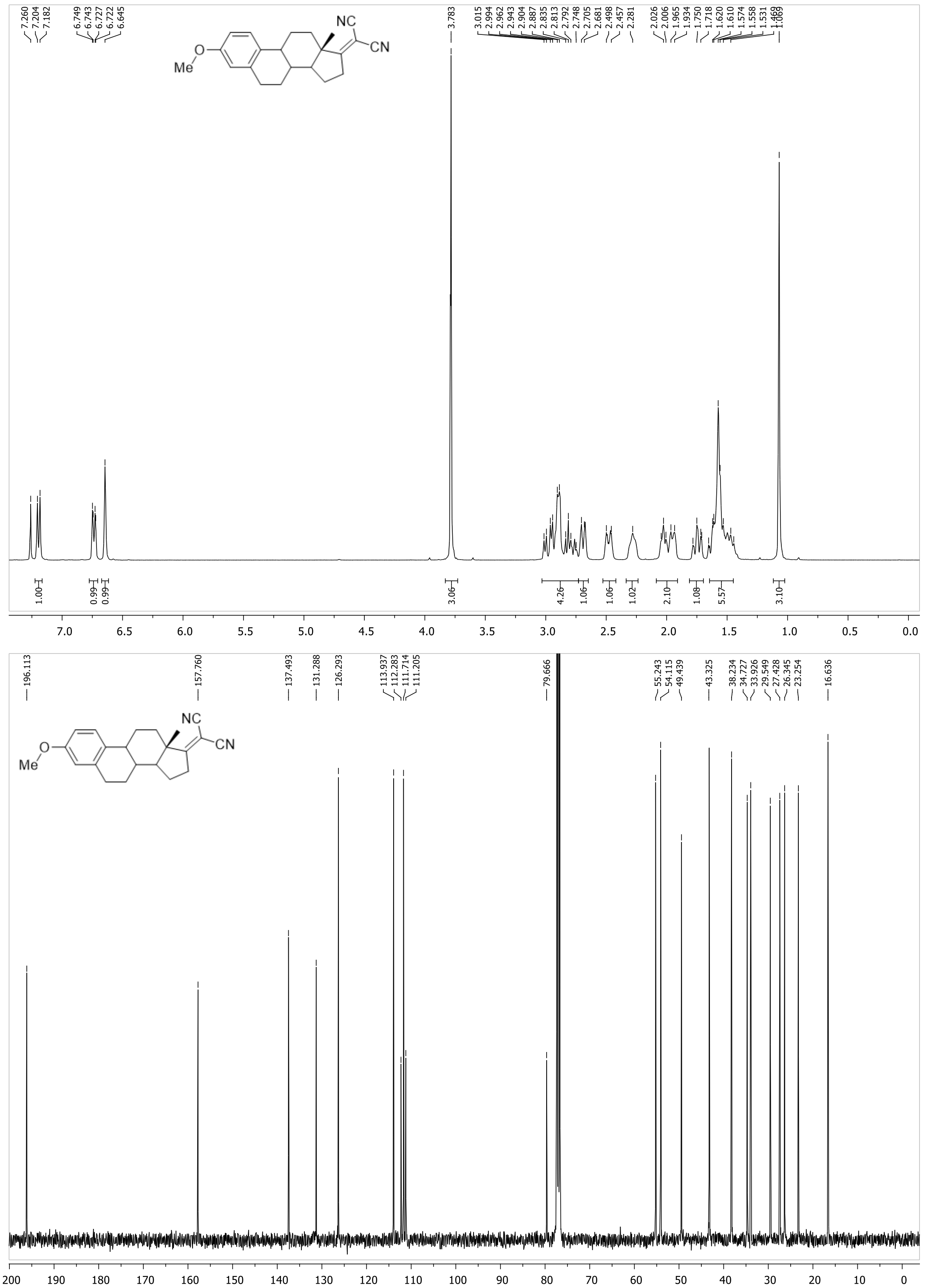

Supplementary Figure 2. ^{1}H NMR (400 MHz, $CDCl_3$) and ^{13}C NMR (100 MHz, $CDCl_3$) spectra of compound **1**.

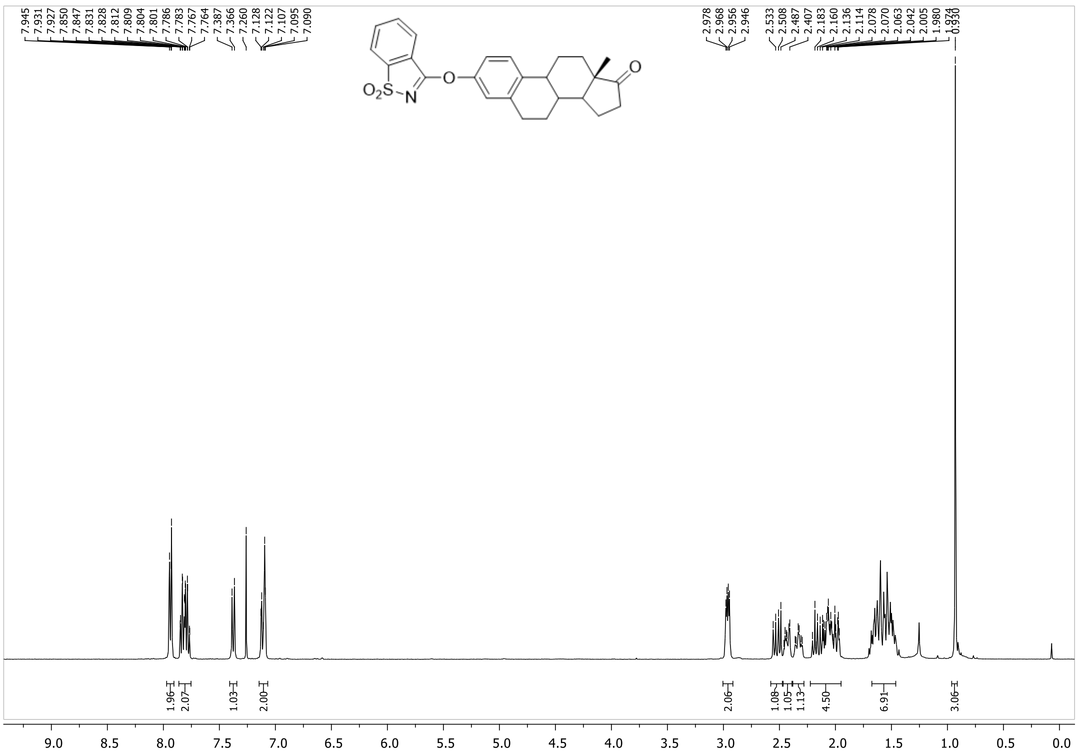

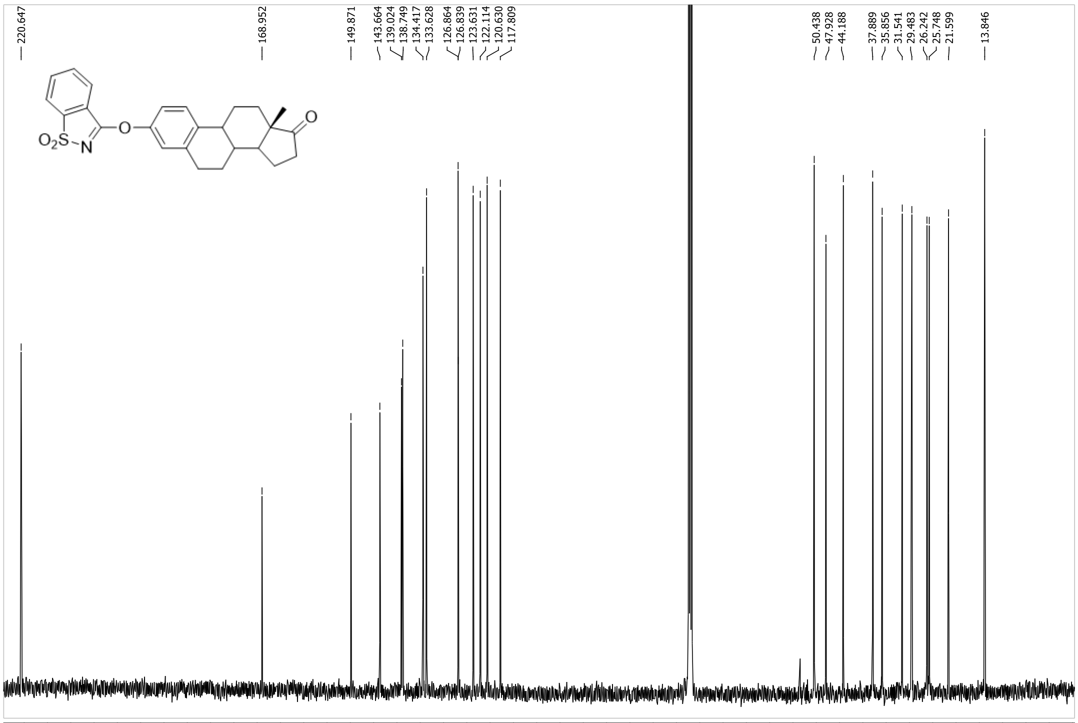

Supplementary Figure 3. ^{1}H NMR (400 MHz, $CDCl_3$) and ^{13}C NMR (100 MHz, $CDCl_3$) spectra of compound **2b**.

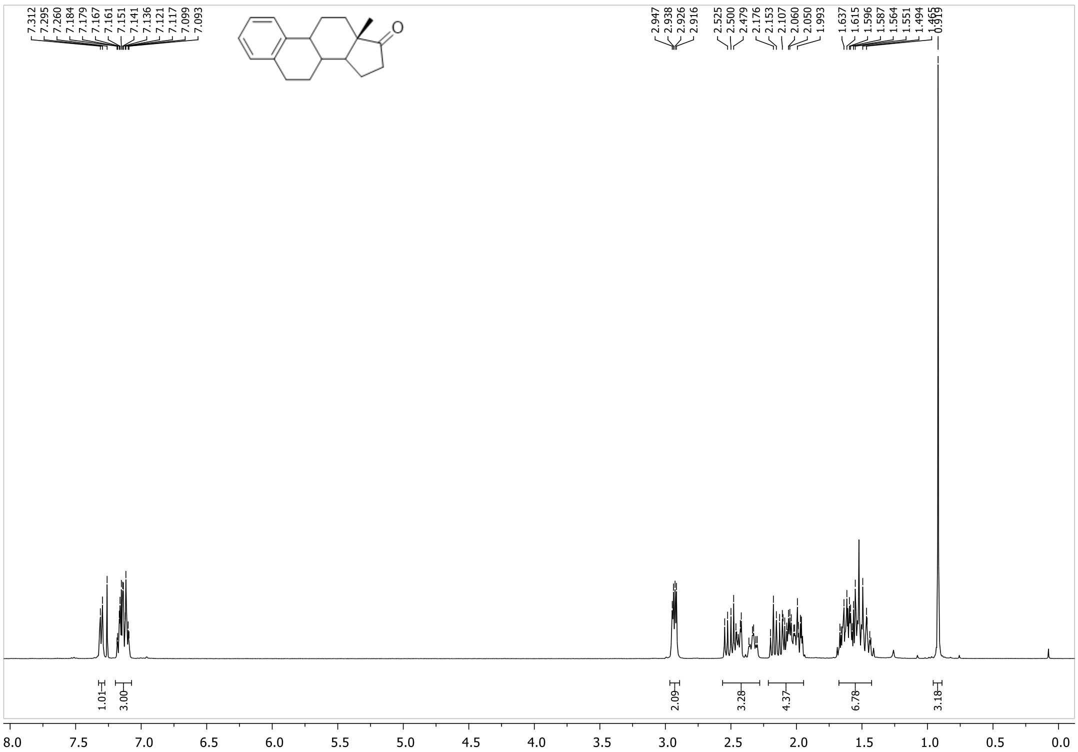

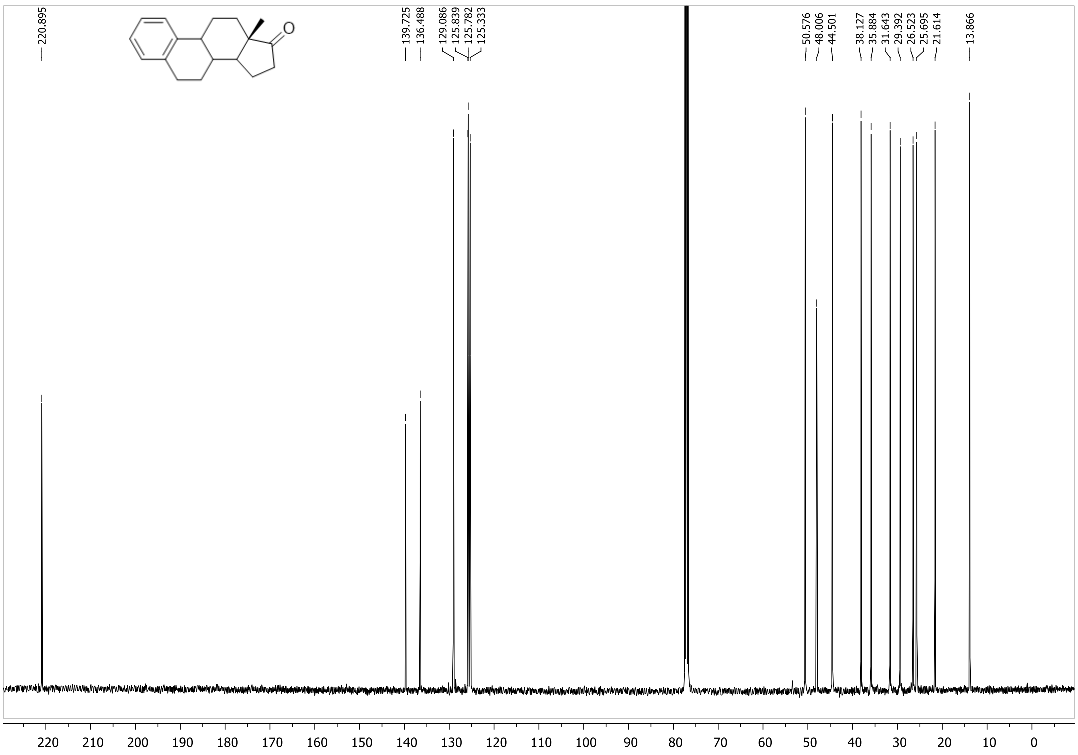

Supplementary Figure 4. ^{1}H NMR (400 MHz, $CDCl_3$) and ^{13}C NMR (100 MHz, $CDCl_3$) spectra of compound **2c**.

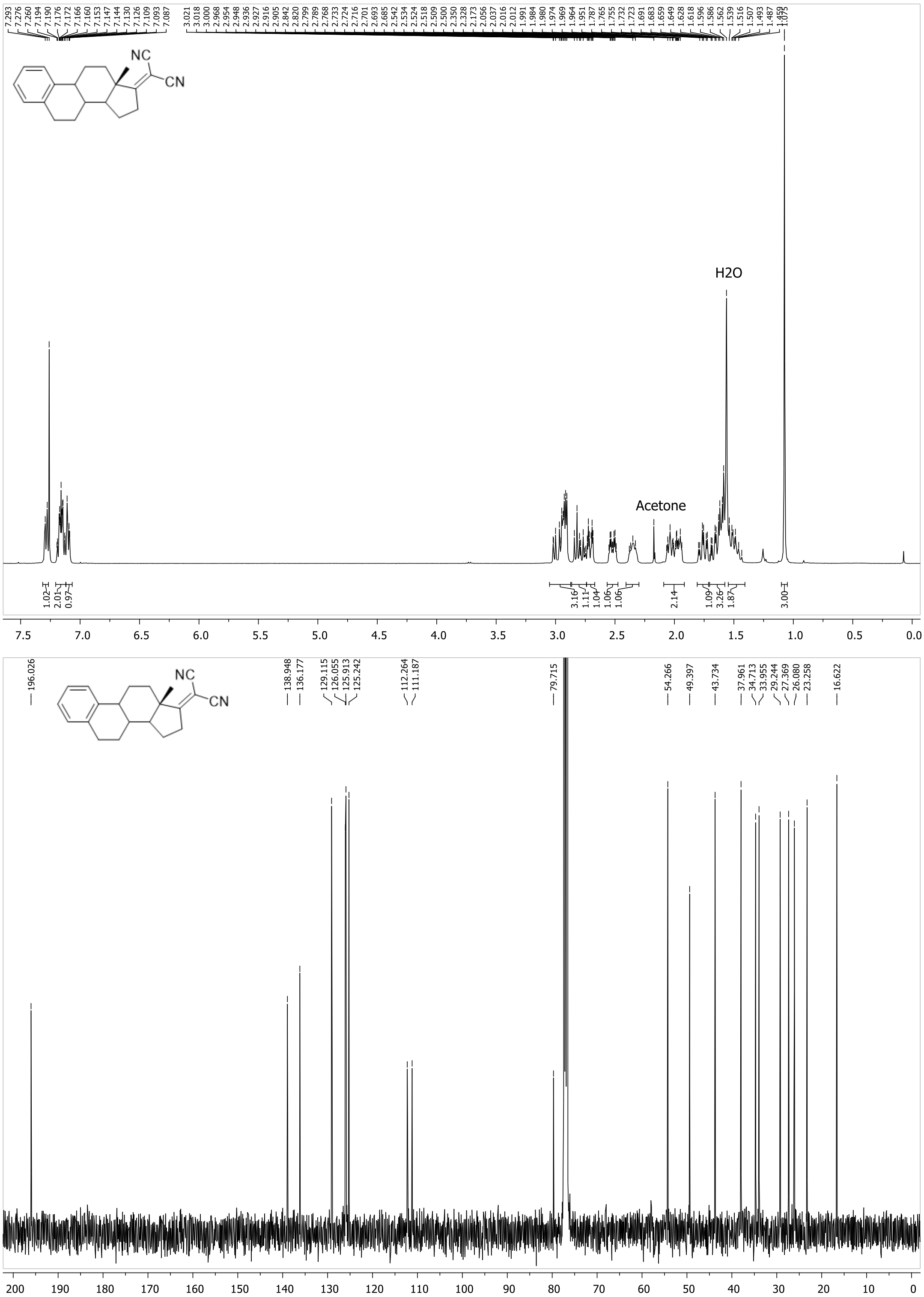

Supplementary Figure 5. ^{1}H NMR (400 MHz, $CDCl_3$) and ^{13}C NMR (100 MHz, $CDCl_3$) spectra of compound **2**.

Supplementary Note 2

Conformational searches and DFT calculations

The center-to center distance, r_c = 7.8 Å, was calculated from the center of the aromatic ring to the carbon atom bonded to the two CN groups. The edge-to-edge distance was calculated from the carbon atom #4 to the carbon atom #37.

Supplementary Table 1. Bond lengths (in Å) of benzene and dicyanoethylene moieties relevant for change recombinations, calculated by GAMESS.

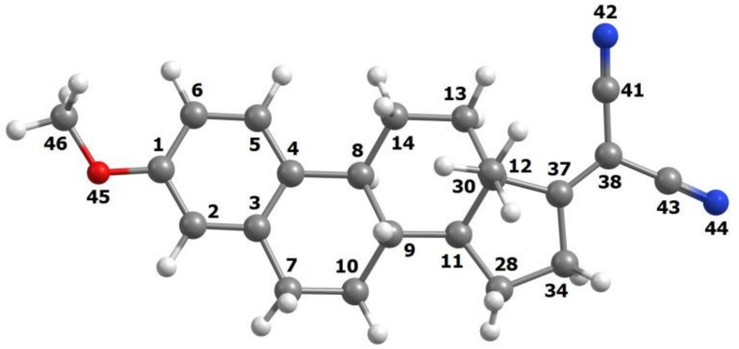

	**1**	^{1}**1**	^{1}**1**$^{\pm}$	Δq_i (^{1}**1**–^{1}**1**$^{\pm}$)	^{3}**1**$^{\pm}$
C1-C6	1.397883	1.417314	1.424782	-0.007468	1.397757
C5-C6	1.395658	1.422312	1.371685	0.050627	1.395710
C4-C5	1.399599	1.427305	1.429468	-0.002163	1.399699
C3-C4	1.413296	1.434967	1.442546	-0.007579	1.413604
C2-C3	1.394814	1.430565	1.375748	0.054817	1.394854
C1-C2	1.398082	1.419243	1.424036	-0.004793	1.397896
C4-C8	1.530642	1.516146	1.509732	0.006414	1.530923
C1-O45	1.366250	1.354455	1.320886	0.033569	1.366577
C43-N44	1.163462	1.163493	1.173919	-0.010426	1.171711
C38-C41	1.434893	1.434876	1.410905	0.023971	1.410602
C37=C38	1.360551	1.360625	1.432384	-0.071759	1.469038
C38-C43	1.436525	1.436491	1.413223	0.023268	1.410135
C41-N42	1.163464	1.163475	1.175541	-0.012066	1.171791

Supplementary Note 3

Experimental procedures

Absorption and fluorescence spectra were recorded on Shimadzu UV-2100 and Horiba-Jobin-Ivon SPEX Fluorog 3−22 spectrometers, respectively. All the fluorescence spectra were corrected for the wavelength response of the system. The fluorescence quantum yields were measured using phenol (Φ_F=0.14) as reference[1]. Phosphorescence was measured in dichloromethane:methanol (1:1, v:v) at 77 K. Charge separation (CS) and charge recombination (CR) rates were measured in the following weakly polar solvents: di-*n*-butyl ether (NBE), di-isopropyl ether (IPE), ethyl acetate (EAC), chloroform (CHF) and dichloromethane (DCM). Figures S6 presents the absorption and emission of **1** and the absorption of dMA in the presence of iPN. Figure S7 presents the absorption and emission of **2** and the absorption of oXY in the presence of iPN. The corresponding Lippert-Mataga plots of **1** and **2** are presented in Fig. S8. Figure S9 shows the thermochromism of the fluorescence of **1** and **2**.

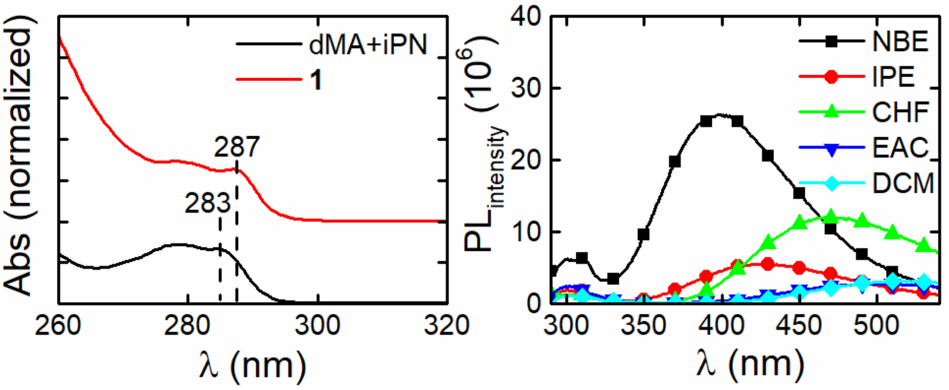

Supplementary Figure 6. Left: Absorption spectra **1** compared with the sum spectra of 3,4-dimethylanisole + isopropylidenemalononitrile (0.1 mM each) in isopropyl ether. Right: Fluorescence spectra of **1** in the solvents indicated in the plot.

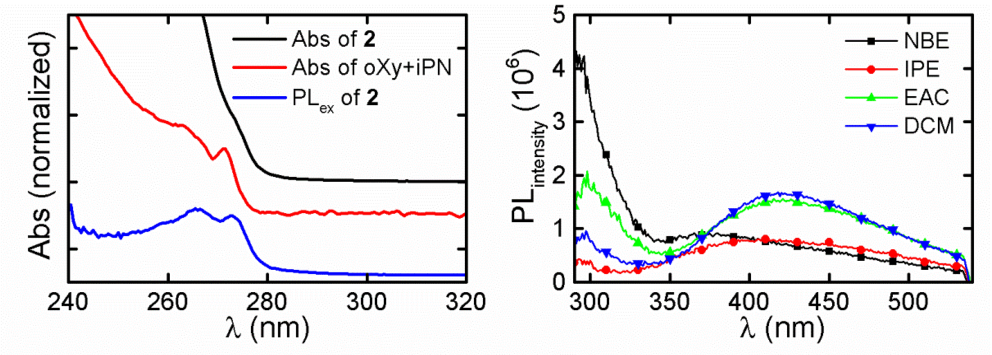

Supplementary Figure 7. Left: Absorption and fluorescence excitation spectrum of **2** in dichloromethane, compared with the sum absorption of *ortho*-xylene and isopropylidenemalononitrile (1 mM each). Right: Fluorescence spectra of **2** in the solvents indicated in the plot.

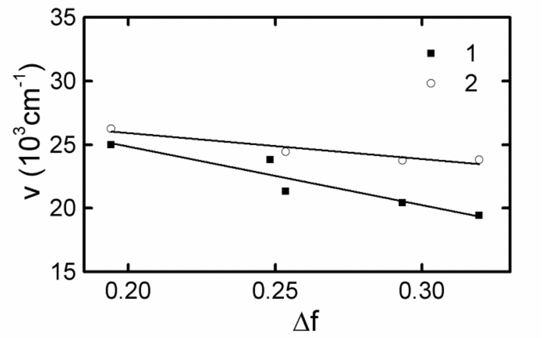

Supplementary Figure 8. Lippert-Mataga plot for **1** using fluorescence intensity maxima in NBE, IPE, CHF, EAC and DCM**,** and for **2** using fluorescence intensity maxima in NBE, CHF, EAC, DCM.

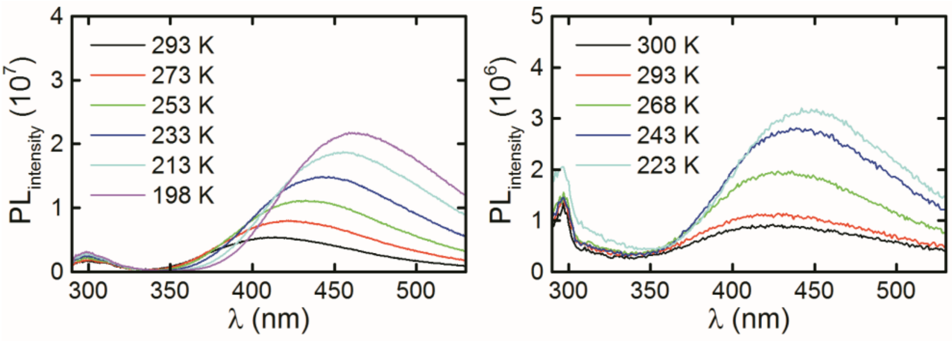

Supplementary Figure 9. Fluorescence spectra at various temperatures. Left) **1** in IPE in various temperatures (λ_{ex}=287 nm), after subtraction of the Raman band. Right) **2** in DCM (λ_{ex}=273 nm).

Supplementary Note 4

Reaction energy calculations

Donor-acceptor systems can be described as a mixture of pure locally excited (LE), pure charge separated and pure ground states

$$\Psi = c_{LE}\Psi_{LE}\left[AD^{*}\right] + c_{CS}\Psi_{CS}\left[A^{\cdot -}D^{\cdot +}\right] + c_{GS}\Psi_{GS}\left[AD\right] \tag{4.1}$$

The fluorescence from the LE state initially prepared by electronic absorption can be observed when its radiative rate is competitive with the charge separation rate. The charge-transfer (CT) emission can be observed when the contribution of Ψ_{LE} is not negligible. The emission band of charge-separated states produced by photoinduced ET depends on the polarity of the solvents and this dependence can be described by the Lippert-Mataga equation[2,3]

$$\bar{\nu}_{em} = \bar{\nu}_{em}(0) - \frac{2}{hc}\frac{\mu^2}{\rho^3}\left[f(\varepsilon) - \frac{1}{2}f(n_D)\right] \tag{4.2}$$

where μ is the dipole moment of the fluorescent state and ρ the effective radius of the solvent cavity in which the ion pair fits, $f(\varepsilon) = (\varepsilon - 1)/(2\varepsilon + 1)$ is a function of the dielectric constant and $f(n_D) = (n^2_D - 1)/(2n^2_D + 1)$ a function of the refractive index. When this cavity is ellipsoidal, the value of ρ is taken as 40% of the long axis of the cavity in which the molecule fits[4]. The observation of LE and CT emission together with the solvent dependence of the CT emission corresponding to the dipole moment of a full charge separation across the D–A distance, is a signature of photoinduced ET.

The free-energy of the charge-separated species relative to the neutral ground-state reactants, which is the negative of the free-energy for thermal charge recombination ($\Delta G^0_{CR} = -\Delta G^0_{CT}$), was calculated with the Weller expression[5,6]

$$\Delta G^0_{CT} = E^{ox}_D - E^{red}_A - \frac{e^2}{r_c\varepsilon} - \frac{e^2}{2}\left(\frac{1}{r_+} + \frac{1}{r_-}\right)\left(\frac{1}{37} - \frac{1}{\varepsilon}\right) \tag{4.3}$$

where E_D^{ox} and E_A^{red} are the electrochemical oxidation and reduction potentials of D and A as measured in acetonitrile (static dielectric constant $\varepsilon = 37$), r_c denotes the center-to-center charge separation distance, and r_+ and r_- are the average ionic radius of the created D^+ and A^- species, respectively. The free-energy for the photoinduced charge separation is $\Delta G^0_{CS} = \Delta G^0_{CT} - E^*$, where E^* is the energy of the electronically excited state from which the electron transfer occurs[5].

Supplementary Table 2. Parameters used to calculate dielectric constants employed in this study, from the dependence $\varepsilon = aT^2 + bT + c$, together with the temperature range for which equation can be used[7].

Solvent	ε (293K)	a	b	c	*T* (K)
Di-*n*-butyl ether	3.1	$5.462\cdot10^{-5}$	$-4.058\cdot10^{-2}$	$1.030\cdot10^{1}$	188-313.8
Isopropyl ether*	4.19	$7.480\cdot10^{-4}$	$-4.828\cdot10^{-1}$	$8.144\cdot10^{1}$	298.2-323.2
Chloroform	4.81	$5.680\cdot10^{-5}$	$-5.183\cdot10^{-2}$	$1.512\cdot10^{1}$	218.2-323.2
Ethyl acetate	6.08	$5.369\cdot10^{-5}$	$-5.099\cdot10^{-2}$	$1.641\cdot10^{1}$	195.2-333.3
Dichloromethane	9.00	$2.426\cdot10^{-4}$	$-1.781\cdot10^{-1}$	$4.036\cdot10^{1}$	184.1-306
Acetonitrile	37.03	$3.908\cdot10^{-4}$	$-4.028\cdot10^{-1}$	$1.215\cdot10^{1}$	229.2-333.2

*Value extrapolated

Supplementary Table 3. Data employed to calculate the free energy of charge-separated states in **1** and **2**.

Parameter	**1**	**2**	***Comment***
Center-to-center distance (r_c)	7.8 Å	7.8 Å	This work
Edge-to-edge distance (r_e)	5.9 Å	5.9 Å	This work
Average ionic radius of D^+ (r_+)	3.5 Å	3.3 Å	From molar volumes of anisole and benzene
Average ionic radius of A^- (r_-)	3.2 Å	3.2 Å	From molar volume of fumaronitrile
Oxidation potential *vs* SCE (E_{ox}^D)	1.37 V	2.09 V	From anisole (1.76 eV)[8] plus a -0.39 eV correction for the difference between benzene and o-xylene[9] as a model for **1**. From o-xylene from ref. [9] as a model for **2**.
Reduction potential *vs* SCE (E_{red}^D)	–1.69 eV	–1.69 eV	From iPN in ref. [10]
Singlet state energy (E^*)	4.29 eV	4.51 eV	From this work
Triplet state energy (E_T)	3.46 eV	3.57 eV	From the phosphorescence of 3,4-dimethylanisole, and literature value for o-xylene from ref. [1]

Supplementary Table 4. Thermodynamic data calculated using information from tables S2 and S3.

System	T (K)	ε	ΔG^0_{CS} (kcal/mol)	ΔG^0_{CR} (kcal/mol)
**1**/NBE	323	2.89	-11.4	-87.3
	308	2.98	-12.0	-86.7
	303	3.02	-12.2	-86.5
	293	3.10	-12.7	-86.0
	276	3.26	-13.6	-85.1
	258	3.47	-14.6	-84.0
	253	3.53	-14.9	-83.7
	238	3.74	-15.8	-82.9
	203	4.31	-17.8	-80.8
	193	4.50	-18.4	-80.3
**1**/IPE	293	4.19	-17.5	-81.2
**1**/CHF	328	4.23	-17.6	-81.1
	323	4.30	-17.8	-80.9
	318	4.38	-18.0	-80.6
	308	4.54	-18.5	-80.2
	293	4.81	-19.2	-79.5
	276	5.14	-20.0	-78.7
	258	5.53	-20.7	-77.9
	253	5.64	-20.9	-77.7
**1**/EAC	293	6.08	-21.7	-77.0
**1**/DCM	293	9.00	-24.7	-74.0
	276	9.68	-25.1	-73.6
	253	10.83	-25.7	-72.9
	223	12.71	-26.5	-72.2
	188	15.45	-27.3	-71.4
**2**/NBE	293	3.10	-0.4	-103.4
**2**/CHF	308	4.54	-6.5	-97.3
	293	4.81	-7.2	-96.6
	273	5.20	-8.1	-95.6
	238	6.00	-9.6	-94.1
	218	6.52	-10.4	-93.3
**2**/EAC	293	6.08	-9.8	-94.0
**2**/DCM	293	9.00	-12.9	-90.8
	276	9.68	-13.4	-90.3
	263	10.3	-13.8	-90.0
	243	11.41	-14.3	-89.4
	213	13.43	-15.1	-88.6
	188	15.45	-15.7	-88.0

Supplementary Note 5

Intersystems crossing to the triplet

The observation of the locally-excited (LE) triplet state $^{3}\mathbf{1}$ (or $^{3}\mathbf{2}$) may be accounted by alternative mechanisms. These mechanisms are briefly discussed below. The complete kinetic schemes employed to extract micro-constants from the observed decays are presented in Supplementary Section 6. The actual intersystem crossing mechanism is not critical to obtain the micro-constants, and the discussion below intends to show that mechanism A contributes with only 0.002 to the triplet quantum yields, and that mechanism B is less likely to occur than mechanisms C and D in our systems.

A. Direct intersystem crossing

$$\begin{array}{lcl} {}^{1}1 & \xrightarrow{k_{CS}} & {}^{1}1^{\pm} \\ \downarrow k_{ST} & & \\ {}^{3}1 & & \end{array} \qquad (5.1)$$

According to this mechanism, direct intersystem crossing (isc) from the LE singlet to the LE triplet, both located in the benzene ring, occurs in the chromophore model of **1** (or **2**). The 3 ns singlet lifetime of the chromophore not covalently linked to the electron acceptor, together with the triplet quantum yield of anisole, 0.64, allow us to calculate the intersystems crossing rate of $2\text{x}10^{8}$ s^{-1} in the chromophore. In molecule **1**, where the chromophore is linked to the electron acceptor, the decay constant of $^{1}\mathbf{1}$ is $\approx 10^{11}$ s^{-1}. Hence, the contribution to the triplet quantum yield by direct isc is 0.002. The triplet quantum yield measured for **1** in NBE is 0.14. Direct isc cannot account for the triplet quantum yield observed. Moreover, this mechanism should lead to LE triplets independently of the energy of the charge transfer state $^{1}\mathbf{1}^{\pm}$ (or $^{1}\mathbf{2}^{\pm}$), but for **1** in DCM, where the energy of the LE triplet is above that of $^{1}\mathbf{1}^{\pm}$, we found no evidence for the formation of $^{3}\mathbf{1}$. Our results are entirely consistent with those of Lim et al.[11] that established that the formation of LE triplet states in electron donor-acceptor complexes is only efficient when the LE triplet of a chromophore (donor or acceptor) lies below the CT transfer singlet state. Hence, the contribution of direct isc from the LE singlet to the LE triplet is small (≈0.002).

B. Intersystems crossing from the singlet charge-separated state to the locally-excited triplet

$$^{1}1 \xrightarrow{k_{CS}} {}^{1}1^{\pm} \xrightarrow{^{3}k_{CR}} {}^{3}1 \qquad (5.2)$$

Rapid intersystem crossing from the singlet CS state may take place via a spin-orbit coupling to produce the LE triplet state directly by charge recombination if the symmetry axes of donor and acceptor orbitals are nearly perpendicular to each other. This mechanism was originally proposed by Okada et al.[12] to explain the sub-nanosecond generation of LE triplet states in flexible intramolecular exciplexes. It was also used to explain the observation of LE triplets in donor-acceptor molecules where the donor and acceptor orbitals are restricted to be nearly perpendicular, as the π-electron transfer generates the necessary torque to flip the electron spin[13-15].

The donor orbital of $^{1}\mathbf{1}^{\pm}$ is located in the dicyanoethene and the acceptor orbital is located in the benzene ring. As shown in the scheme below, these axes are not perpendicular and spin-orbit coupling is not expected to operate efficiently in this case. Moreover, this should lead to mono-exponential decays of $^{1}\mathbf{1}^{\pm}$ when the energy of $^{3}\mathbf{1}$ is above that of $^{1}\mathbf{1}^{\pm}$ (which are observed) and also when the energy of $^{3}\mathbf{1}$ is much lower than that of $^{1}\mathbf{1}^{\pm}$ (which are not observed). The energies of $^{1}\mathbf{2}^{\pm}$ in the various solvents are always much higher than those of $^{3}\mathbf{2}$, but the decays of $^{1}\mathbf{2}^{\pm}$ are bi-exponential, which further argues against rapid intersystem crossing from $^{1}\mathbf{1}^{\pm}$ to $^{3}\mathbf{1}$ (or $^{1}\mathbf{2}^{\pm}$ to $^{3}\mathbf{2}$).

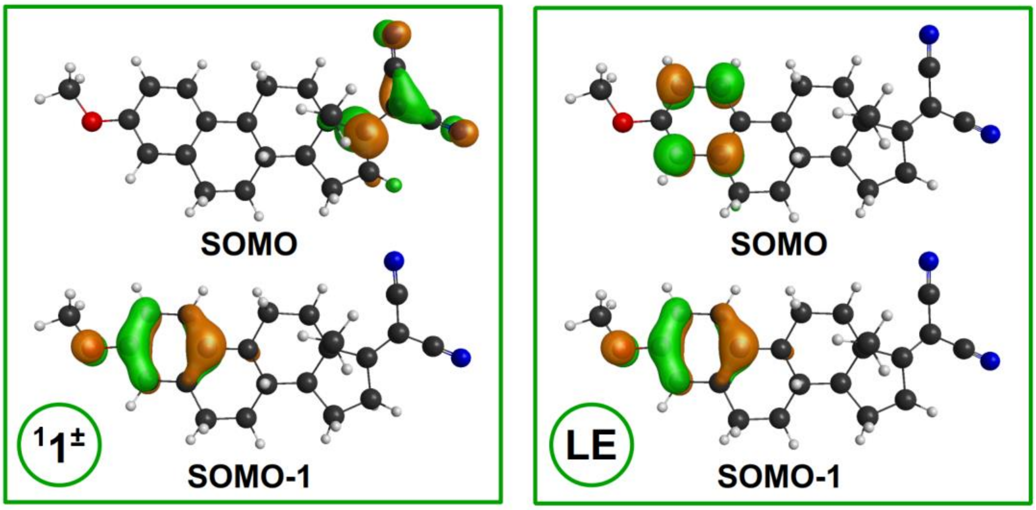

C. Hyperfine coupling of singlet and triplet charge-separated states

$$^{1}1 \xrightarrow{k_{CS}} {}^{1}1^{\pm} \underset{^{3}k_{isc}}{\overset{^{1}k_{isc}}{\rightleftarrows}} {}^{3}1^{\pm} \xrightarrow{^{3}k_{CR}} {}^{3}1 \tag{5.3}$$

In this mechanism, fast intersystem crossing between **^{1}1**$^{\pm}$ and **^{3}1**$^{\pm}$, followed by triplet charge recombination to the LE triplet, is triggered by electron-nuclear hyperfine coupling.

Hyperfine coupling is known to bring the electron spins to a triplet alignment with a rate constant $k_{\mathrm{isc}} \approx 10^{8}$ s^{-1} in solvent-separated ion pairs, where center-to-center distance between donor and acceptor is $r_c \approx 7$ Å [16], which is similar to the distance found in **1**: $r_c = 7.8$ Å. This interaction mechanism is plausible on the basis of r_c. However, it is also known that the hyperfine interaction can only couple the singlet and triplet states effectively when the value of the hyperfine interaction, calculated for each radical i from

$$B_i = \left[\sum_i a_{ik}^2 I_k \left(I_k + 1 \right) \right]^{1/2} \tag{5.4}$$

where a_{ik} are the hyperfine coupling constants in radical i and I_{k} are the nuclear spins, is comparable to the singlet–triplet energy splitting ΔE_{ST}. Organic radical ions typically have $B_{\mathrm{i}} \approx 1$ mT [17,18]. Paddon-Row and Shephard[19] studied singlet-triplet energy gaps between charge-separated states in a series of aromatic (donor) – rigid bridge – dicyanovinyl (acceptor) as a function of the number of single CC bonds in the bridge. TD-DFT with the B3P86 basis set gave singlet-triplet energy gaps of 1.3 and 0.26 cm^{-1} for bridges with 10 and 12 CC bonds, respectively, for which the following experimental values are available: 2.1 and 0.46 cm^{-1}. The same calculations predict $\Delta E_{\mathrm{ST}} \approx 97.2$ cm^{-1} ≈ 300 cal/mol ≈ 100 T for a bridge with 5 CC bonds, which is the case of molecules **1** and **2** in our work. Hyperfine coupling is not plausible on the basis of such a relatively large ΔE_{ST}. However, this singlet-triplet energy gap (the triplet state is more stable than the singlet state) was calculated as the difference between overestimated energies of singlet and triplet CT states and may be subject to some error.

The kinetic data analysis in Section 6 is based on rate constants and, consequently, on free energies. Taking the degeneracy of the three triplet states in consideration, and assuming that hyperfine coupling is identical for all of them, the singlet-triplet exchange can be written

$$^{1}1^{\pm} \underset{\frac{1}{3}k_{hfc}}{\overset{k_{hfc}}{\rightleftarrows}} {}^{3}1^{\pm} \tag{5.5}$$

and $\Delta G_{\mathrm{ST}}^{0} = -\Delta E_{\mathrm{ST}} - RT\ln(3)$. Under these assumptions, $\Delta E_{\mathrm{ST}} = 0$ would yield $\Delta G^{0} = -0.6$ kcal/mol at T = 293 K. The value employed in Section 6 was $\Delta G^{0} = -0.3$ kcal/mol and the rate constants reported are $^{1}k_{\mathrm{isc}}$ and $^{3}k_{\mathrm{isc}}$. If the hyperfine coupling mechanism dominates isc (i.e. $\Delta E_{\mathrm{ST}} \approx 0$), $^{3}k_{\mathrm{isc}} \approx 0.6\ k_{\mathrm{hfc}}$.

In summary, the value of ΔE_{ST} is not known with sufficient accuracy to preclude hyperfine coupling from contributing to fast isc between $^{1}\mathbf{1}^{\pm}$ and $^{3}\mathbf{1}^{\pm}$, and it is of the size of statistical corrections on the rates and on ΔG^{0}. Hence, we cannot exclude hyperfine coupling from contributing to isc in the CT states.

D. Spin-orbit coupling of singlet and triplet charge-separated states

$$^{1}1 \xrightarrow{k_{CS}} {}^{1}1^{\pm} \underset{^{3}k_{isc}}{\overset{^{1}k_{isc}}{\rightleftarrows}} {}^{3}1^{\pm} \xrightarrow{^{3}k_{CR}} {}^{3}1 \qquad (5.6)$$

Spin-orbit coupling is not usually operative between singlet and triplet CT states because the orbitals involved in both states are the same and the matrix element vanishes, $\langle ^{1}CT|H_{SOC}|^{3}CT\rangle=0$ [11]. However, the 82° rotation of the donor orbital from the singlet to the triplet CT state, which brings the donor and acceptor orbitals to nearly perpendicular orientations, may provide a change in orbital angular momentum that generates the necessary torque to flip the electron spin.

Significant matrix elements of spin-orbit coupling between ^{1}CT and ^{3}CT states can occur and enhance isc when it is concurrent with a spatial change in the orbital and/or in the presence of σ-π overlap[11]. Both cases are enabled by the rotation of the dicyanoethene moiety in $^{1}\mathbf{1}^{\pm}\rightarrow{}^{3}\mathbf{1}^{\pm}$ (or $^{1}\mathbf{2}^{\pm}\rightarrow{}^{3}\mathbf{2}^{\pm}$) as shown in the scheme below. Hence, spin-orbit coupling may accelerate the interconversion between ^{1}CT and ^{3}CT, and open a channel to the LE triplet state when it is energetically accessible from the CT states.

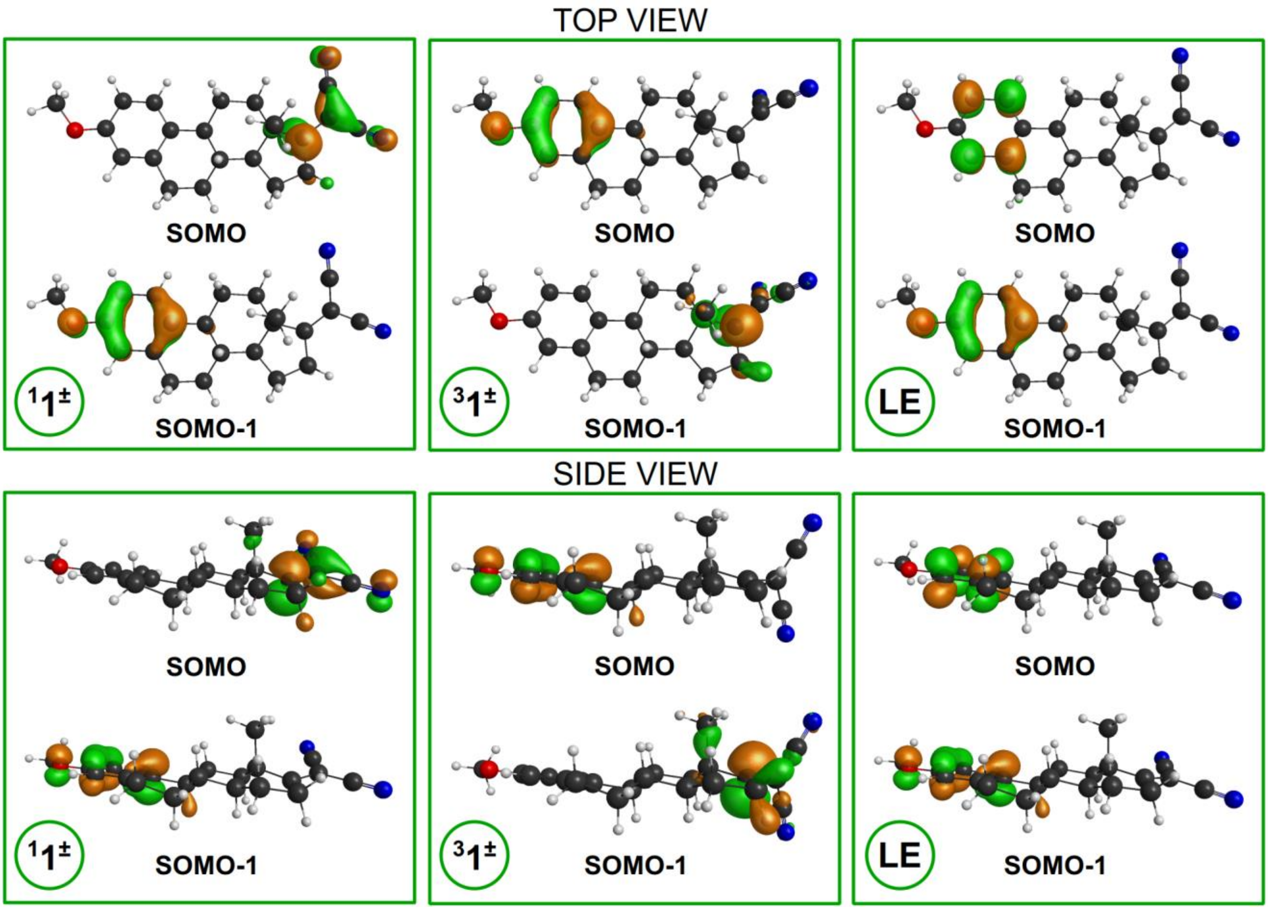

Supplementary Note 6

Analysis of transient absorption and single-photon counting data

The solution of the modified Birks mechanism presented in Eq. (5C) the main text:

$$\frac{\mathrm{d}\left[{}^{1}\mathrm{CT}\right]}{\mathrm{d}t} = {}^{3}k_{isc}\left[{}^{3}\mathrm{CT}\right] - {}^{1}k_{isc}\left[{}^{1}\mathrm{CT}\right] - {}^{1}k_{CR}\left[{}^{1}\mathrm{CT}\right]$$
$$\frac{\mathrm{d}\left[{}^{3}\mathrm{CT}\right]}{\mathrm{d}t} = {}^{1}k_{isc}\left[{}^{1}\mathrm{CT}\right] - {}^{3}k_{isc}\left[{}^{3}\mathrm{CT}\right] - {}^{3}k_{CR}\left[{}^{3}\mathrm{CT}\right] \qquad (6.1)$$

is equivalent to that of the Birks mechanism[20]

$$I_{1\mathrm{CT}} = a_{11}e^{-\lambda_1 t} + a_{12}e^{-\lambda_2 t} = \frac{\left[{}^{1}\mathrm{CT}\right]_0}{\lambda_2 - \lambda_1}\left\{(\lambda_2 - k_x)e^{-\lambda_1 t} + (k_x - \lambda_1)e^{-\lambda_2 t}\right\}$$
$$I_{3\mathrm{CT}} = a_{21}e^{-\lambda_1 t} + a_{22}e^{-\lambda_2 t} = \frac{{}^{1}k_{isc}\left[{}^{1}\mathrm{CT}\right]_0}{\lambda_2 - \lambda_1}\left(e^{-\lambda_1 t} - e^{-\lambda_2 t}\right) \qquad (6.2)$$

where

$$\lambda_1 = \frac{1}{2}\left[\left(k_x + k_y\right) - \sqrt{\left(k_x - k_y\right)^2 + 4\,{}^{1}k_{isc}\,{}^{3}k_{isc}}\right]$$
$$\lambda_2 = \frac{1}{2}\left[\left(k_x + k_y\right) + \sqrt{\left(k_x - k_y\right)^2 + 4\,{}^{1}k_{isc}\,{}^{3}k_{isc}}\right] \qquad (6.3)$$

with

$$k_x = {}^{1}k_{isc} + {}^{1}k_{CR}$$
$$k_y = {}^{3}k_{isc} + {}^{3}k_{CR} \qquad (6.4)$$

and

$$\lambda_1\lambda_2 = k_x k_y - {}^{1}k_{isc}\,{}^{3}k_{isc}$$
$$\lambda_1 + \lambda_2 = k_x + k_y \qquad (6.5)$$

Only the emission from ^{1}CT is observed. Hence, we can use the relation

$$\frac{a_{11}}{a_{12}} = \frac{\lambda_2 - k_x}{k_x - \lambda_1} \qquad (6.6)$$

to obtain

$$k_x = \frac{a_{11}/a_{12}\,\lambda_1 + \lambda_2}{1 + a_{11}/a_{12}} \qquad (6.7)$$

Hence,

$${}^{1}k_{isc} = \frac{a_{11}/a_{12}\,\lambda_1 + \lambda_2}{1 + a_{11}/a_{12}} - {}^{1}k_{CR}$$
$${}^{3}k_{isc} = \frac{k_x\left(\lambda_1 + \lambda_2 - k_x\right) - \lambda_1\lambda_2}{{}^{1}k_{isc}} \qquad (6.8)$$
$${}^{3}k_{CR} = \lambda_1 + \lambda_2 - k_x - {}^{3}k_{isc}$$

The original Birks mechanism applied to excimer kinetics can be easily solved because the quenching of the monomer is a bimolecular process that can be neglected at very high dilutions to obtain its decay rate (corresponding to $^{1}k_{\mathrm{CR}}$ in our mechanism). This is not the case in intramolecular systems **1** and **2**. In order to solve the equations above, we need to assume an additional relation. The intersystem crossing in the CT species can only compete with the decays of the CT species if their energies are similar, i.e., if $\Delta E_{\mathrm{ST}} \approx 0$. We solved the equations above using $\Delta G_{\mathrm{ST}} = -0.3$ kcal/mol and assumed this free-energy difference to be independent of the temperature.

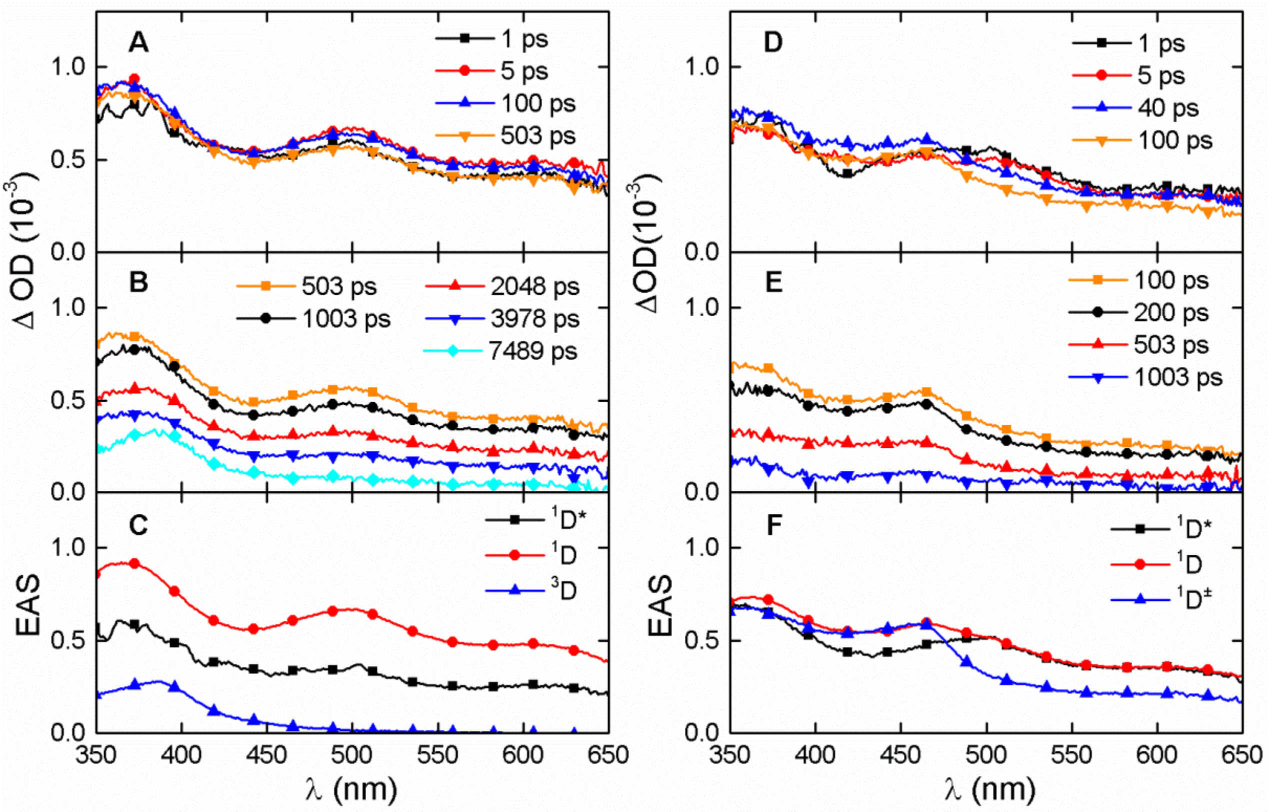

Supplementary Figure 10. Top. Transient difference absorption spectra of 3,4-dimethylanisole in isopropyl ether in short and long time scales, either in the absence (A, B) and presence of 0.1 M isopropylidenemalononitrile (D, E). Evolution associated spectra in the absence (C) and presence (F) of acceptor are presented.

Supplementary Table 5. Lifetimes of **1** obtained by femtosecond transient absorption, from target analysis.

**Solvent**	**T (K)**	τ_{CS}	τ_1	τ_2
NBE	323	8.23 ps	0.93 ns	5.41 ns
	308	6.62 ps	0.70 ns	8.09 ns
	293	8.42 ps	1.76 ns	10.26 ns
	276	8.40 ps	1.21 ns	14.29 ns
	253	7.85 ps	0.52 ns	17. 87 ns
IPE	293	8.70 ps	1.40 ns	9.43 ns
CHF*	323	3.97 ps	-	10.2 ns
	293	6.60 ps	-	14.68 ns
	276	6.83 ps	-	13.81 ns
	253	7.96 ps	-	13.26 ns
EAC	293	8.23 ps	-	10.15 ns
DCM	293	4.06 ps	-	13.82 ns
	276	3.95 ps	-	11.58 ns
	253	4.34 ps	-	9.53 ns
	223	4.07 ps	-	9.45 ns
	188	5.01 ps	-	5.65 ns

*An additional decay lifetime would improve the fitting but with the spectrum unrelated to all other spectra observed in this work. Solvent purity significantly changes the contribution of this additional lifetime to the fitting. We consider that additional lifetime is probably related to an impurity and ignored it in the treatment of the data.

Supplementary Table 6. Lifetimes of **2** obtained by femtosecond transient absorption, from target analysis.

**Solvent**	**T (K)**	τ_{CS}	τ_1	τ_2	τ_{tail}
NBE	293	46.23 ps	–	0.22 ns	6.47 ns
CHF	293	12.09 ps	–	1.0 ns	13.53 ns
EAC	293	39.15 ps	0.45 ns	4.90 ns	
DCM	293	9.31 ps	0.20 ns	6.96 ns	

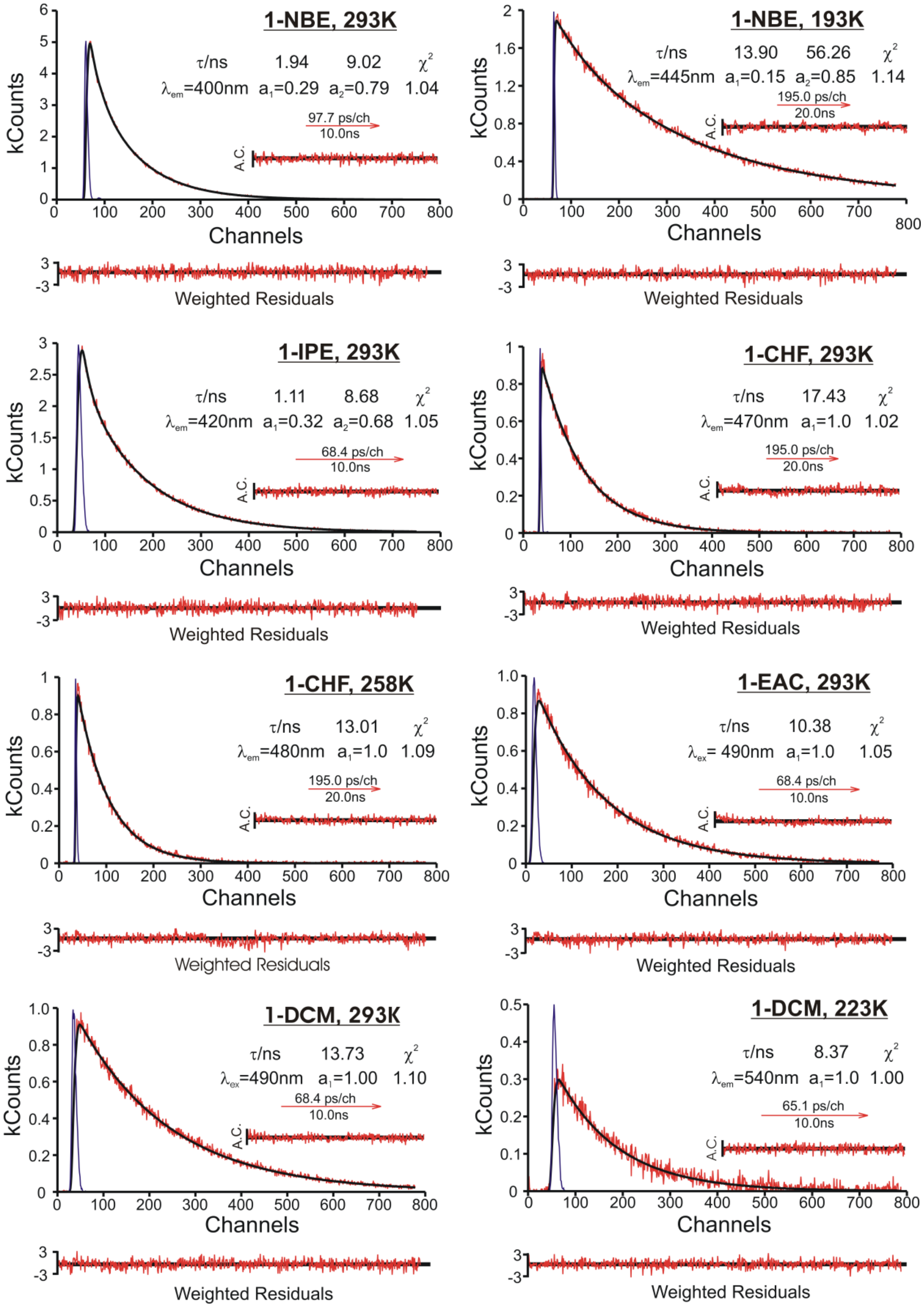

Supplementary Figure 11. Single-photon counting of charge transfer emission of **1** in different solvents and different temperatures.

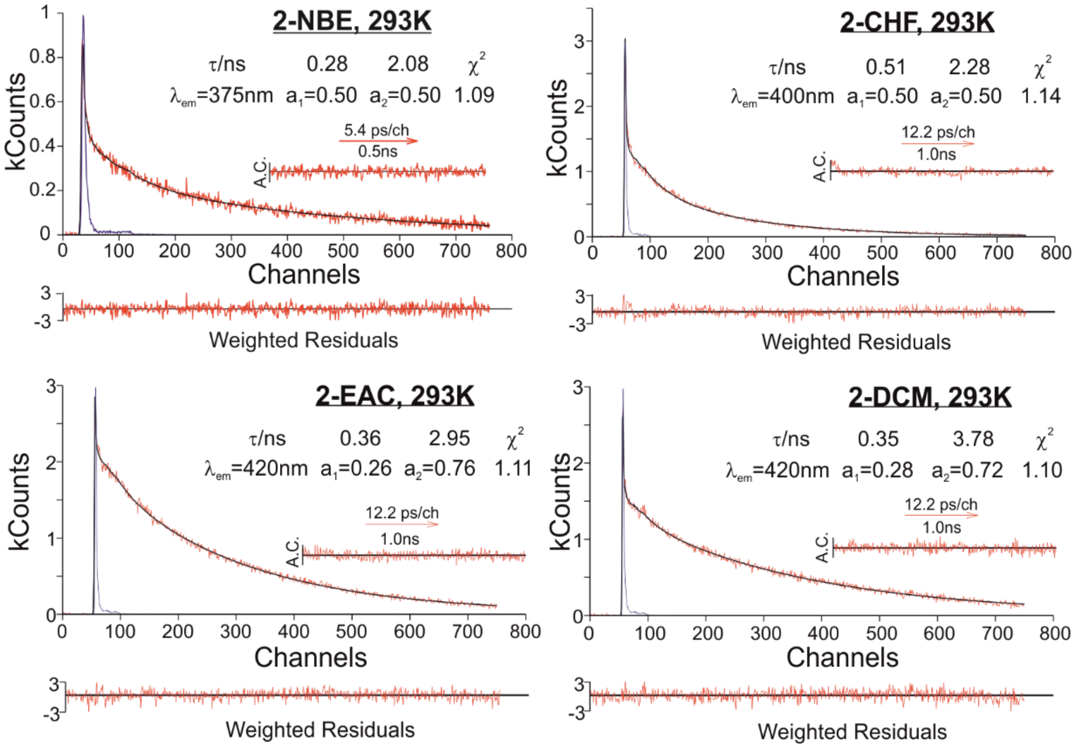

Supplementary Figure 12. Single-photon counting of charge transfer emission of **2** in different solvents, at room temperature.

Supplementary Table 7. Charge recombination lifetimes of $^{1}\mathbf{1}^{\pm}$ obtained by SPC in various solvents, measured close to the maximum of the fluorescence emission.

Solvent	T (K)	λ_{em} (nm)	τ_1 / a_1	τ_2 / a_2
NBE	323	390	0.8 ns / 21%	4.3 ns / 79%
	308	395	1.3 ns / 24%	6.5 ns / 76%
	303	395	1.0 ns / 38%	10.0 ns / 62%
	293	400	1.9 ns / 29%	9.0 ns / 71%
	276	410	2.9 ns / 25%	11.4 ns / 75%
	258	420	3.7 ns / 16%	13.1 ns / 84%
	253	410	2.9 ns/ 23%	16.1 ns / 77%
	238	435	4.9 ns / 19%	22.8 ns / 81 %
	203	445	8.8 ns / 15%	46.8 ns / 85%
	193	445	13.9 ns / 15%	56.3 ns / 85%
IPE	293	420	1.1 ns / 32%	8.7 ns / 68%
CHF	328	450	-	16.7 ns / 100%
	318	455	-	16.7 ns / 100%
	308	460	-	17.7 ns /100%
	293	470	-	17.4 ns / 100%
	276	480	-	15.2 ns / 100%
	258	480	-	13.0 ns / 100%
EAC	293	490	-	10.4 ns / 100%
DCM	293	490	-	13.7 ns / 100%
	276	520	-	11.2 ns / 100%
	253	525	-	9.3 ns / 100%
	223	540	-	8.4 ns / 100%
	188	545	-	5.6 ns / 100%

Supplementary Table 8. Charge recombination lifetimes of $^{1}2^{\pm}$ obtained by SPC in various solvents, at different temperatures.

Solvent	T (K)	λ_{em} (nm)	τ_1 / a_1	τ_2 / a_2
NBE	293	375	0.28 ns / 50%	2.08 ns / 50%
	308	395	0.52 ns / 66%	2.28 ns / 34%
	293[b]	400	0.44±0.14 ns / 49%	2.15±0.16 /51 %
CHF[a]	273	405	0.33 ns / 42%	2.60 ns / 58%
	238	417	0.23 ns / 39%	3.41 ns / 61%
	218	423	0.29 ns / 32%	3.76 ns / 68%
EAC	293[b]	420	0.34±0.04 ns / 26%	3.15±0.17 ns / 74%
DCM	293[b]	420	0.35±0.06 ns / 30%	3.86±0.20 ns / 70%
	276	425	0.38 ns / 28%	4.14 ns / 72%
	263	430	0.46 ns / 26%	4.32 ns / 74%
	243	441	0.41 ns / 26%	4.50 ns / 74%
	213	449	0.47 ns / 25%	4.58 ns / 75%
	188	456	0.47 ns / 26%	4.61 ns / 74%

[a] All measurements were performed in the same day using cryostat Optistat DN2, except measurement at 308K, which was performed in the sample holder Flash 300.
[b] Average and sample standard deviation of three to six independent measurements.

Supplementary Table 9. Micro-constants extracted from SPC data using the adapted Birks mechanism with ΔE_{ST}=0.3 kcal/mol.

System	T (K)	$^1k_{CR}$ (s^{-1})	$^3k_{isc}$ (s^{-1})	$^1k_{isc}$ (s^{-1})	$^3k_{CR}$ (s^{-1})
**1** / NBE	323	1.24E+08	5.17E+08	3.24E+08	5.05E+08
	308	9.32E+07	3.50E+08	2.14E+08	2.90E+08
	303	1.05E+08	5.74E+08	3.49E+08	9.21E+07
	293	8.66E+07	2.38E+08	1.42E+08	1.60E+08
	276	6.82E+07	1.45E+08	8.40E+07	1.32E+08
	258	5.38E+07	9.56E+07	5.32E+07	1.45E+08
	253	3.81E+07	1.62E+08	8.93E+07	1.22E+08
	238	2.82E+07	8.66E+07	4.59E+07	8.90E+07
	203	1.25E+07	4.81E+07	2.29E+07	5.18E+07
	193	1.28E+07	2.86E+07	1.31E+07	3.53E+07
**1** / IPE	293	8.20E+07	4.59E+08	2.74E+08	1.77E+08
**2** / NBE	293	8.38E+08	2.00E+09	1.19E+09	2.05E+07
**2** / CHF	308	8.62E+08	9.01E+08	5.52E+08	4.76E+07
	293[a]	6.74E+08	1.30E+09	7.74E+08	1.93E+08
	273	5.09E+08	1.72E+09	9.91E+08	1.93E+08
	238	4.22E+08	2.71E+09	1.44E+09	7.04E+08
	218	2.34E+08	2.10E+09	1.05E+09	3.32E+08
**2** / EAC	293[a]	1.20E+08	1.52E+09	9.07E+08	7.50E+08
**2** / DCM	293[a]	1.25E+08	1.57E+09	9.35E+08	5.37E+08
	276	9.99E+07	1.42E+09	8.20E+09	5.37E+08
	263	9.53E+07	1.13E+09	6.38E+08	5.39E+08
	243	7.58E+07	1.36E+09	7.28E+08	5.63E+08
	213	1.14E+08	1.18E+09	5.79E+08	4.76E+08
	188	1.48E+08	1.25E+09	5.58E+08	3.94E+08

[a] Average of three to six independent measurements.

Supplementary Table 10. Free energies and ET rate constants.

System	*T* (K)	ΔG^0_{CS} (kcal/mol)	$^1k_{CS}$ (10^{11} s^{-1})	ΔG^0_{CR} (kcal/mol)	$^1k_{CR}$ (10^8 s^{-1})	$1/\tau_2$ (10^8 s^{-1})
**1**/NBE	323	-11.4	1.22	-87.3	1.24	1.85
	308	-12.0	1.51	-86.7	0.93	1.24
	303	-12.2		-86.5	1.05	
	293	-12.7	1.19	-86.0	0.87	0.98
	276	-13.6	1.19	-85.1	0.68	0.70
	258	-14.6		-84.0	0.54	
	253	-14.9	1.27	-83.7	0.38	0.56
	238	-15.8		-82.9	0.28	
	203	-17.8		-80.8	0.13	
	193	-18.4		-80.3	0.13	
**1**/IPE	293	-17.5	1.15	-81.2	0.82	1.06
**1**/CHF	328	-17.8	2.52 [a]	-81.1	0.60	0.98[a]
	318	-18.0		-80.6	0.60	
	308	-18.5		-80.2	0.56	
	293	-19.2	1.52	-79.5	0.58	0.68
	276	-20.0	1.46	-78.7	0.66	0.72
	258	-20.7	1.26 [b]	-77.9	0.77	0.75[b]
**1**/EAC	293	-21.7	1.22	-77.0	0.96	0.96
**1**/DCM	293	-24.7	2.46	-74.0	0.73	0.72
	276	-25.1	2.53	-73.6	0.89	0.86
	253	-25.7	2.30	-72.9	1.08	1.05
	223	-26.5	2.46	-72.2	1.19	1.06
	188	-27.3	2.00	-71.4	1.79	1.77
**2**/NBE	293	-0.4	0.22	-103.4	8.38	46.08
	253	-0.4	0.07	-103.4		30.67
**2**/CHF	308	-6.5		-97.3	8.62	
	293	-7.2	0.83	-96.6	6.74±1.25[c]	10.00
	273	-8.1		-95.6	5.09	
	238	-9.6		-94.1	4.22	
	218	-10.4		-93.3	2.34	
**2**/EAC	293	-9.8	0.26	-94.0	1.20±0.27[c]	2.04
**2**/DCM	293	-12.9	1.07	-90.8	1.25±0.72[c]	1.44
	276	-13.4		-90.3	1.00	
	263	-13.8		-90.0	0.95	
	243	-14.3		-89.4	0.76	
	213	-15.1		-88.6	1.14	
	188	-15.7		-88.0	1.48	

[a]At 323 K where the transient absorption of **1**/CHF has a feature nearly absent at lower temperatures (Fig. S13), which can be assigned to a complex formed between the anionic groups of ^{1}**1**$^{\pm}$ and a chlorine atom, on the basis of literature reports on similar species,[21] on the increase of the intensity of this feature with the removal of the stabilizer from the solvent, and on the decrease of this feature when the solvent is kept at lower temperatures. [b]At 253 K. [c]Average and sample standard deviation of three to six independent measurements, representative of the error bars of rate constants obtained by single photon counting.

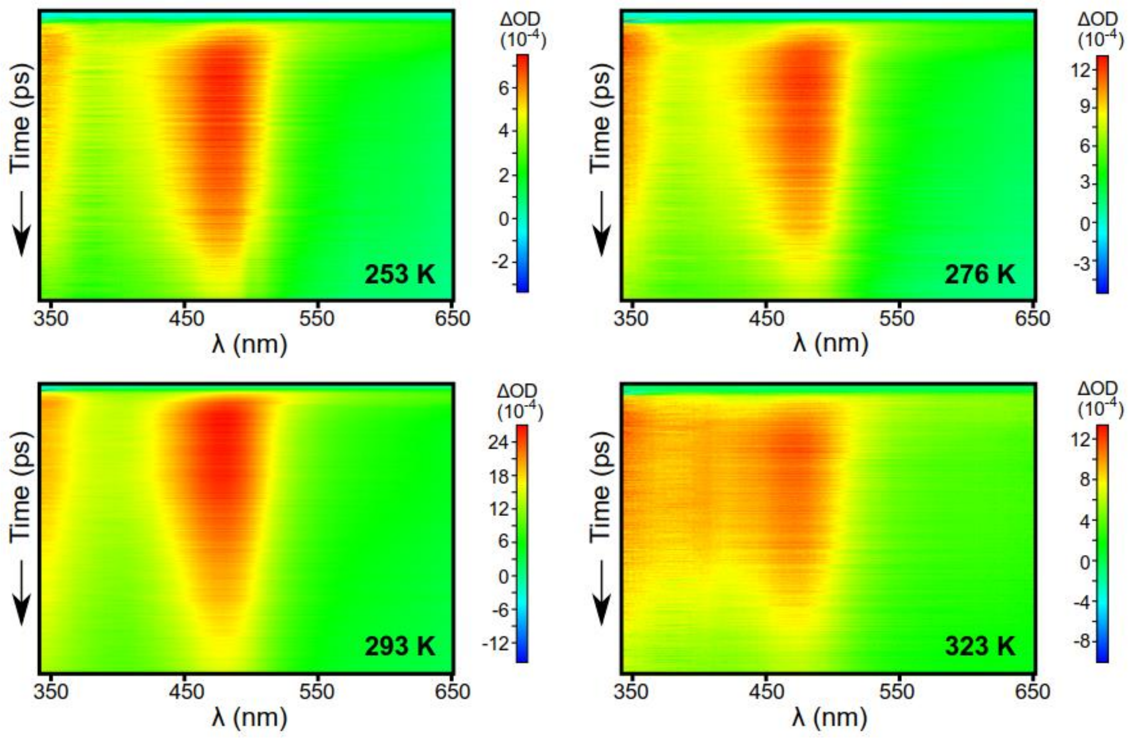

Supplementary Figure 13. 2D images of data collected for **1** in chloroform and various temperatures, using fs pump-probe spectroscopy.

Supplementary Note 7

Electron transfer rate constants

The ET rates can be expressed as the product between the electronic coupling strength between donor and acceptor (V) and a Franck-Condon weighted density of states ($FCWD$)

$$k = \frac{2\pi}{\hbar}\left|V^2\right| FCWD \qquad (7.1)$$

where V is related to the probability of electron tunneling from donor to acceptor in the activated complex. For the case of displaced oscillators with frequencies ω_i and reduced masses μ_i identical in the initial and final states, the Franck-Condon factor is a convolution between line shape integrals corresponding to the contributions of the solvent mode (subscript s) and of one higher frequency molecular vibrational mode (subscript v), that can be expressed as[22,23]

$$FCWD = \frac{1}{\hbar\omega_s}\exp\left[-S_s\left(2\bar{n}_s+1\right)-S_v\left(2\bar{n}_v+1\right)\right]\sum_{m=-\infty}^{+\infty}\left(\frac{\bar{n}_s+1}{\bar{n}_s}\right)^{p(m)/2}$$
$$\times I_{|p(m)|}\left(2S_s\sqrt{\bar{n}_s\left(\bar{n}_s+1\right)}\right)\left(\frac{\bar{n}_v+1}{\bar{n}_v}\right)^{m/2} I_m\left(2S_v\sqrt{\bar{n}_v\left(\bar{n}_v+1\right)}\right) \qquad (7.2)$$

where $\Delta E = -\Delta G^0$ because the frequencies were assumed not to change, $p(m) = \left(\Delta E - m\hbar\omega_v\right)/\hbar\omega_s$ is taken as an integer closest to the value of $p(m)$, $I_x(z)$ is the modified Bessel function, the average quantum number of an ensemble of identical oscillators in thermal equilibrium is

$$\bar{n}_s = \left[\exp\left(\hbar\omega_s/k_BT\right)-1\right]^{-1} \qquad \bar{n}_v = \left[\exp\left(\hbar\omega_v/k_BT\right)-1\right]^{-1} \qquad (7.3a)$$

or

$$\frac{\bar{n}+1}{\bar{n}} = \exp\left(\hbar\omega/k_BT\right) \qquad (7.3b)$$

and the electron-phonon couplings (Huang-Rhys parameters) are $S_s = \lambda_s/\hbar\omega_s$ and $S_v = \lambda_v/\hbar\omega_v$. Note that $\hbar\omega = h\nu$. The frequency ω_s corresponds to a solvent mode, or a group of solvent modes with identical frequencies, and may take values close to 10 cm^{-1}. Even at the lowest temperatures studied in this work (T>188 K), $k_BT >> \hbar\omega_s$ and the following limits apply[22,23]

$$\sqrt{\bar{n}_s\left(\bar{n}_s+1\right)} \rightarrow k_BT/\hbar\omega_s$$
$$2\sqrt{\bar{n}_s\left(\bar{n}_s+1\right)}-\left(2\bar{n}_s+1\right) \rightarrow \hbar\omega_s/4k_BT \qquad (7.4)$$
$$I_{|p(m)|}(z) \rightarrow \frac{1}{\sqrt{2\pi z}}\exp\left[z-\frac{\left[p(m)\right]^2}{2z}\right]$$

where an asymptotic expansion of Bessel function for the solvents modes was made because of the large values of its argument. This asymptotic limit corresponds to a Gaussian line shape function, which supports a classical treatment of the medium modes.

When $S_s \rightarrow 0$ (i.e., $\lambda_s \rightarrow 0$) and only m = 0 contributes in Eq. (7.2), a simple relation was derived[22]

$$FCWD = \frac{1}{\hbar\omega_s}\exp\left[-S_v\left(2\bar{n}_v+1\right)\right]\left(\frac{\bar{n}_v+1}{\bar{n}_v}\right)^{p/2} I_p\left(2S_v\sqrt{\bar{n}_v\left(\bar{n}_v+1\right)}\right) \qquad (7.5a)$$

or

$$k = \frac{2\pi}{\hbar}\left|V^2\right|\frac{1}{\hbar\omega_s}\exp\left[-S_v\left(2\bar{n}_v+1\right)\right]\left(\frac{\bar{n}_v+1}{\bar{n}_v}\right)^{p/2} I_p\left(2S_v\sqrt{\bar{n}_v\left(\bar{n}_v+1\right)}\right) \qquad (7.5b)$$

where $p = \Delta E / \hbar\omega_s$.

A low temperature approximation can be made when $k_B T << \hbar\omega_v$, $n_v \to 0$. For vibrational frequencies of 1500 cm^{-1}, this approximation applies to the range of temperatures studied in this work (188-323 K), and the limiting form of the Bessel function for small values of the argument can be used

$$I_p(z) \to \frac{1}{p!}\left(\frac{z}{2}\right)^p \quad (7.6)$$

Using these approximations, Eq. (7.5) simplifies to

$$FCWD = \frac{1}{\hbar\omega_s}\exp\left[-S_v\right]\frac{S_v^p}{p!} \quad (7.7a)$$

or

$$k = \frac{2\pi}{\hbar}\left|V^2\right|\frac{1}{\hbar\omega_s}\exp\left[-S_v\right]\frac{S_v^p}{p!} \quad (7.7b)$$

Using the relation between the angular frequency of oscillation and the harmonic force constant of the vibrational mode, $\omega_v = \sqrt{f/\mu}$, and the relation of the molecular vibrational reorganization energy (λ_v) with the horizontal displacement of the vibrational mode between the initial and the final state, $\lambda_v = fd^2/2$, it is also useful to write

$$S_v = \frac{d\sqrt{\lambda_v \mu}}{\hbar\sqrt{2}} \quad (7.8)$$

It is easy to show that when Eq. (7.7) is used for a symmetrical reaction (ΔE=0, p=0, $\Delta E^{\ddagger}=\lambda_v/4$), and Eq. (7.8) is used for S_v, the ET rate is given by [24]

$$k = \nu \exp\left[-\frac{d\sqrt{2\mu\Delta E^{\ddagger}}}{\hbar}\right] \quad (7.9)$$

Eq. (7.9) was first derived by Formosinho, who presented it in a more general form, also valid for exothermic reactions[25]

$$k = \nu \exp\left[-\frac{\Delta x\sqrt{2\mu\Delta E^{\ddagger}}}{\hbar}\right] \quad (7.10)$$

where the barrier width Δx is the horizontal distance between the turning points of vibration of the oscillator in the initial and final states of a radiationless transition. For displaced harmonic oscillators with the same frequency in the initial and final states,

$$\Delta x = d - \sqrt{\frac{2|\Delta E|}{f}} \quad (7.11)$$

The calculation of the ET rate using the equations above relies on estimates of the normal mode displacement d, which is related to the change of the equilibrium positions Δq_i of the normal modes (i.e. to the bond-length changes due to electron transfer), and on the corresponding normal mode harmonic frequencies, to calculate the molecular vibrational reorganization energy

$$\lambda_v = \frac{1}{2}\sum_i \mu_i \omega_i^2 (\Delta q_i)^2 \quad (7.12)$$

In the single high-frequency approximation used above, a weighted average over the strongly coupling modes ω_v is employed rather than a normal mode frequency. The bond length changes for the donor or the acceptor may obtained from the mean square displacement[25-27]

$$d_{D/A} = \left(\sum_i (\Delta q_i)^2\right)^{1/2} \quad (7.13)$$

and the sum of the displacements of donor and acceptor is $d = d_A + d_D$. The value of d can be obtained from the equation above when the changes in bond lengths are known. Electronic structure calculations, described in Section 2, are often used to calculate the equilibrium structures of donor and acceptor before and after the transfer of the electron, which provides the basis to calculate each Δq_i relevant for Eq. (7.13). Table S1 presents the bond lengths of the donor and acceptor oscillators before and after the transfer of the electron and the corresponding values of Δq_i. According to Eq. (7.13) the displacement of the vibrational mode is d = 0.164 Å for charge separation and d = 0.162 Å for charge recombination reactions.

The displacement d can also be regarded as the sum of the displacements of the reactants and products from their equilibrium positions to their transition state configuration. According to the Intersecting-State Model (ISM)[28-31], the sum of these displacements is

$$d = \frac{a'\ln(2)}{n^\ddagger}\left(l_{r.eq} + l_{p,eq}\right) \quad (7.14a)$$

where a'=0.156 is a scaling factor and $n^\ddagger$ the transition state bond order. The ET reactions studied in this work do not involve bond-breaking or bond-forming processes, and $n^\ddagger$ is just the average bond order of the relevant bonds, i.e. $n^\ddagger$ = 1.75. The average bond lengths of CT or LE states in Table S1 are l_{eq} = 1.37 Å and for the ground state they are l_{eq} = 1.36 Å. Thus, ISM gives d = 0.169 Å, which is in good agreement with Eq. (7.13).

The major difference between ISM and estimates of d using Eq. (7.13) is that ISM accounts for a possible increase of the reorganization energy with the driving force of the reaction, expressed as

$$d = \frac{a'}{2n^\ddagger}\ln\left[\frac{1+g}{1-1/(1+g)}\right]\left(l_{r,eq} + l_{p,eq}\right), \qquad g = \exp\left(\sqrt{2n^\ddagger}\,\Delta G^0/\Lambda\right) \quad (7.14b)$$

where the parameter Λ regulates the dissipation of excess ΔG^0 by the accepting modes, that may lead to additional bond extensions not accounted by Eq. (7.13). This becomes especially relevant for very exothermic reactions.

The electronic frequency factor in Eqs. (7.9) and (7.10) corresponds to the pre-exponential factor in Eq. (7.7b), and is related to the non-adiabaticity of the ET reaction. It can be expressed as the electronic frequency in the donor, $\nu_{el} \approx 5\text{x}10^{14}$ s^{-1},[32] multiplied by a donor-acceptor distance-dependence factor

$$\nu = \nu_{el}\exp(-r_e\beta) \quad (7.15)$$

A simple electron-tunneling model (electron mass m_e) from donor to acceptor taking the spacer as a uniform square potential energy barrier with width r_e and a barrier height Φ_0, gives[33]

$$\beta = \frac{2}{\hbar}\sqrt{2m_e\Phi_0} \quad (7.16)$$

When the barrier is a material with an optical dielectric constant ε_{op} ($\varepsilon_{op} = n_D^2$, $n_D \approx 1.4$ on the basis of cyclohexane), it stabilizes the energy of the electron with respect to vacuum and the barrier Φ_0 is replaced by $\Phi = \Phi_0/n_D^2$.[30,34] In a charge recombination, the barrier height Φ_0 can be estimated from the absolute potential of the electron

$$\Phi_0 = E_{SCE} + E_{red}^D \quad (7.17)$$

where E_{SCE} = 4.71 eV and E_{red}^D = –1.69 eV. Hence, we have β = 1.27 $Å^{-1}$ and $\nu \approx 2\text{x}10^{11}$ s^{-1}.

All the parameters employed in ISM calculations using Eqs. (7.10) and (7.14b) were obtained from the properties of **1** except Λ. The value of this empirical parameter, which is relevant for very exothermic reactions, was taken from earlier application of ISM to similar systems, Λ = 70 kcal/mol.[29,35,36]

For the sake of completeness, it can also be demonstrated that from Eq. (7.2) it possible to derive

$$FCWD = \frac{1}{\sqrt{4\pi\lambda_s k_B T}} \sum_{n=0}^{\infty} e^{-S} \frac{S^n}{n!} \exp\left[-\frac{\left(\Delta G^0 + \lambda_s + nh\nu_v\right)^2}{4\lambda_s k_B T} \right] \tag{21}$$

when the solvent modes are treated classically and the vibrational modes are quantized[22]. A possible limitation of this approach to very exothermic ET reactions is the neglect of anharmonicity in the intramolecular modes. A significantly flatter dependence on exoergicity is predicted for the maximum rates using anharmonic modes[37], but for very exothermic reactions the steep decrease of the rates with the exoergicity is maintained and does not explain the lifting of exothermic rate restrictions.

Supplementary Note 8

Transient absorption spectra

(All the spectra presented in this section were subject to the subtraction of the solvent response and chirp correction)

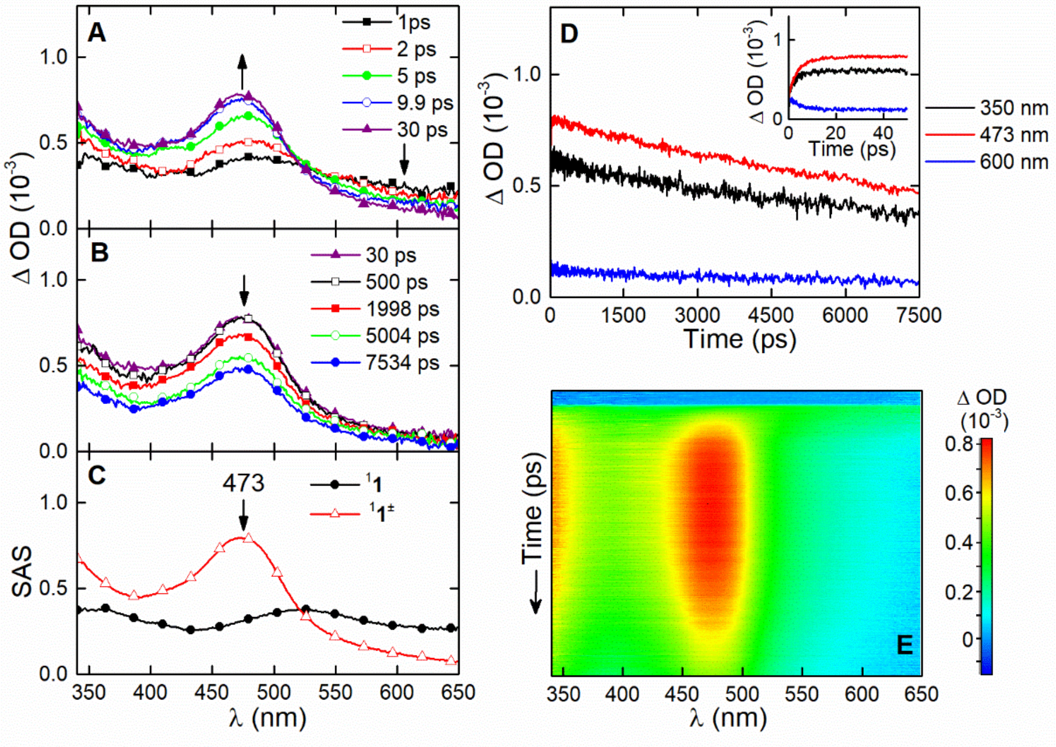

Supplementary Figure 14. Transient absorption spectra of **1** in dichloromethane at 293K, in short (A) and long time scales (B) and calculated Species-Associated Spectra (C). Time profile of selected decays (D). 2D image of collected data (E).

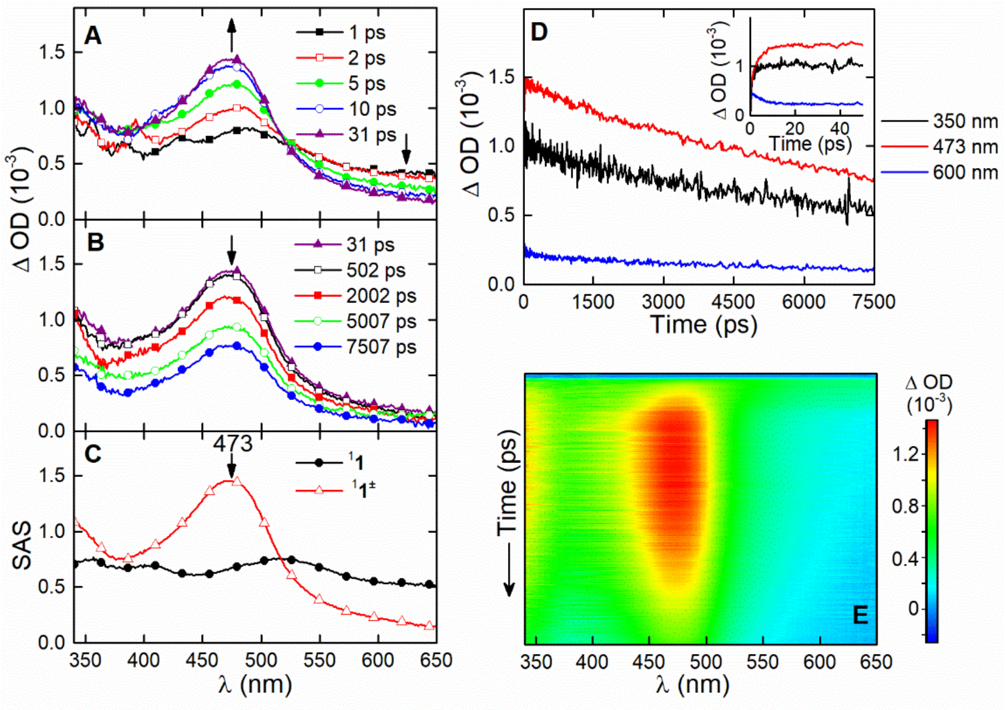

Supplementary Figure 15. Transient absorption spectra of **1** in dichloromethane at 276K, in short (A) and long time scales (B) and calculated Species-Associated Spectra (C). Time profile of selected decays (D). 2D image of collected data (E).

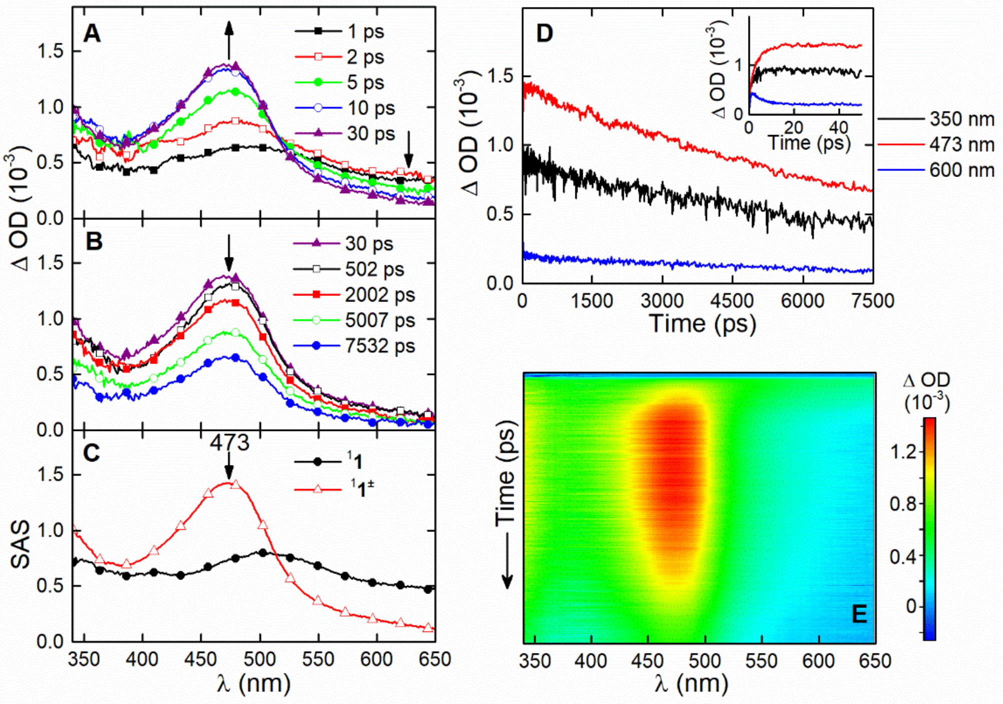

Supplementary Figure 16. Transient absorption spectra of **1** in dichloromethane at 253K, in short (A) and long time scales (B) and calculated Species-Associated Spectra (C). Time profile of selected decays (D). 2D image of collected data (E).

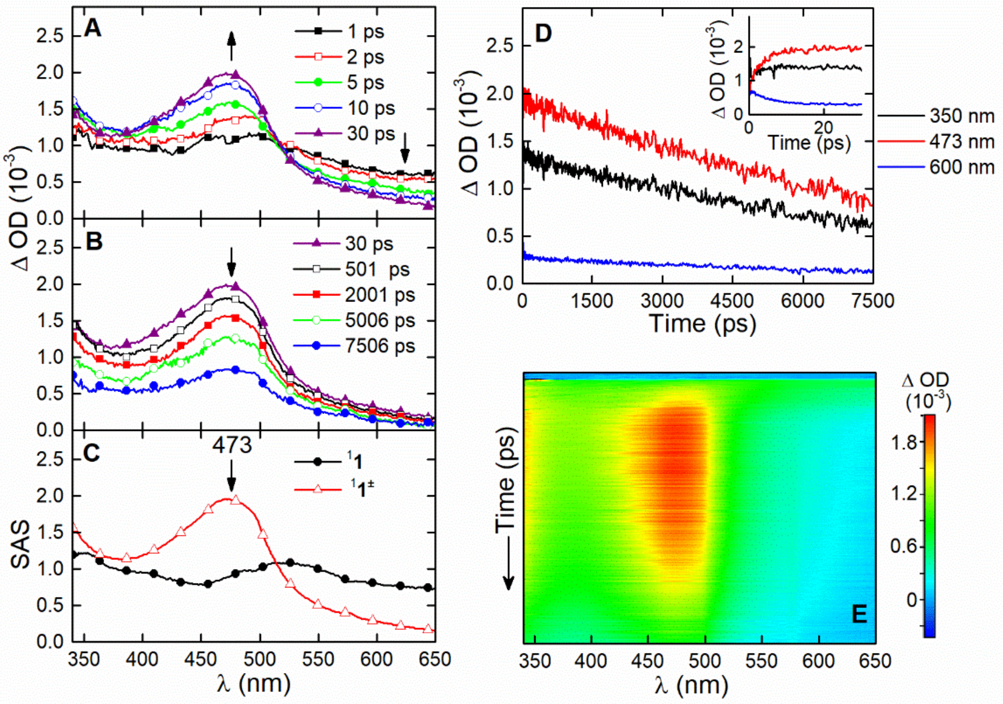

Supplementary Figure 17. Transient absorption spectra of **1** in dichloromethane at 223K, in short (A) and long time scales (B) and calculated Species-Associated Spectra (C). Time profile of selected decays (D). 2D image of collected data (E).

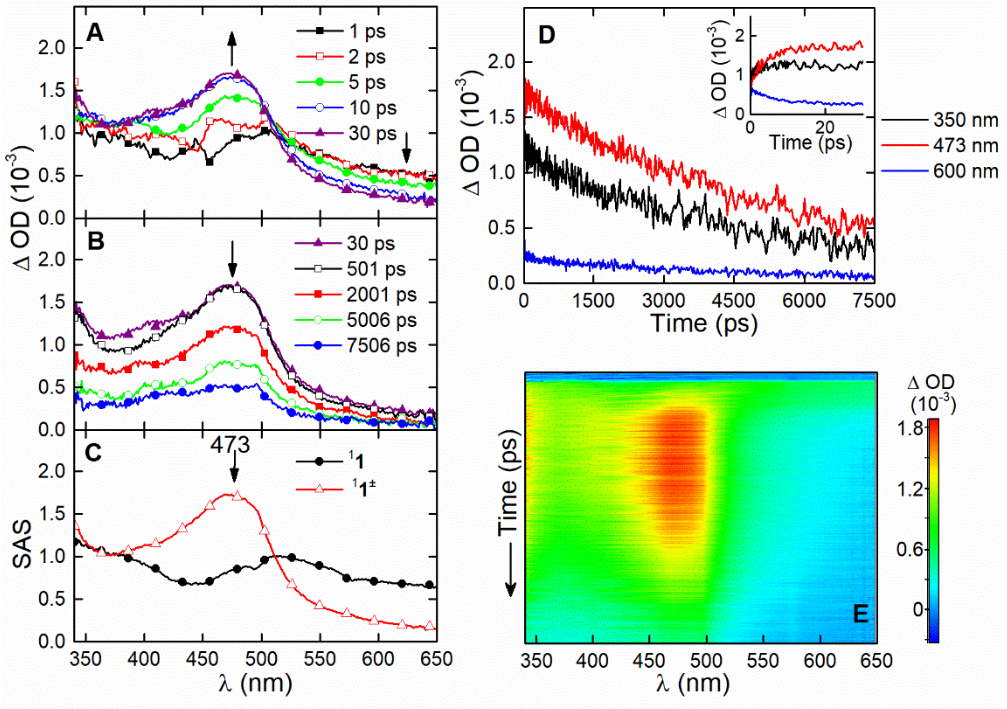

Supplementary Figure 18. Transient absorption spectra of **1** in dichloromethane at 188K, in short (A) and long time scales (B) and calculated Species-Associated Spectra (C). Time profile of selected decays (D). 2D image of collected data (E).

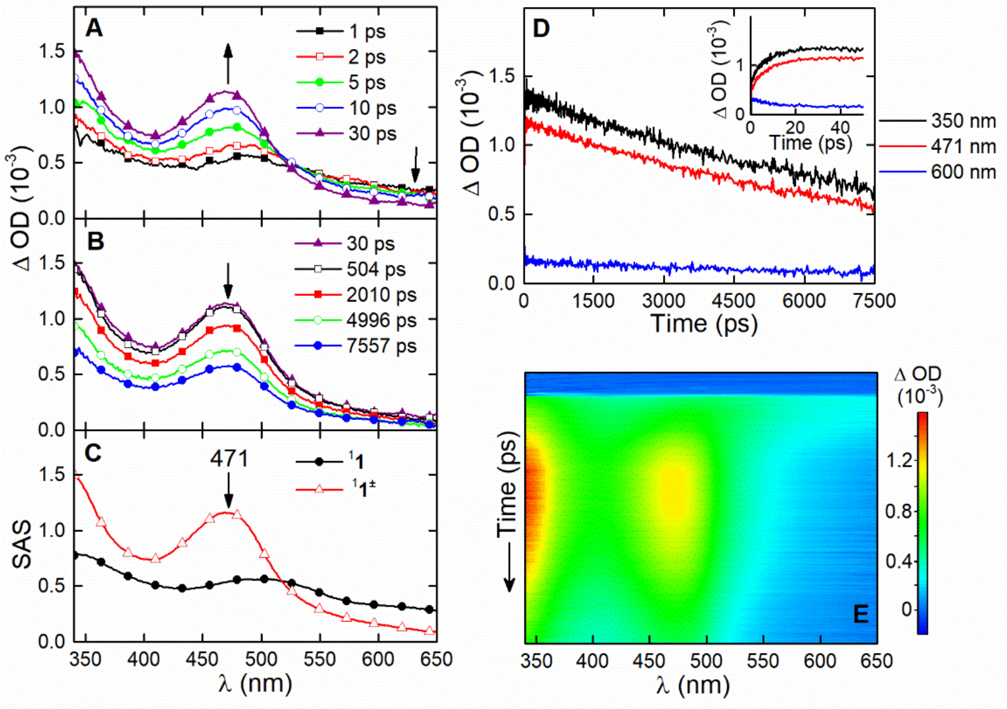

Supplementary Figure 19. Transient absorption spectra of **1** in ethyl acetate at 293K, in short (A) and long time scales (B) and calculated Species-Associated Spectra (C). Time profile of selected decays (D). 2D image of collected data (E).

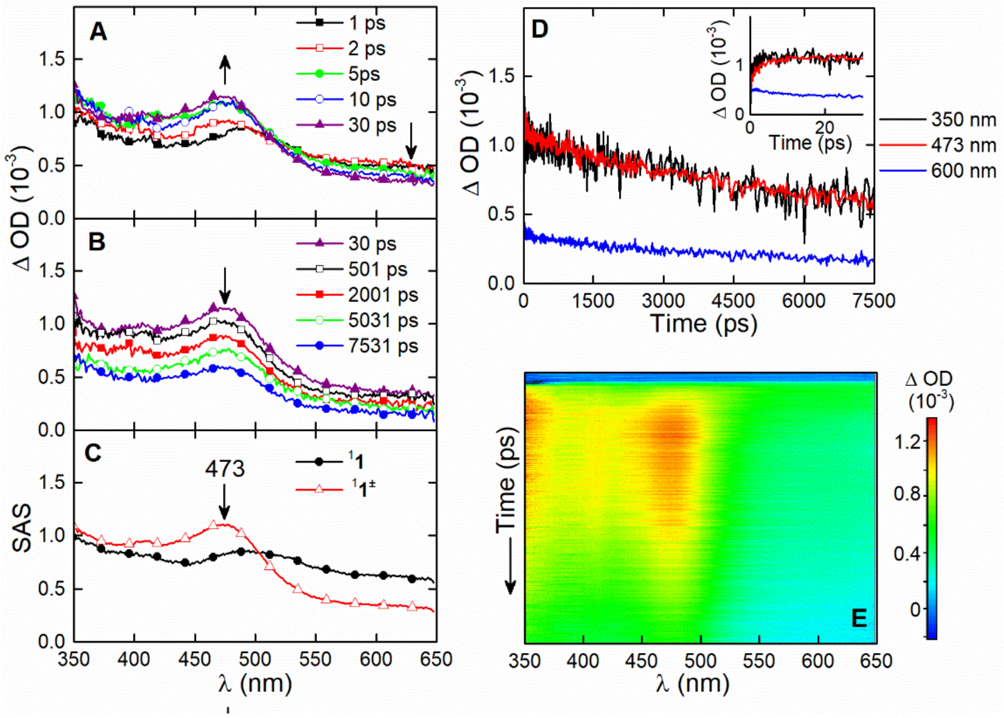

Supplementary Figure 20. Transient absorption spectra of **1** in chloroform at 323K, in short (A) and long time scales (B) and calculated Species-Associated Spectra (C). Time profile of selected decays (D). 2D image of collected data (E).

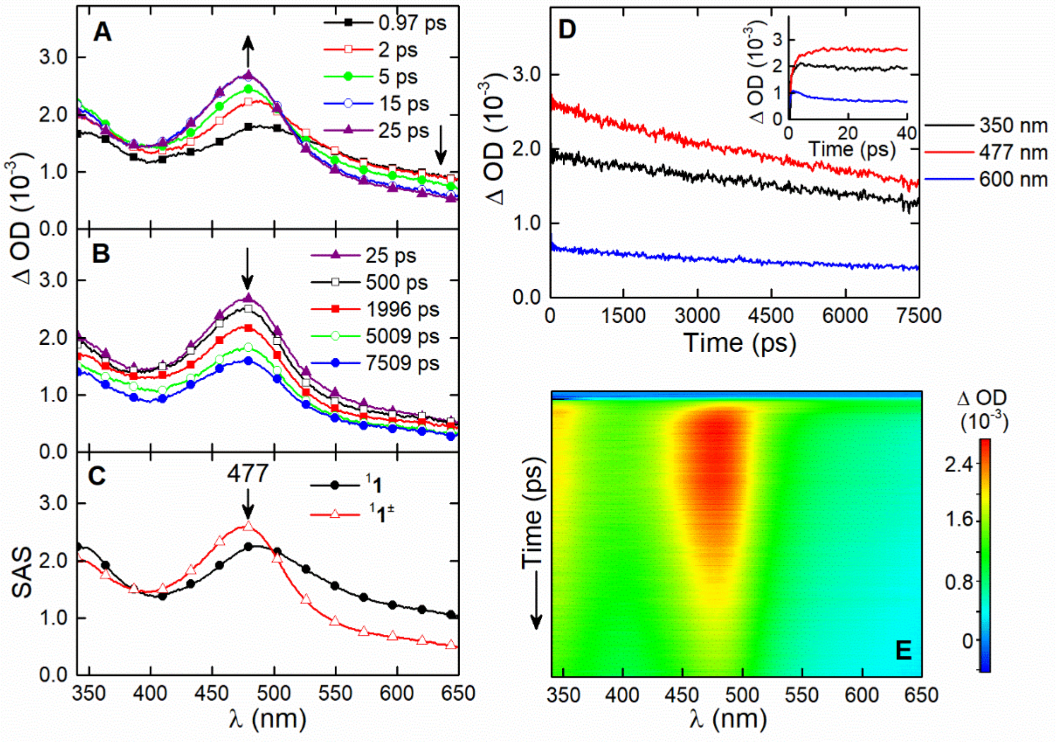

Supplementary Figure 21. Transient absorption spectra of **1** in chloroform at 293K, in short (A) and long time scales (B) and calculated Species-Associated Spectra (C). Time profile of selected decays (D). 2D image of collected data (E).

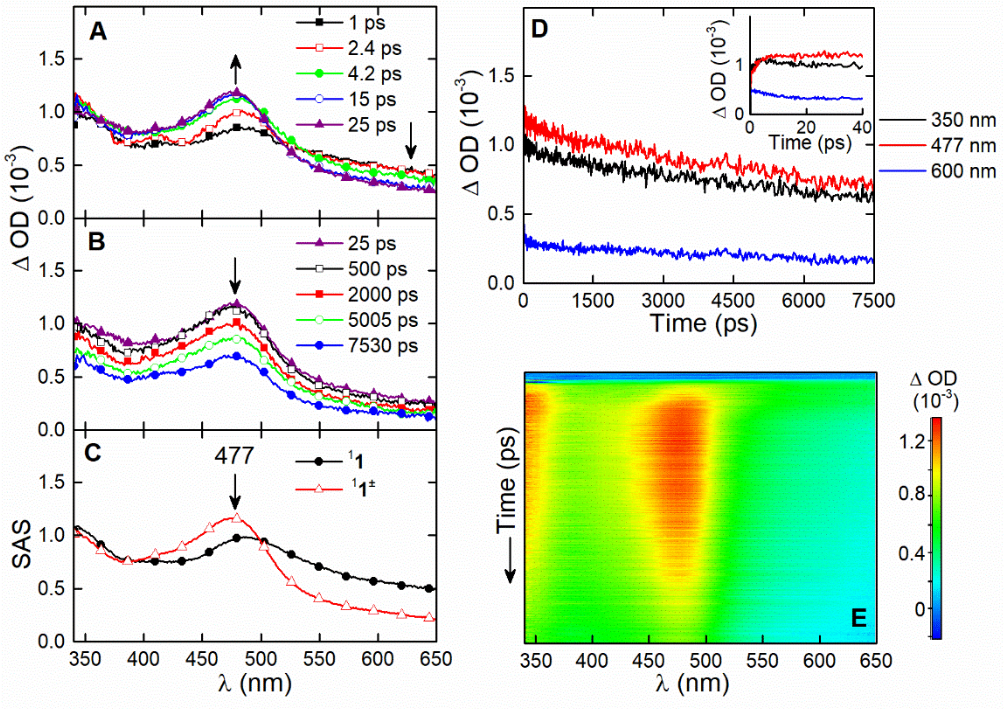

Supplementary Figure 22. Transient absorption spectra of **1** in chloroform at 276K, in short (A) and long time scales (B) and calculated Species-Associated Spectra (C). Time profile of selected decays (D). 2D image of collected data (E).

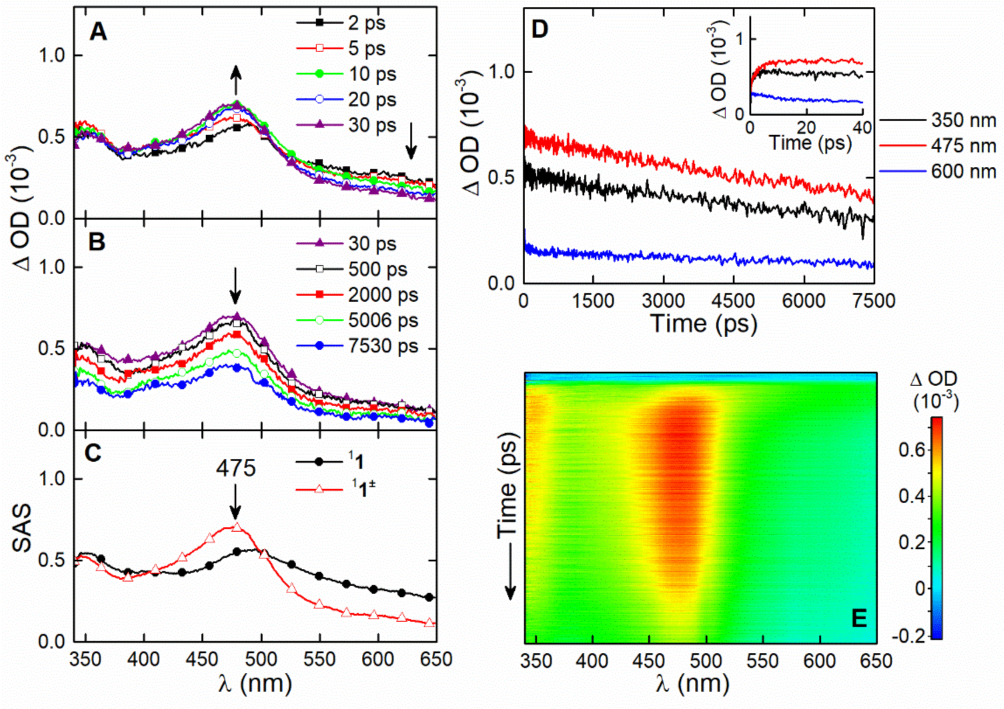

Supplementary Figure 23. Transient absorption spectra of **1** in chloroform at 253K, in short (A) and long time scales (B) and calculated Species-Associated Spectra (C). Time profile of selected decays (D). 2D image of collected data (E).

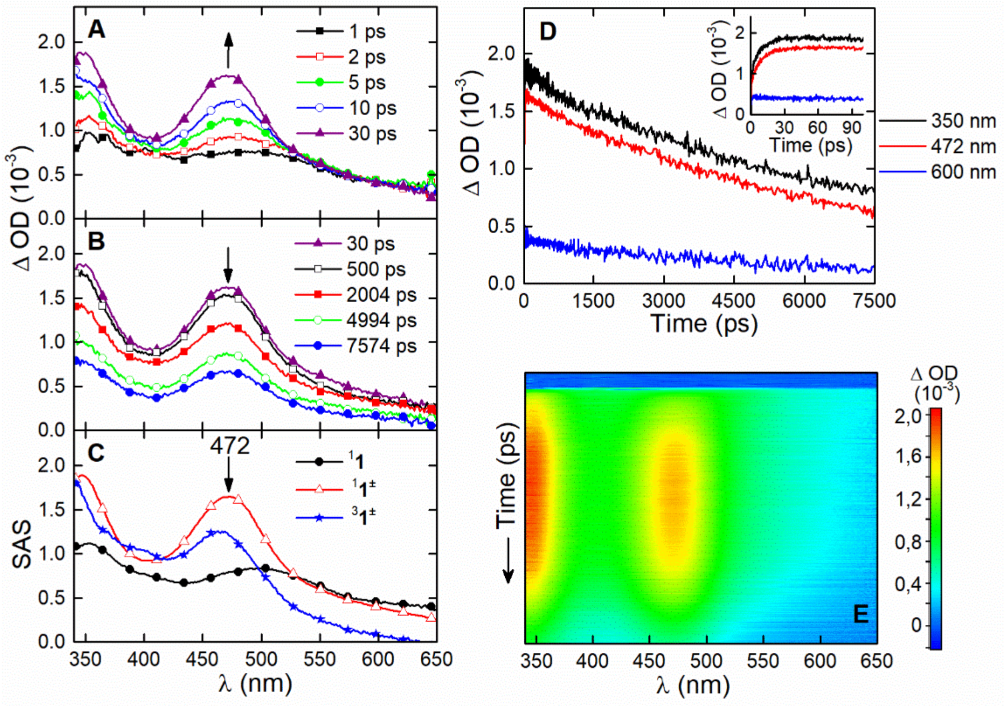

Supplementary Figure 24. Transient absorption spectra of **1** in isopropyl ether at 293K, in short (A) and long time scales (B) and calculated Species-Associated Spectra (C). Time profile of selected decays (D). 2D image of collected data (E).

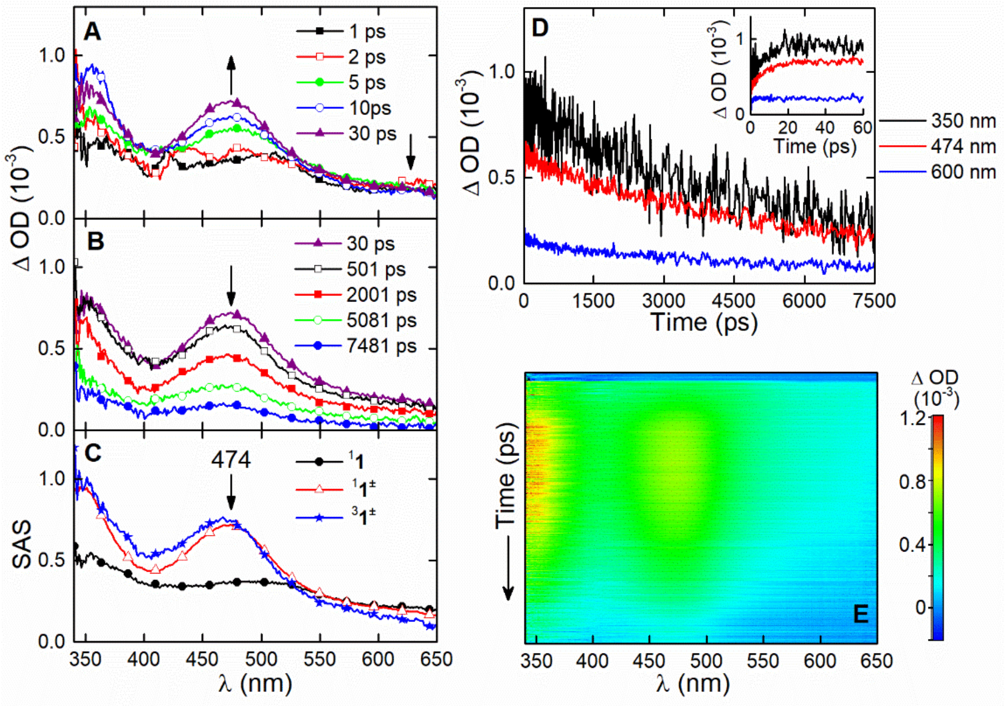

Supplementary Figure 25. Transient absorption spectra of **1** in di-n-butyl ether at 323K, in short (A) and long time scales (B) and calculated Species-Associated Spectra (C). Time profile of selected decays (D). 2D image of collected data (E).

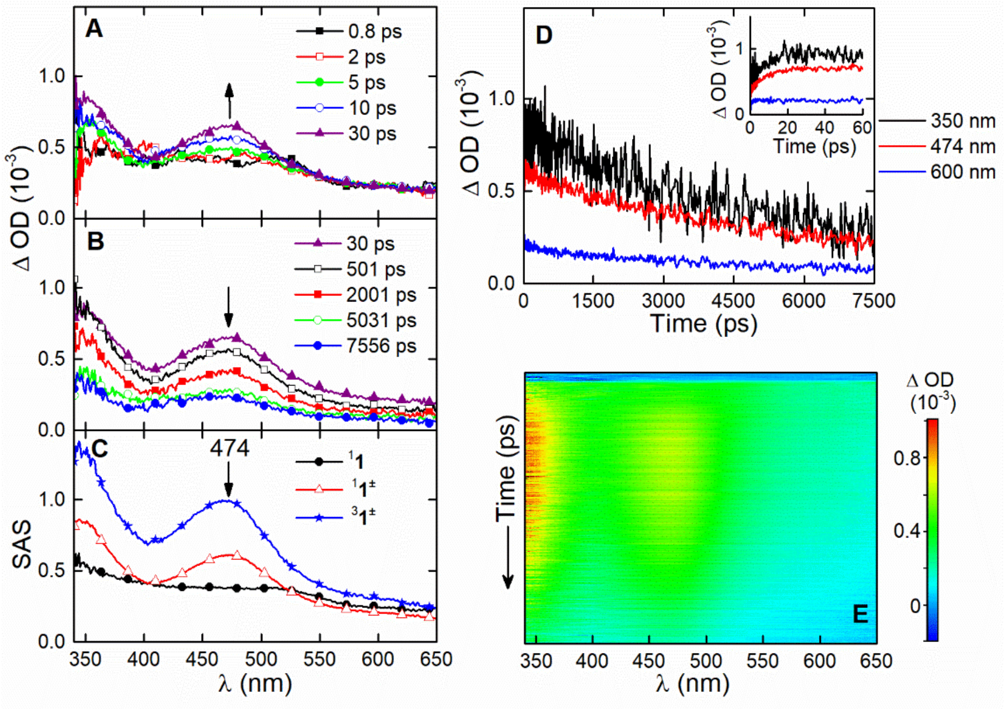

Supplementary Figure 26. Transient absorption spectra of **1** in di-n-butyl ether at 308K, in short (A) and long time scales (B) and calculated Species-Associated Spectra (C). Time profile of selected decays (D). 2D image of collected data (E).

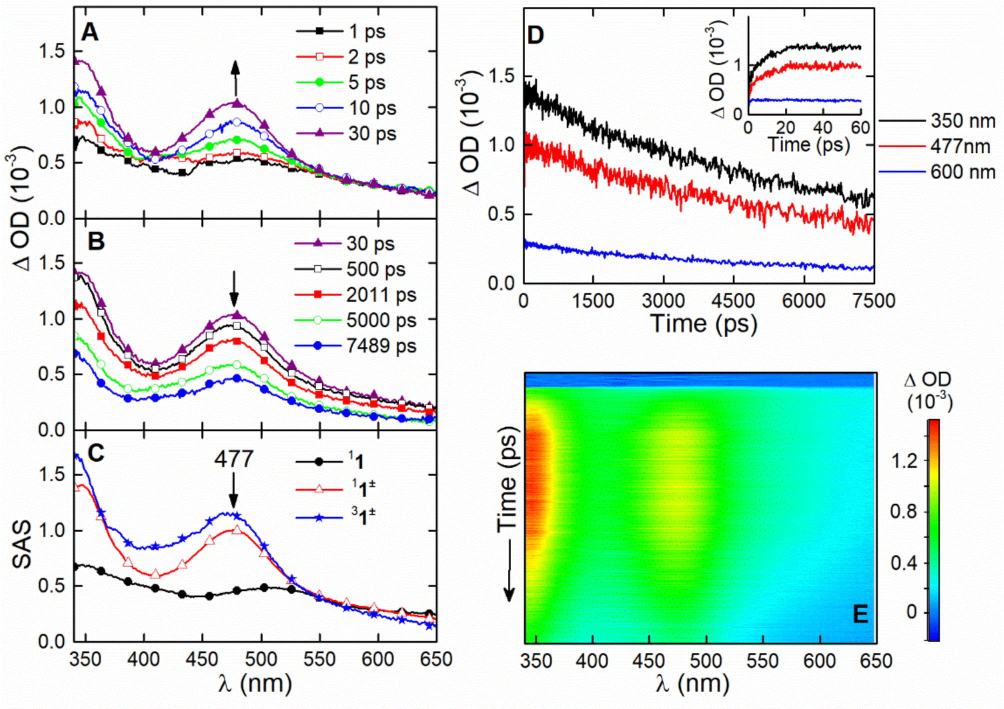

Supplementary Figure 27. Transient absorption spectra of **1** in di-n-butyl ether at 293K, in short (A) and long time scales (B) and calculated Species-Associated Spectra (C). Time profile of selected decays (D). 2D image of collected data (E).

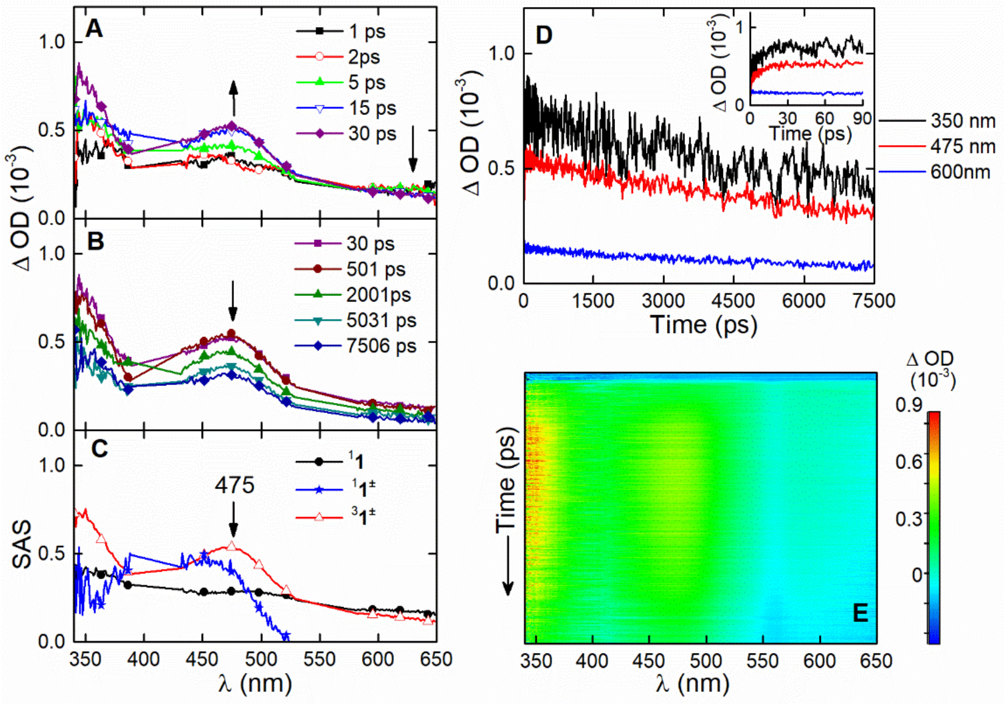

Supplementary Figure 28. Transient absorption spectra of **1** in di-n-butyl ether at 276K, in short (A) and long time scales (B) and calculated Species-Associated Spectra (C). Time profile of selected decays (D). 2D image of collected data (E).

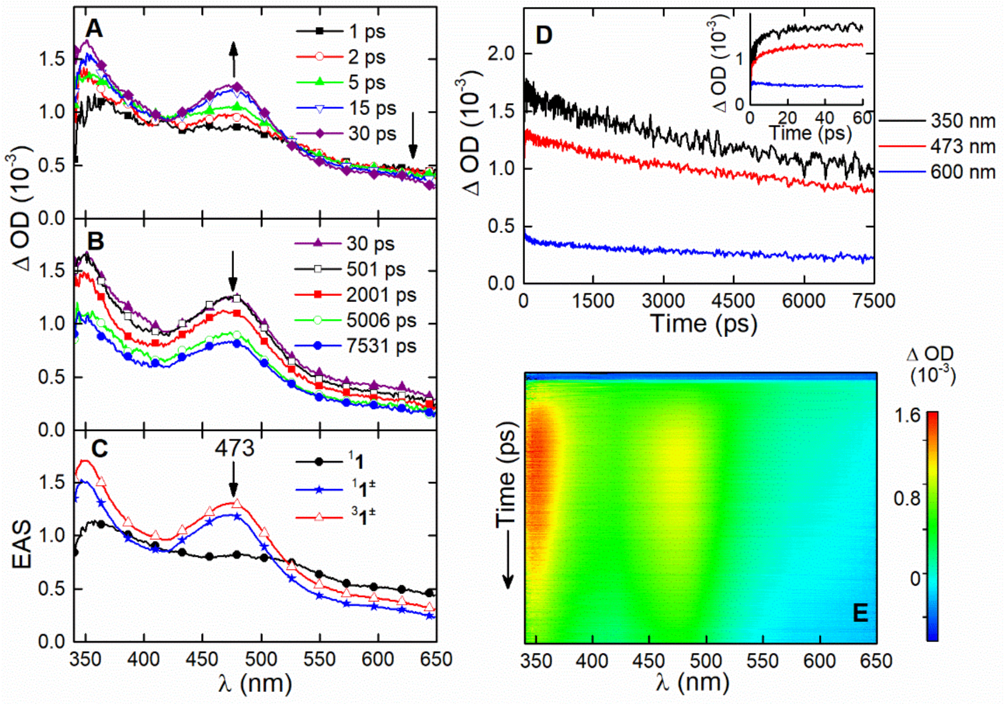

Supplementary Figure 29. Transient absorption spectra of **1** in di-n-butyl ether at 253K, in short (A) and long time scales (B) and calculated Evolution-Associated Spectra (C). Time profile of selected decays (D). 2D image of collected data (E).

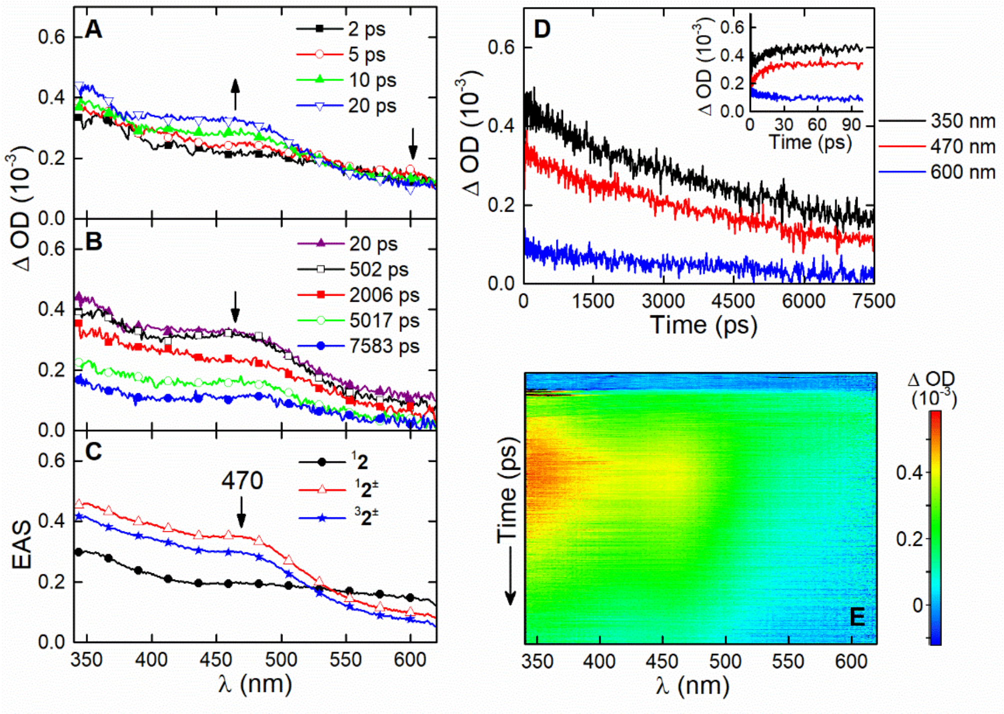

Supplementary Figure 30. Transient absorption spectra of **2** in dichloromethane at 293K, in short (A) and long time scales (B) and calculated Evolution-Associated Spectra (C). Time profile of selected decays (D). 2D image of collected data (E).

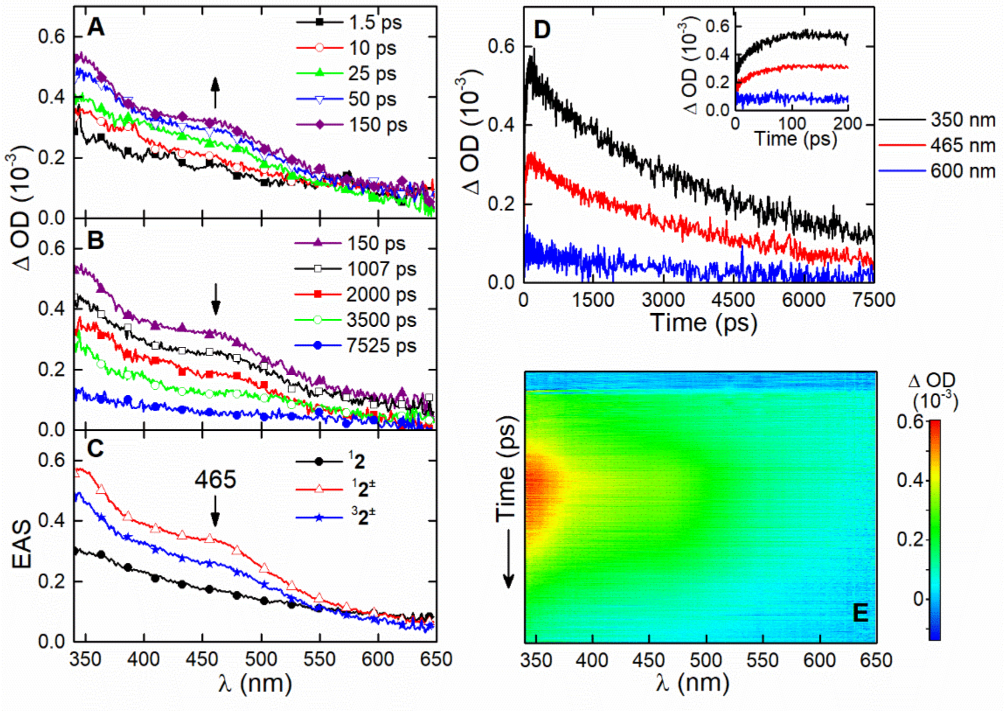

Supplementary Figure 31. Transient absorption spectra of **2** in ethyl acetate at 293K, in short (A) and long time scales (B) and calculated Evolution-Associated Spectra (C). Time profile of selected decays (D). 2D image of collected data (E).

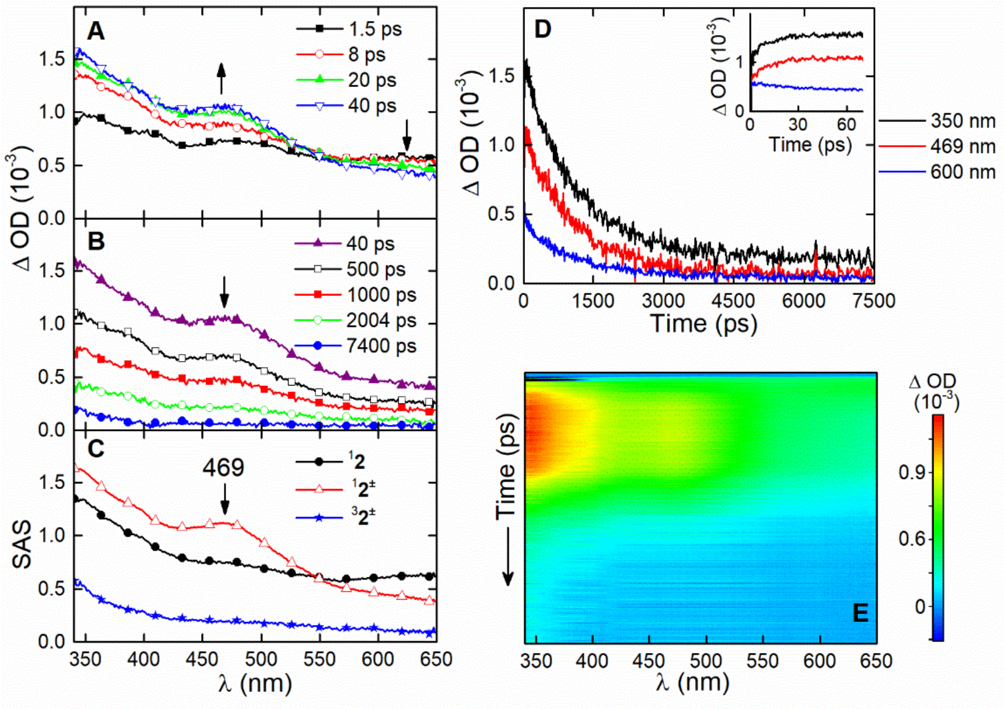

Supplementary Figure 32. Transient absorption spectra of **2** in chloroform at 293K, in short (A) and long time scales (B) and calculated Species-Associated Spectra (C). Time profile of selected decays (D). 2D image of collected data (E).

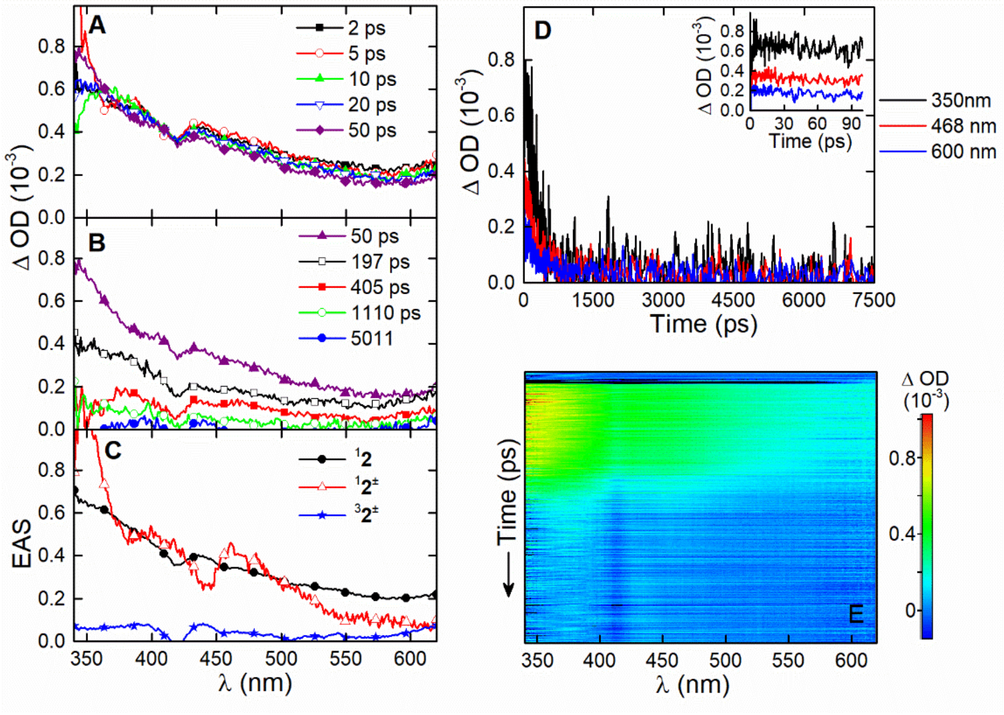

Supplementary Figure 33. Transient absorption spectra of **2** in di-n-butyl ether at 293K, in short (A) and long time scales (B) and calculated Evolution-Associated Spectra (C). Time profile of selected decays (D). 2D image of collected data (E).

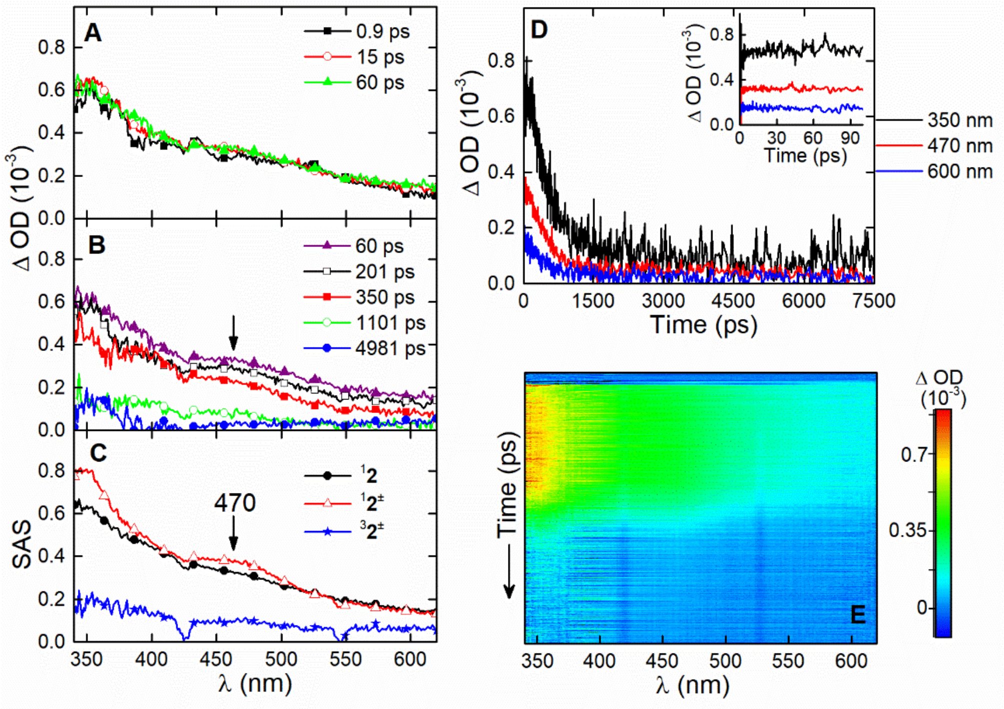

Supplementary Figure 34. Transient absorption spectra of **2** in di-n-butyl ether at 253K, in short (A) and long time scales (B) and calculated Species-Associated Spectra (C). Time profile of selected decays (D). 2D image of collected data (E). The lifetimes of species $^{1}\mathbf{2}$, $^{1}\mathbf{2}^{\pm}$ and $^{3}\mathbf{1}^{\pm}$ are 144 ps, 326 ps and larger than 10 ns, respectively

Supplementary References

1 Montalti, M., Credi, A., Prodi, L. & Gandolfi, M. T. *Handbook of Photochemistry*. 3rd edn, (CRC Press, 2006).

2 Lippert, E. *Z. Naturforsch.* **61**, 962 (1955).

3 Mataga, N., Kaifu, Y. & Koizumi, M. *Bull. Chem. Soc. Jpn.* **28**, 690 (1955).

4 Pasman, P., Mes, G. F., Koper, N. W. & Verhoeven, J. W. Solvent effects on photoinduced electron transfer in rigid, bichromophoric systems. *J. Am. Chem. Soc.* **107**, 5839-5843 (1985).

5 Weller, A. Photoinduced ET in Solution: Exciplex and RIP Formation Enthalpies and their Solvent Dependence. *Z. Physik. Chem. N. F.* **133**, 93-98 (1982).

6 Oevering, H. *et al.* Long-range photoinduced through-bond electron transfer and radiative recombination via rigid nonconjugated bridges: Distance and solvent dependence. *J. Am. Chem. Soc.* **109**, 3258-3269 (1987).

7 Wohlfahrt, C. in *Static Dielectric Constants of Pure Liquids and Binary Liquid Mixtures* 5-228 (Landolt-Börnstein, 1991).

8 Zweig, A., Hodgson, W. G. & Jura, W. H. The oxidation of methoxybenzenes. *J. Am. Chem. Soc.* **86**, 4124-4129 (1964).

9 Merkel, P. B., Luo, P., Dinnocenzo, J. P. & Farid, S. Accurate oxidation potentials of benzene and biphenyl derivatives via electron-transfer equilibria and transient kinetics. *J. Org. Chem.* **74**, 5163-5173 (2009).

10 Pasman, P., Verhoeven, J. W. & de Boer, T. J. Fluorescence of intramolecular electron donor-acceptor systems: The importance of through-bond interaction. *Chem. Phys. Lett.* **59**, 381-385 (1978).

11 Lim, B. T., Okajima, S., Chandra, A. K. & Lim, E. C. Radiationless transitions in electrondonor-acceptor complexes: Selection rules for S1→T1 intersystems crossing and efficient S1→S0 internal conversion. *Chem. Phys. Lett.* **79**, 22-27 (1981).

12 Okada, T. *et al.* Ultrafast intersystems crossing in some intramolecular heteroexcimers. *J. Phys. Chem.* **85**, 3957-3960 (1981).

13 van Willingen, H., Jones II, G. & Farahat, M. S. Time-resolved EPR study of photoexcited triplet-state formation in electron-donor-substituted acridinium ions. *J. Phys. Chem.* **100**, 3312-2216 (1996).

14 Dance, Z. E. X. *et al.* Time-resolved EPR studies of photogenerated radical ion pairs separated ny p-phenylene oligomers and of triplet states resulting from charge recombination. *J. Phys. Chem. B* **110**, 25163-25173 (2006).

15 Dance, Z. E. X. *et al.* Intersystem crossing mediated by photoinduced intramolecular charge transfer: Julolidine-anthracene molecules with perpendicular π systems. *J. Phys. Chem. A* **112**, 4194-4201 (2008).

16 Weller, A., Staerk, H. & Treichel, R. Magnetic-field effects on geminate radical-pair recombination. *Faraday Discuss. Chem. Soc.* **78**, 271-278 (1984).

17 Werner, H.-J., Staerk, H. & Weller, A. Solvent, isotope and magnetic field effects in the geminate recombination of radical ion pairs. *J. Chem. Phys.* **68**, 2419-2426 (1978).

18 van der Boom, T. *et al.* Charge transport in photofunctional nanoparticles self-assembled from zinc 5,10,15,20-tetrakis(perylenediimide)porphyrin buildong blocks. *J. Am. Chem. Soc.* **124**, 9582-9590 (2002).

19 Paddon-Row, M. N. & Shephard, M. J. A time-dependent density functional study of the singlet-triplet energy gap in charge-separated states of rigid bichromophoric molecules. *J. Phys. Chem. A* **106**, 2935-2944 (2002).

20 Birks, J. B., Dyson, D. J. & Munro, I. H. "Excimer" fluorescence. II. Lifetime studies of pyrene solutions. *Proc. Royal Soc. A* **275**, 575-588 (1963).

21 Crowther, A. C., Carrier, S. L., Preston, T. J. & Crim, F. F. Time-resolved studies of CN radical reactions and the role of complexes in solution. *J. Phys. Chem. A* **112**, 12081-12089 (2008).
22 Jortner, J. Temperature dependent activation energy for electron transfer between biological molecules. *J. Chem. Phys.* **64**, 4860-4867 (1976).
23 DeVault, D. Quantum mechanical tunneling in biological systems. *Quart. Rev. Biophys.* **13**, 387-564 (1980).
24 Jortner, J. & Ulstrup, J. Tunnelling in low-temperature atom-transfer processes. *Chem. Phys. Lett.* **63**, 236-239 (1979).
25 Formosinho, S. J. Quantum mechanical tunnelling in the radiationless transitions of large molecules. *J. Chem. Soc., Faraday Trans. 2* **70**, 605-620 (1974).
26 McCoy, E. F. & Ross, I. G. Electronic states of aromatic hydrocarbons: The Franck-Condon principle and geometries in excited states. *Australian J. Chem.* **15**, 573-590 (1962).
27 Van Duyne, R. P. & Fischer, S. F. A nonadiabatic description of electron transfer reactions involving large free energy changes. *Chem. Phys.* **5**, 183-197 (1974).
28 Arnaut, L. G., Pais, A. C. C., Formosinho, S. J. & Barroso, M. Absolute Rate Calculations for Atom Abstractions by Radicals. Energetic, Structural and Electronic Factors. *J. Am. Chem. Soc.* **125**, 5236-5246 (2003).
29 Serpa, C. *et al.* Electron Transfers in Supercritical CO_2. Ultra-Exothermic Charge Recombinations at the End of the 'Inverted Region'. *Chem. Eur. J.* **12**, 5014-5023 (2006).
30 Formosinho, S. J., Arnaut, L. G. & Fausto, R. A critical assessment of classical and semi-classical models for electron transfer reactions in solution. *Prog. Reaction Kinetics* **23**, 1-90 (1998).
31 Arnaut, L. G. & Formosinho, S. J. Understanding Chemical Reactivity. The Case for Atom, Proton and Methyl Transfers. *Chem Eur. J.* **14**, 6578-6587 (2008).
32 Formosinho, S. J. & Arnaut, L. G. Electron-transfer reactions in organic chemistry. *Bull. Chem. Soc. Jpn.* **70**, 977-986 (1997).
33 Wenger, O. S. How donor-bridge-acceptor energetics influence electron tunneling dynamics and their distance dependences. *Acc. Chem. Res.* **44**, 25-35 (2010).
34 Arnaut, L. G. & Formosinho, L. G. Theoretical Studies of Intramolecular ET Reactions: Distance and Free-Energy Dependences. *J. Photochem. Photobiol. A: Chem.* **100**, 15-34 (1996).
35 Serpa, C., Gomes, P. J. S., Arnaut, L. G., Formosinho, S. J. & Seixas de Melo, J. Temperature dependence of ultra-exothermic charge recombinations. *ChemPhysChem* **7**, 2533-2539, doi:10.1002/cphc.200600467 (2006).
36 Arnaut, L. G., Formosinho, S. J. & Burrows, H. D. *Chemical Kinetics*. (Elsevier, 2007).
37 Yeganeh, S. & Ratner, M. A. Effects of anharmonicity on nonadiabatic electron transfer: A model. *J. Chem. Phys.* **124**, 044108 (2006).
