# Supplementary material for: Higher activation barriers can lift exothermic rate restrictions in electron transfer and enable faster reactions
Source: Nat Commun. 2018 Jul 25;9:2903. doi: 10.1038/s41467-018-05267-5 (PMC6060101; doi:10.1038/s41467-018-05267-5)
Supplement: Supplementary file 2 — Description of Additional Supplementary Files [file 41467_2018_5267_MOESM2_ESM.pdf]

Description of Additional Supplementary Files

File Name: Supplementary Movie 1
Description: Perspectives of the lowest energy conformations of molecule 1. Calculated with Marvin 17.2.27 2017 ChemAxon using the conformer plugin. All the conformers found differ by minor orientations of the methoxy group linked to the donor moiety, which are irrelevant for the electronic coupling between donor and acceptor moieties, and probably collapse into one single conformer with more accurate energy minimization procedures.
